# Camera Assisted Roadside Monitoring for Invasive Alien Plant Species Using Deep Learning

**DOI:** 10.3390/s21186126

**Published:** 2021-09-13

**Authors:** Mads Dyrmann, Anders Krogh Mortensen, Lars Linneberg, Toke Thomas Høye, Kim Bjerge

**Affiliations:** 1 Department of Electrical and Computer Engineering, Aarhus University, Finlandsgade 22, 8200 Aarhus N, Denmark; kbe@ece.au.dk; 2 Department of Agroecology, Aarhus University, Forsøgsvej 1, 4200 Slagelse, Denmark; anmo@agro.au.dk; 3 Danish Road Directorate, Thomas Helsteds Vej 11, 8660 Skanderborg, Denmark; lali@vd.dk; 4 Department of Bioscience and Arctic Research Centre, Aarhus University, Grenåvej 14, 8440 Rønde, Denmark; tth@bios.au.dk

**Keywords:** invasive alien plant species, machine learning, high speed acquisition, remote sensing, roadside

## Abstract

Invasive alien plant species (IAPS) pose a threat to biodiversity as they propagate and outcompete natural vegetation. In this study, a system for monitoring IAPS on the roadside is presented. The system consists of a camera that acquires images at high speed mounted on a vehicle that follows the traffic. Images of seven IAPS (*Cytisus scoparius*, *Heracleum*, *Lupinus polyphyllus*, *Pastinaca sativa*, *Reynoutria*, *Rosa rugosa*, and *Solidago*) were collected on Danish motorways. Three deep convolutional neural networks for classification (ResNet50V2 and MobileNetV2) and object detection (YOLOv3) were trained and evaluated at different image sizes. The results showed that the performance of the networks varied with the input image size and also the size of the IAPS in the images. Binary classification of IAPS vs. non-IAPS showed an increased performance, compared to the classification of individual IAPS. This study shows that automatic detection and mapping of invasive plants along the roadside is possible at high speeds.

## 1. Introduction

Invasive alien plant species (IAPS) have been identified as a growing threat to global sustainability [1,2], due to human activity. IAPS are considered to be a major driver of biodiversity loss. Invasive species are among the top five threats to biodiversity worldwide [3]. They pose a threat to native species, their habitats, and the functioning of the ecosystem. Invasive species can also adversely affect human health and the economy [4]. Monitoring programs for IAPS are needed to identify where IAPS are most abundant, most likely to spread, or most easily contained [5]. Transportation lines such as roads, railways, and trails are critical dispersal routes for IAPS since traffic can transport seeds over long distances [6,7]. Efficient and cost-effective monitoring for IAPS along, for example, roads will therefore serve as an important advance towards the mitigation of the negative consequences of IAPS.

Remote sensing is increasingly being used for the detection of invasive plant species as reported by Bradley [8], and Huang and Asner [9]. Here, the spatial analysis of plant invasions is based on imagery acquired by satellites to create distribution maps of invasive plants to support decision making for management and control. Bolch et al. [10] focused on remote sensing capabilities to detect and monitor IAPS across terrestrial, riparian, aquatic, and human-modified ecosystems. The identification and remote detection of alien invasive plants in commercial forests are reported by Ismail et al. [11].

Unmanned aerial vehicles (UAVs) have been popular in remote sensing applications [12]. For monitoring IAPS, this approach offers a valuable solution for local areas with a high spatial and temporal resolution [13,14]. However, alien species that are distributed along roads are impractical to monitor using UAVs. Firstly, the large distances mean that the drones will have to be charged or refueled several times to cover the motorways. Secondly, UAVs are subject to regulation and they may not be flown unattended in Denmark, which makes it difficult to cover thousands of kilometers every week.

In many countries, the road network is already inspected by planned drivings. Our approach is to use these inspections to record pictures of plants taken along the road and construct a map with the occurrence of IAPS. Baard and Kraaij [15] demonstrated that roadside surveys can be used to facilitate an early detection of invasive plant species along 530 km of roads in the Garden Route, South Africa. McDougall et al. [16] used manual surveys of roadsides in seven regions worldwide to investigate non-native plants invading the mountain vegetation.

Niphadkar and Nagendra [17] conducted a survey on research that uses remote sensing combined with functional plant traits for the mapping and monitoring of invasive alien plants. Niphadkar and Nagendra [17] reported that morphological, biochemical, phenological, or physiological plant features can improve remote sensing mapping. Carlier et al. [18] presented an application of morphological image analysis to provide an objective method for detection and accurate cover assessment of an IAPS. They used top-down images captured using a hand-held digital camera with images that cover 1 m × 1 m. James and Bradshaw [19] based their work on images collected using UAVs, which allowed them to cover larger areas, but instead of using morphological image analysis, James and Bradshaw [19] used the U-net convolutional neural network to segment images semantically. The use of a convolutional neural network makes the method less affected by changes in light intensity and shadows and makes it applicable in images with plant occlusion.

In our work, we investigate whether it is possible to automate the registration of IAPS along the Danish state roads, including motorways. We use high-resolution images that are processed automatically to distinguish individual plants along the road. Since the roads are already inspected from a driving vehicle every week, a camera mounted on the vehicle that automatically scans the roadside is a labor- and cost-efficient tool. This is in contrast to, for example, manual surveys or cameras on UAVs, which would add an extra workflow.

We have tested camera equipment that can provide sufficient image quality so that it is possible to identify selected IAPS in the collected images at normal traffic speeds on the Danish motorways. The work includes images collection and annotation as well as applying deep learning algorithms for automatic detection of IAPS in the collected images. The paper demonstrates how detected IAPS can be presented on a map based on the collected data. Finally, the deep learning algorithms are evaluated and the challenges of monitoring IAPS are discussed.

In summary, this paper makes the following new contributions:

- Presentation of a camera solution to collect images of plants along the roadside at normal traffic speeds on the Danish motorways.
- Evaluation of deep learning algorithms for object detection and classification of invasive plants.
- Steps towards an automatic computer vision system for detection and mapping of IAPS in real-time.

## 2. Camera and Platform for Collecting Images

A prerequisite for mapping invasive plants automatically is to use camera equipment capable of recording images of sufficient quality to detect the plants. The detection must take place from a vehicle that follows the traffic on Danish motorways, that is, with speeds in the range of 100 km/h–130 km/h. Therefore, the camera should be able to take images with sufficiently fast exposure time to avoid motion blur, with a resolution that allows plants to be recognized, and with a frame rate that ensures the full roadside is covered.

The way the camera is mounted on the car also affects the object resolution, the required exposure time to avoid blur, and the required frame rate to cover the full roadside. This is because the distance to the roadside depends on the camera orientation and motion blur depends on the direction of movement relative to the direction of recording. The following sections will go through the camera choice and camera settings.

### 2.1. Ground Sampling Distance

**Figure 1:**
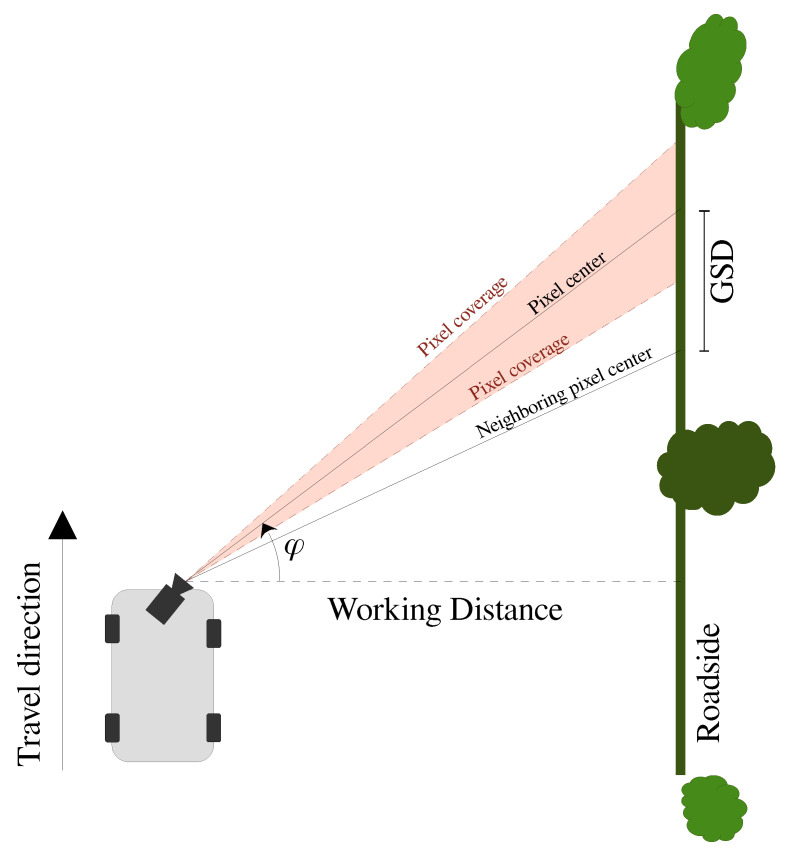
Ground sampling distance (GSD) at specific camera orientation relative to the driving direction, and thus the roadside. The red filled area illustrates the area covered by a single pixel. The closer the the orientation angle φ is to 90°and the greater the working distance (WD), the greater GSD. With a larger GSD, fewer details can be captured in the images, which is also illustrated in Figure 2.

The spatial resolution is defined as the distance on the object that is covered by the distance between neighboring pixels as illustrated in Figure 1. In remote sensing this unit is called ground sampling distance (GSD), since the camera is often pointed towards the ground and the spatial resolution becomes the ground distance between two pixels ([20], p. 31). The unit is meter per pixel. There are three main factors that affect the ground sampling distance. The first is the image sensor’s resolution and size. The second is the focal length of the lens. The third is the distance from camera to the object (roadside) along the camera’s primary axis (illustrated as ‘Pixel center’ in Figure 1). When the camera points in the direction of travel, the least amount of blur is obtained in the image, but at the same time, part of the image will be useless, as it does not cover the roadside. When the camera is pointing in the direction of travel, we get pictures that cover the largest possible area. However, this is at the expense of the ground sampling distance and, thus, the level of detail.

If the camera is shifted to point in between the direction of travel and perpendicular to the direction of travel, there will be a large variation in the size of a plant depending on the location in the image. It, therefore, places greater demands on the subsequent image processing, which must not only be invariant to size, due to the plants’ natural variation in size at different growth stages, but also invariant to different locations in the image.

When the camera points perpendicular to the direction of travel (i.e., φ=0 in Figure 1), the highest resolution of the objects (GSD) and the smallest variation in size of objects is ensured. On the other hand, the change in the content of each pixel during the exposure will be greatest with this orientation, thereby resulting in greater image blur. Higher demands are, therefore, put on the exposure time. Moreover, with an orientation perpendicular to the driving direction, the scanned area by each image will be the least. This orientation, therefore, also requires a high shutter speed and a high frame rate, which puts extra demands on the image processing platform.

**Figure 2:**
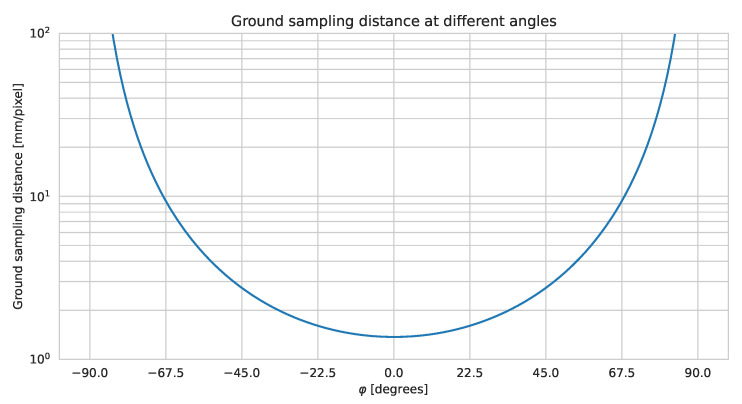
Ground sampling distance relative to camera angle, φ. φ = 0° corresponds to perpendicular to the driving direction. The calculations are based on a stationary camera. The effect of moving the camera is shown in Figure 3.

Figure 2 illustrates the relationship between the GSD and a pixel orientation relative to the direction of travel. This figure is based on a 7 m working distance, WD, corresponding to 1 m from car to the emergency track, 2.55 m emergency track, 2.5 m cut grass, and 1 m from the grass to the plant. The camera parameters are based on a Sony IMX226 sensor with an 8 mm lens, which provides a horizontal field-of-view of 46°. This setup is described in further details in a following section. Since a camera has a given field of view, the ground sampling distance will depend on the location in the image. However, when the distance to the object is large relative to the focal length of the lens, the difference between the GSD for the individual pixels will be small.

### 2.2. Motion Blur and Resolution

**Figure 3:**
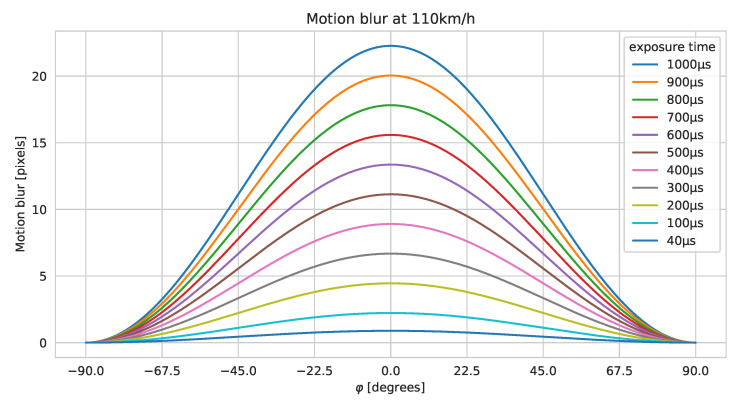
Motion blur at different orientations of center pixel in image when driving 110 km/h with a Sony IMX226 with a lens that ensures a 46° horizontal field of view.

Motion blur will acour when pictures are taken at speed. The amount of motion blur in an image relates to the shutter speed, field of view, and camera orientation relative to the object. Figure 3 shows the relationships between blur and orientation based on a Sony IMX226 sensor with an 8 mm lens. In the calculation, we assume that the lens is ideal and does not itself contribute to blur. The GSD is at its minimum when φ=0°, meaning we obtain the most details. At the same time, the amount of blur is also the greatest when moving the camera. A short exposure time is, therefore, preferable since it will bring down the amount of motion blur (at the expense of added noise if the gain is increased). Figure 3 shows that for our particular camera setup, having a shutter speed of 1/1000 s results in horizontal motion blur of more than 20 pixels. When the blur spreads over several horizontal pixels, it means that the horizontal resolution decreases. It, therefore, reduces the details captured in the images and thus, reduces the possibility of recognizing plants along the roadside. Ideally, the blur should be kept below 1 pixel. This is not practically possible without adding external lighting, due to the fast driving speed.

Plants that are far away will appear smaller than plants closer to the camera. As we want to reduce the variation in plants’ scaling to ease the automatic classification, we have chosen to prioritize a camera setup that points perpendicular to the roadside (i.e., φ=0°). Thus, the size of the plants is approximately independent of the location in the pictures.

### 2.3. Camera Setup

We tested a Logitech Brio, a Stereolabs ZED2, and two machine-vision cameras; a Daheng MER2-503-36U3C with a Sony IMX264 sensor and a DaDaheng MER-1220-32U3C with a Sony IMX226 sensor. The Stereolabs ZED2 camera was used in a study by Sousa Guedes et al. [21], where it was also mounted on a car and used for detecting road-killed amphibians and small birds. However, tests showed that the fastest exposure times of the Logitech and Stereolabs cameras are not sufficient for this application as plants were too blurred to be recognizable, when the cameras were filming perpendicular to the travel direction.

The Sony IMX264 sensor is fast enough for the application. Moreover, it is a global shutter sensor and therefore, does not suffer from the rolling-shutter distortion. However, the resolution is only 5 megapixels, whereas the IMX226 is 12 megapixels. By inspecting the images from the two machine vision cameras from Daheng with 8 mm and 16 mm lenses, it was estimated that the higher resolution of the IMX226 outweighed the rolling shutter distortion. Global shutter cameras with comparable or higher resolution exist, but the IMX226 delivered sufficient image quality for a person to easily recognize the plant species in question at a lower price than a global shutter camera with the same resolution. With the Daheng MER-1220-32U3C camera and an 8 mm lens, we get a field of view of 6.2 m at 7 m from the camera to the roadside (working distance). Full coverage of the roadside requires 161 images per km. At a speed of 110 km/h, the frame rate must be at least 4.91 FPS to achieve full coverage.

**Figure 4:**
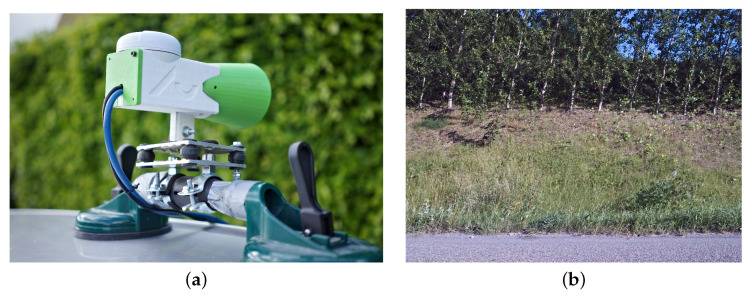
(**a**) The selected camera system that is tested at speeds up to 130 km/h. It consists of Daheng MER-1220-32U3C camera with 8mm lens and u-blox 8 UBX-M8030-KT GNSS receiver that are mounted on the car roof with suction cups. (**b**) Sample from Daheng MER-1220-32U3C with an 8 mm lens and 100 µs exposure time, while driving 110 km/h. Notice the leaning of the trees, which is a result of the rolling shutter.

Based on these considerations, we selected the Daheng MER-1220-32U3C camera with the Sony IMX226 sensor. The camera was mounted on the roof of the car and oriented perpendicular to the direction of travel (Figure 4a). The roads of interest in this study are the danish state roads, which typically have two to four lanes in each direction, separated by crash barriers. Therefore a single camera pointing to the right is sufficient in this study, although an additional camera pointing to the left may be added if the system were to be used on smaller roads with a clear view of the roadside on both sides of the road. This camera has a C-mount, which makes it possible to change the lens. In addition, it has the option of manually setting parameters, including shutter speed, analog gain, aperture and focusing distance. A sample image of the Daheng MER-1220-32U3C, taken at 1/10,000 s, is shown in Figure 4b. The picture shows that this camera can also provide high sharpness, so it is possible to distinguish individual leaves of grass. Even though the images are sharp they suffer from distortion due to the rolling shutter of the sensor. This phenomenon is seen in Figure 4b, where trees lean to the right, even though they are vertical in reality. However, not to a degree that is worse than if the plants were exposed to wind.

### 2.4. Camera Mount and Housing

The camera is mounted in a 3D printed housing with a lens hood, shown in Figure 4a. In addition to protecting the camera, this housing has a bracket for mounting, as well as space for mounting the GNSS receiver. The housing is mounted on a bracket that is attenuated with silicone pads to dampen vibrations. This bracket is mounted on a suction cup otherwise used for windshield replacement, which allows it to be mounted on any car. On top of the camera sits a GNSS receiver from Navilock with a u-blox 8 chipset. This GNSS has a 2.5 m positioning accuracy (Circular Error Probable) and a maximum refresh rate of 10 Hz when using the GPS and GALILEO satellites.

### 2.5. Processing Platform

With a camera that covers 6.2 m of the roadside in each image, we need 612,903 images to cover one roadside of the 3800 km Danish state-owned roads. Before these images provide value, it is necessary to go through them manually and annotate plants, to create a training basis for automated detection algorithms. To facilitate the manual annotation, we have developed a remote control that can be used to mark places with invasive plants while driving. The images are, therefore, only saved when the button has been activated. When driving approximately at 110 km/h, corresponding to 30 m/s, one may risk passing the invasive plants before the button is pressed. Therefore, a buffer was implemented that constantly contains images from the last second. By pressing the button, the image is thus saved back in time until the time that the button is activated. This means that if you first press the button shortly after passing an invasive plant species, it will still be recorded. The images are saved on a 1 TB Samsung Portable SSD.

A processing platform must be used to save the images and synchronize them with the GNSS receiver. We have used an Nvidia Jetson AGX Xavier, as it was developed for automotive use, has a built-in GPU, and runs full Ubuntu, which makes development easy. The code for synchronizing the camera, GNSS and remote control, is written in C++ and Python and synchronized through the Robot Operating System (ROS).

## 3. Data Collection

**Figure 5:**
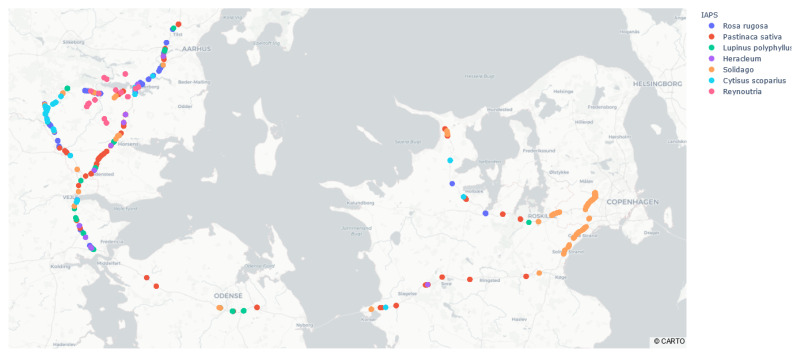
Overview of the collected images and IAPS.

Images were collected along the Danish state roads near the Midwestern part of Jutland, Funen and Zealand during four days between late August and early October 2020 (Figure 5). During image collection, the following seven IAPS were recorded: (1) *Cytisus scoparius*, (2) *Heracleum* (*Heracleum mantegazzianum*, *Heracleum sosnowskyi* and *Heracleum persicum*), (3) *Lupinus polyphyllus*, (4) *Pastinaca sativa*, (5) *Reynoutria* (*Reynoutria japonica* and *Reynoutria sachalinensis*), (6) *Rosa rugosa*, and (7) *Solidago* (*Solidago canadensis* and *Solidago gigantea*).

Data collection was carried out in collaboration with The Danish Road Directorate, which helped selecting road sections of interest as well as identify the IAPS during data collection. An initial data collection campaign was performed in mid to late August near the city of Skanderborg in East Jutland, Denmark. In September, images were collected along a route from Jutland via Funen to Zealand, where *Solidago* was more widespread (Figure 5). In early October, additional data collection was performed near Skanderborg to ensure a sufficient amount of examples of *Reynoutria*.

## 4. Data Annotation

To train and evaluate the detection algorithms, the collected images were annotated according to which IAPS were present in which images. The images contained both invasive plants and background objects (non-invasive plants, road, roadside, sky, traffic signs, etc.); however, many of the collected images contained only background objects. Therefore, the annotations needed to include which IAPS are present in a given image as well as where in the image.

The images were annotated using CVAT (v1.0.0, https://github.com/openvinotoolkit/cvat) (accessed on 10 September 2021) according to the seven IAPS, which were observed during the data collection. Individual IAPS found in an image were annotated by drawing a polygon around it. Depending on the plant density, multiple plants were included in the same polygon or split into different polygons. The annotations were performed by experts at The Danish Road Directorate and took roughly 40–50 person hours. In total, 12263 polygons were annotated in 8387 images out of the 14854 collected images (Table 1). Out of the 8387 images with annotations, 209 images included annotations of multiple IAPS. 6467 images did not contain any IAPS.

### Training, Validation, and Test Sets

When training a deep convolutional neural network, it is important to split the data into a training set, a validation set, and a test set. The training set is used for optimizing the parameters of the network, while the validation set is used for monitoring the performance of the network during training and for comparing the performance of different networks with, for example, different hyperparameters or network structures. The test set acts as a final evaluation of a network. To ensure a proper evaluation on both the validation set and the test set, samples in each set must be independent and have similar class distributions.

The full data set can be split in various ways. The naïve approach is to randomly sample images from the full data set. However, due to the images being captured in close proximity and potentially having overlapping fields of views, information may leak between the data sets when using the naïve approach. Another approach would be to split the data based on image acquisition dates. Unfortunately, the annotated species are not evenly distributed across the acquisition dates (Table 1). This is especially evident for *Solidago* and *Reynoutria*, which were primarily observed on 15 September 2020 and 6 October 2020, respectively. In addition, images may be acquired from the same location across multiple dates, which would make the split data sets non-independent.

Therefore, to ensure independent data sets, the images were clustered based on the locations they were acquired. Images less than 40 m from each other were assigned to the same cluster (With a maximum speed of 130 km/h, and a GPS update rate of 1 Hz, the max distance between adjacent images were 36.1 m). This distance means that images on opposite sides of the motorway could be close enough to be assigned to the same cluster. The images from the same cluster were then restricted to be assigned to the same data sets. To ensure similar class distribution, each cluster was weighted based on the image class distribution within the cluster. The clusters were then randomly shuffled and iteratively added to one of the data sets. Before adding a cluster to one of the data sets, the distribution of the updated set was compared to the expected distribution of that set, using a $\chi^{2}$-goodness-of-fit. The cluster was added to the set, which showed the largest decrease in $\chi^{2}$-value when adding the cluster. If neither of the clusters showed a decrease in $\chi^{2}$-value, the cluster was added to the training set, as it was significantly larger than the validation and test set.

Before splitting the full data set but after clustering, the data set was cleaned. The data cleaning procedure included removing images with *Heracleum*, “Multiple species”, and overlapping annotations. Images with *Heracleum* were removed due to the low number of samples, compared to the other classes. Images with “Multiple species” were removed to avoid ambiguity in the image classification algorithm. Images where two annotations had an intersection over union larger than 75% were removed to avoid ambiguity in the object detection algorithm. In total, 22 images of *Heracleum*, 209 images of “Multiple species” and 19 images with overlapping annotations were removed.

**Figure 6:**
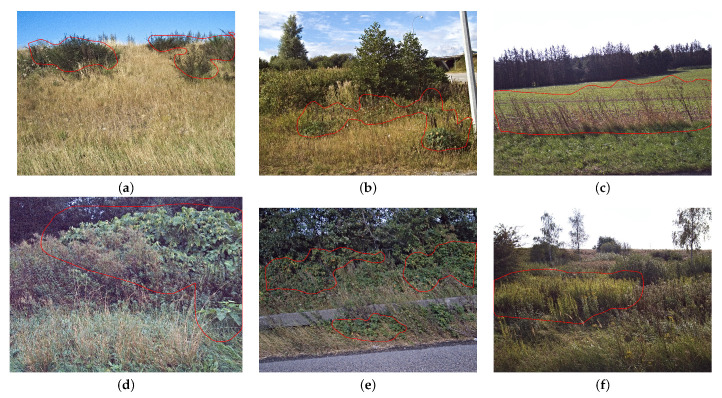
Examples from the training set of (**a**) *Cytisus scoparius*, (**b**) *Lupinus polyphyllus*, (**c**) *Pastinaca sativa*, (**d**) *Reynoutria*, (**e**) *Rosa rugosa*, and (**f**) *Solidago*. The annotations are shown as red polygons.

The full data set was split into a training set, a validation set, and a test set with a 70%/15%/15%-split based on the images in each set (Table 2). Examples of the six IAPS from the training set are shown in Figure 6. Additional examples can be found in the Supplementary Materials.

## 5. Detection Algorithm

Two different methods were explored for finding IAPS in the annotated images: (1) image classification of IAPS and (2) object detection of IAPS. In the former, the task is to correctly classify an image, according to which IAPS are present in the image. In the latter, the task is to localize all IAPS instances in an image and correctly classify the species of each instance. Object detection is therefore a more difficult task than image classification. Both methods are tested as they can provide sufficient information for mapping IAPS, and annotating data for these methods is manageable.

### 5.1. Image Classification of Invasive Alien Plant Species

A preliminary test showed that the processing platform could evaluate images with a resolution of 224 × 224 pixels in real time, using ResNet50-v2 [22]. MobileNet-v2 [23] is a network structure designed with mobile devices in mind. In other words, it aims for good performance with a low computational load. Both network structures are available in Keras [24] that is a high-level API based on Tensorflow [25], and they include weights pre-trained on ImageNet [26], making them easy to evaluate in the case at hand. Therefore, the two network structures ResNet50-v2 and MobileNet-v2 were explored for image classification.

#### 5.1.1. Network Training Procedure

In both network structures, the final classification layer was adapted to match the seven classes (six IAPS and “no species” (Table 2)). The input image size affects the processing time of each image, but can also impact the classification accuracy. Therefore, the network models were trained on five different image sizes: 96 × 128 px, 192 × 256 px, 384 × 512 px, 768 × 1024 px, and 1536 × 2048 px (see Supplementary Materials for a visual comparison of the image sizes). For each image size, a network was trained using either random weights or weights pre-trained on ImageNet and with global max pooling or global average pooling before the final classification layers.

The following three-step data augmentation procedure was applied to the images during training before being processed by the network: (1) randomly flip image horizontally, (2) scale the contrast of each image channel by a random factor between 0.8 and 1.2, and (3) randomly crop the image to a square with side lengths equal to the image height.

All networks were trained on the training set for 100 epochs, using the Adam optimizer [27] with a learning rate of 0.001 and cross-entropy loss. The image batch size varied from 1 to 32 images, depending on the input image resolution with larger batch sizes for smaller image sizes. After each epoch, the network was evaluated on the validation set. The epoch model with the highest accuracy on the validation set during training was saved for further analysis. Before training the networks, the training set was balanced by upsampling the images from the underrepresented classes through repetition to match the number of images of the most represented class, “No species”.

The performance of each of the trained networks was quantified, using the overall accuracy as well as the precision, recall, and F1-score of individual classes. The overall accuracy is the number of images correctly classified out of the total number of images. The precision is the number of correctly classified images of a given class out of the number of predicted images of that class. The recall is the number of correctly classified images of a given class out of the number of annotated/observed images of that class. The F${}_{1}$-score is the harmonic mean of precision and recall.

#### 5.1.2. Results on Image Classification

A total of 40 networks were trained to evaluate the effect of the hyperparameters on the network structure (MobileNetV2 and ResNet50V2). These hyperparameters are input image size (96 × 128 px, 192 × 256 px, 384 × 512 px, 768 × 1024 px, and 1536 × 2048 px), weight initialization (random or pre-trained on ImageNet) and global pooling (average or max).

##### Weight Initialization and Global Pooling

**Figure 7:**
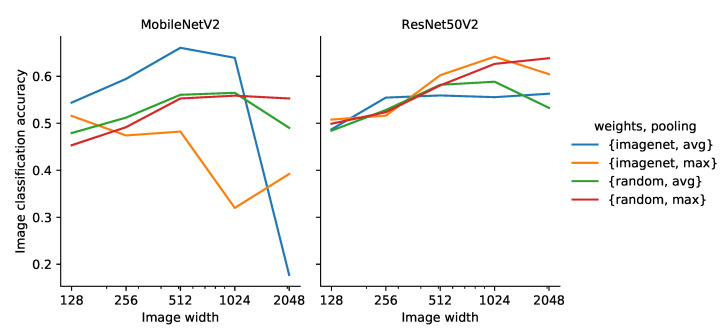
Image classification accuracy across all species on the validation set using MobileNetV2 and ResNet50V2 as a function of input image size.

The two network structures performed similarly on the validation set in terms of classification accuracy, but MobileNetV2 was more sensitive to the weight initialization and global pooling at a given image size (Figure 7). For both network structures, the image classification accuracy generally increased with an increasing image size, except for MobileNetV2, which was initialized with weights pre-trained on ImageNet.

MobileNetV2 initialized with weights pre-trained on ImageNet and average pooling performed better than the other three weight and pooling combinations at all image sizes, except 1536 × 2048 px, where the network failed to distinguish images with no invasive species from images with invasive species. When using randomly initialized weights, there was little difference in accuracy when using average or max pooling. Using the weights pre-trained on ImageNet with max pooling generally resulted in the worst performance. It is not surprising that average pooling performed better than max pooling when using the ImageNet pre-trained weights, as the pre-training was performed using average pooling.

ResNet50V2 showed much less variation between the choice of weights and pooling; however, at larger image sizes (768 × 1024 px and 1536 × 2048 px), the models based on max pooling outperformed the models based on average pooling. The ResNet50V2 models showed no trends in terms of weight initialization.

As a result, MobileNetV2, using weights pre-trained on ImageNet and average pooling, as well as ResNet50V2, using weights pre-trained on ImageNet and max pooling, were selected for further analysis. From this point on, these two configurations will simply be referred to as MobileNetV2 and ResNet50V2, respectively.

##### Species Classification

The average precision, recall and F${}_{1}$-score (data not shown) showed similar trends to the accuracy (Figure 7) with respect to image size for both MobileNetV2 and ResNet50V2. The highest average precision and recall on the validation set was achieved by ResNet50V2 with an input image size of 768 × 1024.

**Figure 8:**
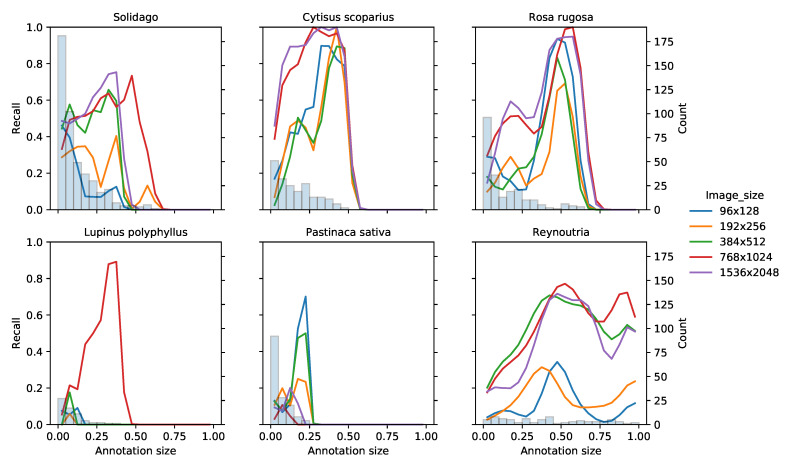
Recall of the ResNet50V2 models on the validation set as a function of the annotation size relative to image size. The validation images were binned according to the relative size of their respective annotations with respect to the image size (“annotation size”). Each line shows the recall (left axis) of a ResNet50V2 model with a given input image size across the binned annotation sizes. The recall lines were “smoothed”, using kernel density estimation. The number of images in each bin is shown as vertical bars (right axis).

Across input image sizes and IAPS, the recall of the ResNet50V2 models varied with the size of the annotations. The recall of each IAPS using ResNet50V2 varied across both input image sizes as well as the combined size of annotation in a given image in the validation set (Figure 8). *Lupinus polyphyllus* and *Pastinaca sativa* both showed very poor recall across image sizes and annotation sizes, due to the relatively few images and small annotation sizes in the images. *Reynoutria* also contained relatively few images, but the size of their annotations ranged from less than 5% of the image to almost the entire image; this range may have contributed to it having a higher recall. For all six IAPS, except *Reynoutria*, the largest image count was in the lowest annotation size. However, the recall of these images was generally much lower than the average recall of a given input image size and IAPS. This indicates that the size of the IAPS in the images was more important than the number of images when training the ResNet50V2 network for classifying IAPS. The highest recall for a given IAPS and input image size was generally achieved at annotation sizes between 25% and 65%. Generally, the two image sizes 768 × 1024 and 1536 × 2048 performed similarly on the individual species and outperformed the other three image sizes, except 384 × 512, which performed comparably to 768 × 1024 and 1536 × 2048 in *Solidago* and *Reynoutria*. This is particularly evident at small annotation sizes in *Cytisus scoparius* and *Rosa rugosa* and at all annotation sizes in *Reynoutria*.

When evaluating ResNet50V2 with an input image size of 768 × 1024 on the test set (Table 3), there was a drop in accuracy (64.2% to 59.3%), average precision (58.5% to 54.4%), average recall (47.2% to 43.3%) and F${}_{1}$-score (49.4% to 45.6%) compared to the validation set. *Reynoutria*, *Rosa rugosa*, *Solidago* and, to some extent, *Cytisus scoparius* performed reasonably well, with both precision above 60% and recall above 50% (except *Cytisus scoparius*). *Lupinus polyphyllus* and *Pastinaca sativa* were the two worst performing classes, both with a precision and recall less than 10%. Disregarding *Lupinus polyphyllus* and *Pastinaca sativa*, the average precision and recall increased to 73.2% and 58.5%, respectively.

##### Invasive vs. Non-Invasive

In some cases, the specific invasive species may not be relevant, e.g., when screening images on the processing platform to decide which images to save and which to discard. In that case, it may be more relevant to distinguish images with IAPS from images without IAPS than to determine the exact IAPS.

To explore this, the IAPS were merged into one class, “invasive species”, after being evaluated by MobileNetV2 and ResNet50V2 networks. This means that classification errors between IAPS were effectively ignored.

**Figure 9:**
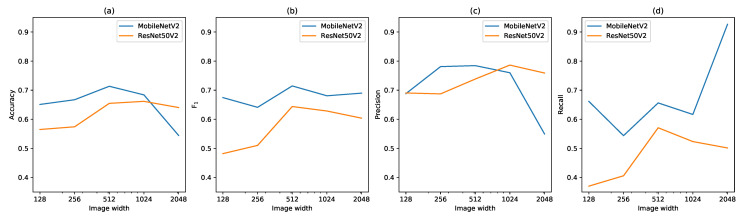
Accuracy (**a**), F${}_{1}$ score (**b**), precision (**c**) and recall (**d**) of IAPS on the validation set for MobileNetV2 and ResNet50V2 as a function of image size. All invasive species are grouped together, and misclassifications between them are ignored.

After merging the IAPS, MobileNetV2 performed better than ResNet50V2 in terms of accuracy, F${}_{1}$-score, precision and recall at most image sizes (Figure 9). This shows that MobileNetV2 was better at distinguishing between images with “invasive species” and “non-invasive species”, while ResNet50V2 was better at distinguishing between the individual IAPS. The highest precision of “invasive species” was achieved by ResNet50V2 with an image size of 768 × 1024 (78.6%), closely followed by MobileNetV2 at image sizes 196 × 256 (78.1%) and 384 × 512 (78.4%). The highest recall was achieved by MobileNetV2 with an image size of 1536 × 2048 (92.6%) but at a cost of a low precision (54.9%), compared to the other networks. This was caused by the network almost exclusively predicting images as one of the invasive species regardless of the input image.

MobileNetV2 with an input image size of 384 × 512 showed the highest F${}_{1}$-score (71.4%) on the validation set and, therefore, best trade-off between precision and recall. Evaluated on the test set, the network achieved an accuracy of 68.3%, precision of 72.3% and recall of 65.9% for invasive species (Table 4). Similar to ResNet50V2 on species classification, the classification errors are mainly caused by *Lupinus polyphyllus* and *Pastinaca sativa*.

##### Cluster Detection

When forming the data sets, the images were clustered based on the location where they were captured. Each cluster represents a group of invasive plants and the area around the group. The purpose of the system is to detect where IAPS occurs and therefore it may be sufficient in an online system if only some IAPS in a cluster are detected. When an IAPS is detected, a sequence of images before and after may be saved for later review — either by a manually inspection or automatically with a slower but more accurate algorithm.

**Figure 10:**
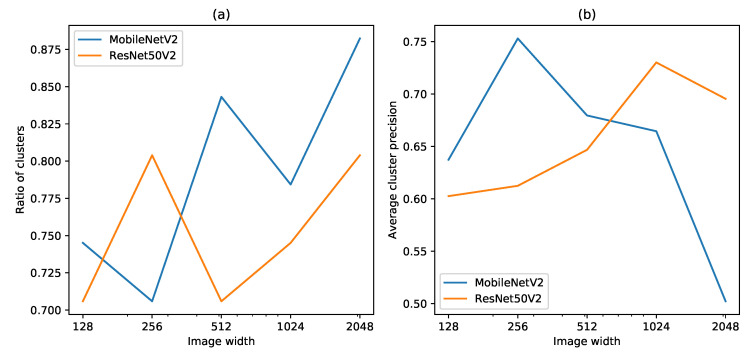
Classification of images in clusters on the validation set. (**a**) Ratio of clusters with at least one image correctly classified as invasive. (**b**) Average cluster precision of IAPS of clusters with at least one image correctly classified as invasive.

At least one image with IAPS was classified correctly in 70–87.5% of the clusters on the validation set (Figure 10). The corresponding precision of these clusters was 50–75%. MobileNetV2 with an image size of 384 × 512 detected at least one IAPS in 84.3% of the clusters with an average cluster precision of 68.0% on the validation set.

### 5.2. Object Detection of Invasive Alien Plant Species

You only look once (YOLO) by Redmon et al. [28] is a state-of-the-art, real-time object detection algorithm based on deep learning. Redmon and Farhadi [29] made YOLOv3 that is able to detect objects in an image with high accuracy at high speed. The YOLOv3 model predicts objects with a bounding box around the detected object and a confidence score (percentage) of the predicted class. With an image size of 608 × 608 pixels, it archives a mean average precision (mAP) of 57.9 mAP on the COCO dataset at a frame rate of up to 20 FPS. The COCO dataset contains images of 80 object categories with labeled data for training, validation, and test. The YOLOv3 code is written in pure C-code optimized for GPU processing, which makes it able to run in real time on the Jetson AGX Xavier platform.

A YOLOv3 model was trained, using the annotations from the training and validation dataset as shown in Table 2. The model was configured and trained with an image size of 832 × 832 pixels for the six classes of plant species. The average training loss was used to determine when to stop the training. Redmon and Farhadi recommends at least 2000 iterations for each number of classes, but 22,000 iterations were chosen, as the training loss was stabilized after this number of iterations. The trained YOLOv3 model was tested using the images from the test data set scaled to a pixel size of 608 × 608.

#### Results on Object Detection

The confusion matrix in Table 5 shows the number of predicted species in comparison to the observed and annotated plant species in the test dataset. The precision, recall, and F1-score are shown in Table 6 for objects detected with a YOLOv3 confidence threshold above 25% based on the confusion matrix. The result shows that the trained YOLOv3 model is able to find, on average, 26% (recall) of the IAPS. The species *Cytisus scoparius*, *Reynoutria* and *Rosa rugosa* have a recall of 34% to 45%, while the recall of *Lupinus polyphyllus* and *Pastinaca sativa* are detected less often and scores a recall of 5% and 13%, respectively. A manual inspection of labels and predictions showed that a big part of the undetected plants are either withered or too small to be detected. However, the recall on the training and validation is 92% indicating that there are differences between the appearance of plants in the different data sets. This difference is likely caused by the spatial distance between locations where the train, validation, and test sets were collected. The average precision is 77% for the detected plant species, where the most difficult species to detect is *Pastinaca sativa* with a precision of 47%. The result also shows that the trained ResNet50V2 network model outperforms YOLOv3 for *Rosa rugosa*, while YOLOv3 is best at detecting the more difficult species *Lupinus polyphyllus* and *Pastinaca sativa*.

Initial results with YOLOv3 on a training dataset from driving in August 2020 verified on a test dataset from driving in September for five different species gave an average recall of 10% and an average precision of only 37%. This indicates that the trained models are sensitive to changes in the appearance of the plants during the season as they grow, flower and wither.

**Figure 11:**
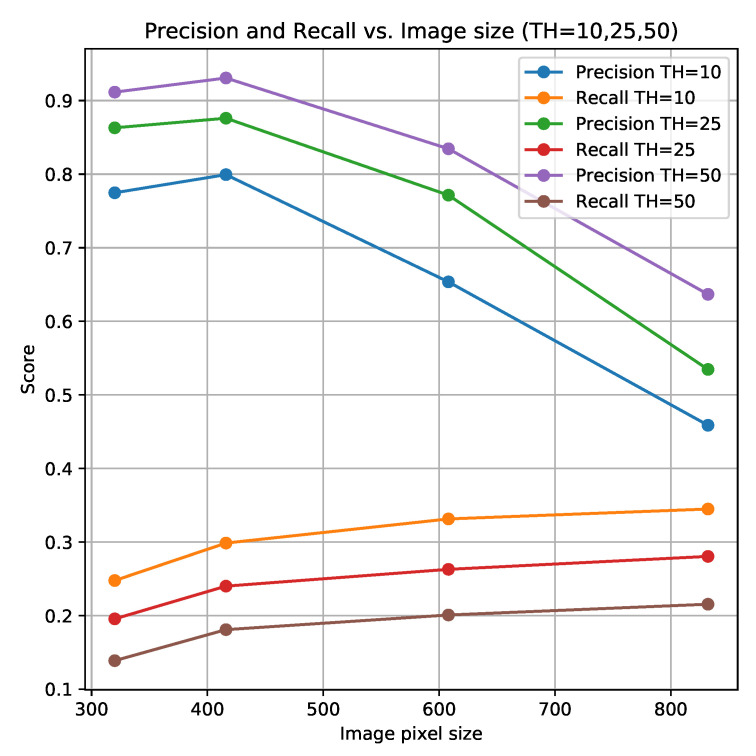
Average precision and recall as function of image size used by YOLOv3 to predict objects of six different plant species with a confidence threshold (TH) of 10%, 25% and 50% (see Supplementary Materials for a visual comparison of the image sizes).

Figure 11 shows the impact of the scaled image size and confidence threshold (TH) on the precision and recall. The average precision (88% vs. 77%) is higher with a scaled image size of 416 × 416, compared to 608 × 608 pixels with a confidence threshold of 25%. The recall is only 2%-points lower (24% vs. 26%) with the scaled image size of 416 × 416.

The speed performance of the trained YOLOv3 model was measured for the model inference on the Jetson AGX Xavier platform and a Ubuntu computer equipped with an Nvidia GTX1080 GPU card. Table 7 shows the maximum frame rate at which it would be possible to process and detect IAPS with the YOLOv3 model. The maximum frame rate was based only on inference time on the GPU and does not include time to load and resize images. In a scenario where IAPS are detected real-time when driving, the images are stored uncompressed in the computer’s RAM and the read time is almost eliminated. Scaling images takes place on the CPU faster than the GPU can process the previous image and does not contribute significantly to the execution time. We, therefore, estimate that the real-time frame rates listed would be achievable on the Nvidia Jetson AGX Xavier platform.

With images scaled to 416 × 416 pixels, it would be possible to process the images with a rate of six images per second. The model with this image size had an average precision of 80% and detected 30% of the IAPS with a confidence threshold of 10%. This precision and recall (marked with bold in Table 7) are higher than the scores achieved when using an image size of 608 × 608 with a confidence threshold of 25% as presented in Table 6. However, the recall was lower, and for *Pastinaca sativa* and *Lupinus polyphyllus*, only 3% and 10% of the plants were found, respectively.

Based on the presented performance results, a real-time system performing object detection at driving speeds of 110 km/h would be possible to realize achieving a high precision of 80% in detection of IAPS.

## 6. Mapping

Based on the collected image data in August and September, the predictions based on object detection (YOLOv3) were analyzed and the GPS locations were plotted on the map of Denmark to illustrate the spatial distribution of detections.

**Figure 12:**
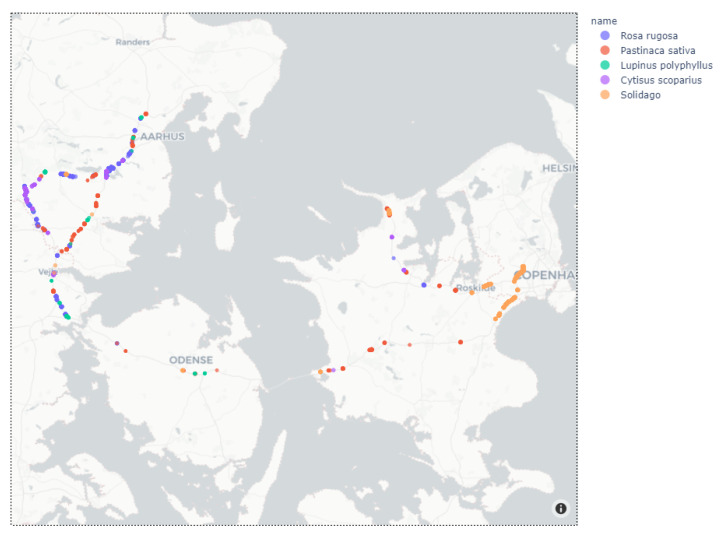
Location of invasive plants found and marked on map of Denmark based on collected and analyzed images in August and September. (© OpenStreetMap contributors).

**Figure 13:**
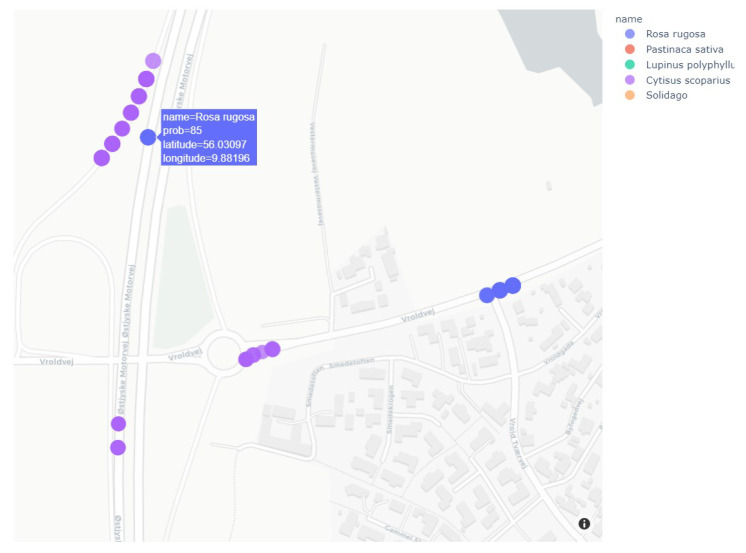
Zoom of location of invasive plants with information on map with a resolution of 1 s. between markers. (© OpenStreetMap contributors).

Figure 12 shows a section of Denmark, where markers are colored based on the predicted name of plant species. Such a map can be created based on a selection of plant species or a selected range of driving dates to highlight temporal patterns in detections. Figure 13 shows an enlarged section of the map, which gives more details about the location of the plants along the roads. For more examples of different maps, see Supplementary Material. The resolution between GPS updates was 1 s. In the case of a driving speed of 100 km/h, the resolution between markers will be 27.8 m. Therefore, each marker represents more images and predictions since the camera frame rate is higher than the GPS update rate.

Although not all IAPS were found, we still see the same trends in the map of detections in Figure 12 and the map with annotated IAPS in Figure 5. As an example, *Solidago* was found in the area of Copenhagen and *Rosa rugosa* was found on the motorways south of Aarhus. These similarities indicate that automated detection of IAPS in geotagged images can give an accurate overview of the IAPS’ locations.

## 7. Discussion

Recent developments in machine learning have made it possible to use automated camera-based solutions for plant ecological monitoring [30,31]. A prerequisite for being able to do automatic recognition of images is that the images have sufficient quality. In this work, we have tested whether the prerequisites are present to use machine learning for the automatic recognition of invasive plants along Danish motorways.

Four cameras were evaluated, of which the two machine-vision cameras provided sufficient image quality when shooting perpendicular to the direction of travel. The selected camera allowed for short exposure times so that images could be captured with minimal motion blur from a car driving at high speeds (>110 km/h) on the motorway. As the maximum driving speed is the limiting factor of the required exposure time, the camera system can, therefore, be transferred to smaller roads with slower driving speeds. On roads other than the motorway, however, the distance from the camera to the plant will often be shorter, as there is rarely an emergency lane between the road and the roadside. This means that the plants will cover a larger part of the image if the current optics are retained. The classification results showed that while the majority of the plants were small (<25% of the image), the medium-sized plants (25–65% of the image) were the easiest to recognize (Figure 8). Assuming that plants are closer to the camera on smaller roads, they will also cover a larger part of the image, and thus the small plants will likely be easier to recognize.

Plant species vary in appearance during their growth stages and are typically easiest to identify when they flower. A machine learning model trained on images of a plant species in one growth stage may not perform well when confronted with images of the species in another growth stage. The performance of our detection model would likely improve considerably if training and test data were restricted to images of the plant species in bloom. However, from a management point of view, it may be desirable to identify the IAPS as early as possible, e.g., before flowering, to combat the IAPS before they spread further.

However, it is not possible to make only a single inspection since the plants are not flowering at the same time. Therefore, it will be necessary to collect photos at different times of the season. In this study, we have only collected photos during August to October. This has shown that plants can be recognized, but that the detection rate decreases when tests and training images are taken while the plants are in flowering in August and after flowering in October, respectively. For example *Lupinus polyphyllus* is very characteristic with its colored flowers, but more discrete after flowering. We, therefore, believe that the recognition results obtained can be improved if the plants are in bloom both in the images used for training the system and the images collected during monitoring.

Therefore, images should be collected for an entire season before the system will able to provide support unless images are annotated immediately after a collection and used for training the system. Still, ongoing supervision will be necessary to minimize false negatives, i.e., IAPS that are overlooked. These will typically be plants that grow under conditions not previously seen in the training material.

There are various sources for street photos besides the camera used in this study, for example, Cowi Gadefotos, Mapillary, and Google Street View. However, in order to increase the probability of the plants being detected, the images should be taken at the right time in terms of development and flowering, where they are most distinct. The recording must, therefore, be synchronized with the development of the plants, which cannot be ensured from existing street photos sources. Therefore, we believe that a dedicated camera system for detecting IAPS is advantageous.

We have used two different paradigms in image recognition: per-image classification (ResNet50v2 and MobileNetv2) and bounding-box detection (YOLOv3). In per-image classification, we assign a plant species to the entire image but do not consider where the plant is located in the image. When conducting bounding-box detection, we detect both the plant species and where this plant is located in the image. Although the level of detail at YOLOv3 is greatest, it also requires more time to annotate training data for it. This is on top of the fact that only a small part of the collected images from 2020 have more than one invasive species in the same image and pointing out the location in an image that covers few meters of the roadside, is not needed when controlling the plants. In terms of detection rate, this study has found no clear winner between ResNet50v2 and YOLOv3.

Plants often grow in clusters. This means that the consequences of overlooking IAPS in an image are limited if the IAPS are detected in neighboring images. Furthermore, the detection in neighboring images can be used to adjust the confidence in the detection of plants if the same species are predicted with a high probability in the neighboring images.

ResNet50v2 and MobileNetv2 are more resource efficient and will be able to run in real-time on the current processing platform without further optimization. YOLOv3 can run in real time with scaled-down images on the processing platform if the entire roadside is to be scanned. If the algorithm is to run in real time on the computer, one can avoid having to save the images for later processing. However, saving images is necessary to improve the training material, and to assess the performance.

Previous studies have focused on the recognition of invasive plants based on their spectral reflections. This includes He et al. [32] and Lass et al. [33], who used hyperspectral remote sensing to detect invasive plant species. Bradley [8] noted that a disadvantage of hyperspectral aerial photography is the price, which speaks against using hyperspectral cameras. Since 2011, when He et al. [32] published their study, hyperspectral images from satellites have become cheaper with a higher resolution. It is, therefore, likely that hyperspectral images taken from satellites may become a competitive alternative to aerial recordings. However, a disadvantage of hyperspectral photography is that the images must be calibrated to local conditions at each recording since the nutrition of the plants has a great influence on their hyperspectral reflectance. This argues for including textural information that is more robust to differences in nutrition.

Bradley [8] argued that the spatial resolution is important for recognizing plants in aerial photographs based on textural features. Bradley [8] states that the limit is 1 px per m${}^{2}$, which is what was available from the aerial photos. This makes it possible to detect large plants and trees. However, we would argue that accurate recognition with textural features requires significantly more than 1 px per plant unless plants are in distinctive clusters. Therefore, a significantly higher resolution is also required. Since Bradley [8] published her work in 2014, however, the achievable resolution has become significantly lower, which is largely due to the availability of affordable UAVs with high-resolution cameras, which can bring the resolution above 1 px per cm${}^{2}$. Moreover, convolutional neural networks only make sense if the plants have a spatial extent in the image. As a starting point, we argue that the resolution must be sufficient for a trained person to be able to recognize the object. This is required to annotate the training data and ensure that the image contains sufficient information for the plants to be identified.

The roadsides are some of the longest continuous areas in Denmark, where nature is allowed to remain relatively undisturbed, except for the vegetation close to the road, which is mowed to increase roadway safety. This makes the roadsides interesting for the detection of invasive plants, as the plants can spread over long distances [6,7]. However, it is important to keep in mind that since we only scan the roadside, there is a significant geographical bias, which was also noted by Wolmarans et al. [34]. The results can, therefore, only be used to monitor invasive plants on the roadside and not to determine the spread of invasive plants away from the roads. Since our method has been developed for roadside monitoring, our approach will not directly applicable for scanning large flat areas, as is possible with aerial photos. In return, our solution can scan plants from a side view, where plants are often easier to recognize than from above since that is the same viewpoint from which we usually see plants. Furthermore, we assess that our method is robust to moderate plant stress as opposed to methods that are based solely on spectral analysis. Although our method was developed for mounting on cars, with slight modification, it will be possible to mount the equipment on a low-flying drone, whereby large areas can be scanned. This will provide significantly higher resolution than normal aerial photos and satellite images for smaller plants to be detectable. On the other hand, the capacity will be significantly lower compared to traditional aerial and satellite images. When scanning along roads, we assess that our equipment has sufficient capacity for scanning roadsides, as it only requires mounting onto the roof of vehicles that are already driving on the road.

## 8. Conclusions

This article has demonstrated a camera assisted roadside monitoring system that makes it possible, at high speed, to record images of invasive alien plant species along the Danish state roads. It was subsequently possible to identify the plant species in the images with reasonable precision and recall using deep learning. The developed data collection platform consisting of a machine-vision camera, global positioning system, a remote control and a processing platform enabled high resolution image acquisition at speeds up to 130 km/h on the Danish motorways. Multiple off-the-shelf deep learning models were trained and evaluated for detecting six invasive alien plant species (*Cytisus scoparius*, *Lupinus polyphyllus*, *Pastinaca sativa*, *Reynoutria*, *Rosa rugosa*, and *Solidago*) in the collected images. The models showed reasonable precision and recall in recognizing the invasive alien plant species. However, there is still room for improvement, particularly with respect to *Lupinus polyphyllus* and *Pastinaca sativa*, which were difficult to detect, probably due to the lack of images of flowering individuals. Future work may also include additional invasive alien plant species, such as *Heracleum*, which was excluded from this work, due to a lack of data material, or plant species considered invasive alien plant species in other countries than Denmark. Further improvements in precision and recall may be achieved by collecting images while the plants are in flowering.

Based on the results presented in this article, we believe that a real-time system with full coverage at driving speeds of 110 km/h will be possible in the near future to map invasive alien plants automatically.

## Figures and Tables

**Table 1:** Overview of collected and annotated images. *C* and *I* are the number of clusters and number of images each species appear in on a given date, respectively. *P* is the number of annotated polygons of a each species on a given date. A cluster consists of multiple images acquired from the same location—potentially across multiple dates. An image contains zero, one, or multiple annotations.

Invasive Plant	2020-08-17			2020-08-24			2020-09-15			2020-10-06			Total		
Species	*C*	*I*	*P*	*C*	*I*	*P*	*C*	*I*	*P*	*C*	*I*	*P*	*C*	*I*	*P*
*Cytisus scoparius*	2	57	116	21	1011	1396	4	74	106	0	0	0	26	1142	1618
*Heracleum*	2	2	2	13	16	23	3	3	3	1	1	1	19	22	29
*Lupinus polyphyllus*	4	47	100	16	357	821	10	161	229	0	0	0	30	565	1150
*Pastinaca sativa*	9	114	230	41	403	691	28	424	532	0	0	0	77	941	1453
*Reynoutria*	0	0	0	1	26	26	0	0	0	15	683	779	16	709	805
*Rosa rugosa*	15	279	460	43	1267	1684	4	96	122	0	0	0	60	1642	2266
*Solidago*	1	7	8	12	110	163	50	3012	4152	1	28	28	64	3157	4341
Multiple species	5	39	157	17	125	334	9	44	108	1	1	2	32	209	601
No species	24	580	-	114	2396	-	87	2379	-	25	1112	-	243	6467	-

**Table 2:** Overview of training, validation, and test set. *C*, *I*, and *P* are the number of clusters, images, and polygons of each species in the respective data sets. A cluster consists of multiple images acquired from the same location—potentially across multiple dates. An image contains zero, one, or multiple annotations.

Species	Training			Validation			Test		
	*C*	*I*	*P*	*C*	*I*	*P*	*C*	*I*	*P*
*Cytisus scoparius*	11	780	1109	6	199	279	9	161	226
*Lupinus polyphyllus*	20	426	851	4	65	103	6	66	127
*Pastinaca sativa*	47	665	1005	18	152	252	12	124	196
*Reynoutria*	6	511	578	5	77	93	5	124	128
*Rosa rugosa*	35	1189	1639	10	226	327	15	225	287
*Solidago*	38	2239	2792	8	462	791	18	451	744
No species	145	4493	-	49	979	-	49	995	-
Total	151	10,303	7974	51	2160	1845	54	2140	1708

**Table 3:** Confusion matrix of the ResNet50V2 (ImageNet weights, max pooling, and 768 × 1024 px input images) model predictions on the test set.

		Predicted							
		*Cyt.*	*Lup.*	*Pas.*	Rey.	*Ros.*	Sol.	“No s.”	Recall
**Observed**	*Cytisus scoparius*	54	2	0	0	0	2	103	0.335
	*Lupinus polyphyllus*	0	2	4	0	0	4	56	0.030
	*Pastinaca sativa*	0	1	5	0	0	11	107	0.040
	*Reynoutria*	0	0	0	91	0	1	26	0.771
	*Rosa rugosa*	2	4	6	0	115	13	85	0.511
	*Solidago*	0	4	2	0	0	248	197	0.550
	“No species”	11	9	73	51	2	95	754	0.758
	Precision	0.806	0.091	0.056	0.641	0.983	0.663	0.568	

**Table 4:** Confusion matrix of the MobileNetV2 (ImageNet weights, average pooling, and 384 × 512 px input images) model predictions of invasive (Inv.) vs. non-invasive (Non.) IAPS on the test set.

		Predicted		
		Invasive	Non-invasive.	Recall
**Observed**	Invasive	755	390	0.659
	Non-invasive	289	706	0.710
	Precision	0.723	0.644	

**Table 5:** Confusion matrix of the YOLOv3 model predictions on the test dataset with a confidence threshold of 25%. The matrix shows the number of predicted species compared to the observed annotated species.

					Predicted			
		*Cyt.*	*Lup.*	*Pas.*	*Reynoutria*	*Ros.*	*Solidago*	“No s.”
**Observed**	*Cytisus scoparius*	76	0	0	0	0	0	150
	*Lupinus polyphyllus*	0	6	0	0	0	0	121
	*Pastinaca sativa*	0	0	25	0	0	1	170
	*Reynoutria*	0	0	0	56	3	0	69
	*Rosa rugosa*	0	0	0	0	130	0	157
	*Solidago*	4	0	0	0	0	156	584
	“No species”	13	2	28	14	19	57	0

**Table 6:** Precision, recall and F1-score for the YOLOv3 model predicted on the test data set with a confidence threshold of 25% based on predictions in Table 5. ResNet50V2 precision and recall in parenthesis to compare the performance of the two models.

Species	Precision	Recall	F1-Score
*Cytisus scoparius*	0.854 (0.806)	0.336 (0.335)	0.483
*Lupinus polyphyllus*	0.750 (0.091)	0.047 (0.030)	0.089
*Pastinaca sativa*	0.472 (0.056)	0.128 (0.040)	0.201
*Reynoutria*	0.800 (0.641)	0.438 (0.771)	0.566
*Rosa rugosa*	0.872 (0.983)	0.453 (0.511)	0.569
*Solidago*	0.732 (0.663)	0.210 (0.550)	0.326
**Average**	**0.771 (0.535)**	**0.263 (0.380)**	**0.392**

**Table 7:** Frame rate (FPS) vs. precision and recall with a confidence threshold of 10%, 25% and 50% of the trained YOLOv3 model running on the Jetson AGX Xavier platform or a Ubuntu computer equipped with an Nvidia GTX1080 GPU.

Image Size	Xavier	GTX1080	Pre.	Recall	Pre.	Recall	Pre.	Recall
(pixels)	(FPS)	(FPS)	(50%)	(50%)	(25%)	(25%)	(10%)	(10%)
832 × 832	n/a	n/a	0.637	0.215	0.535	0.280	0.459	0.345
608 × 608	3.3	16.1	0.835	0.201	0.771	0.263	0.654	0.331
416 × 416	6.3	27.0	0.931	0.181	0.876	0.240	0.799	0.299
320 × 320	10.0	35.7	0.912	0.139	0.863	0.319	0.775	0.248

## Supplementary Materials

The following are available at https://www.mdpi.com/article/10.3390/s21186126/s1, Figures S1–S6: Examples of IAPS from training data, Figure S1: *Cytisus scoparius*. Figure S2: *Lupinus polyphyllus*. Figure S3: *Pastinaca sativa*. Figure S4: *Reynoutria*. Figure S5: *Rosa rugosa*. Figure S6: *Solidago*. Figures S7 and S8: Visual and relative comparison of image sizes, Figure S7: Image classification. Figure S8: Object detection. Figures S9 and S10: Map examples, Figure S9: IAPS by date. Figure S10: Detection confidence by IAPS.
