# Supplementary figures and images for: Camera Assisted Roadside Monitoring for Invasive Alien Plant Species Using Deep Learning
Source: Sensors (Basel). 2021 Sep 13;21(18):6126. doi: 10.3390/s21186126 (PMC8473160; doi:10.3390/s21186126)

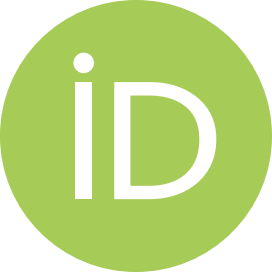

Supplement: Supplementary file 1 [file sensors-21-06126-s001.zip › Definitions/logo-orcid-eps-converted-to.pdf]

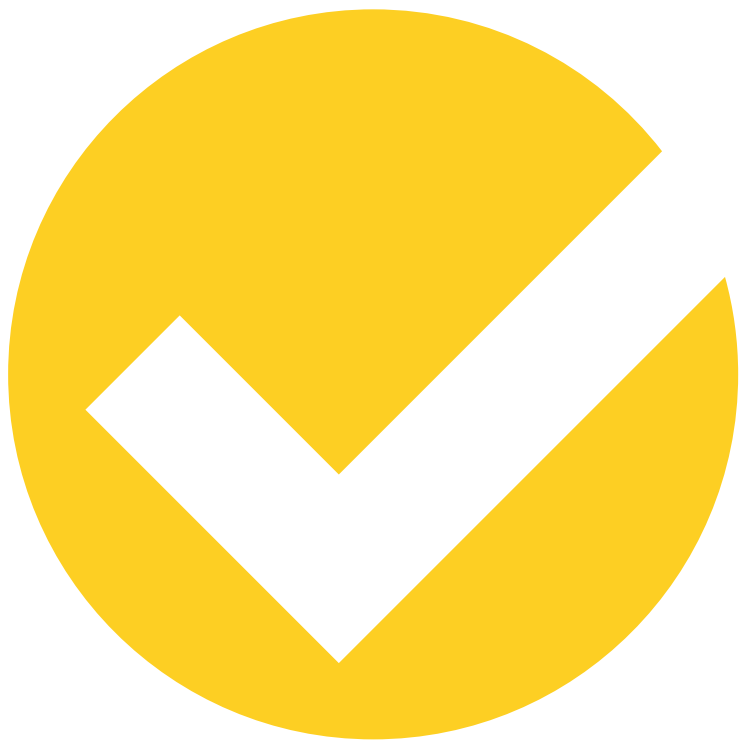

check for  
updates

Supplement: Supplementary file 1 [file sensors-21-06126-s001.zip › Definitions/logo-updates.pdf]

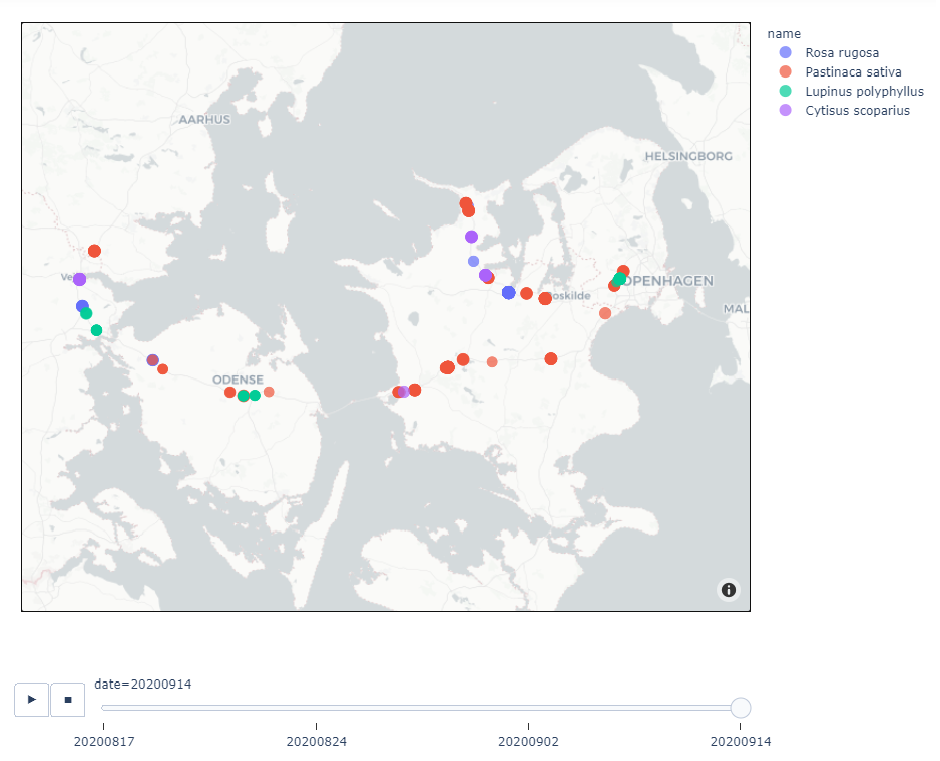

Supplement: Supplementary file 1 [file sensors-21-06126-s001.zip › images/MapDates.png]

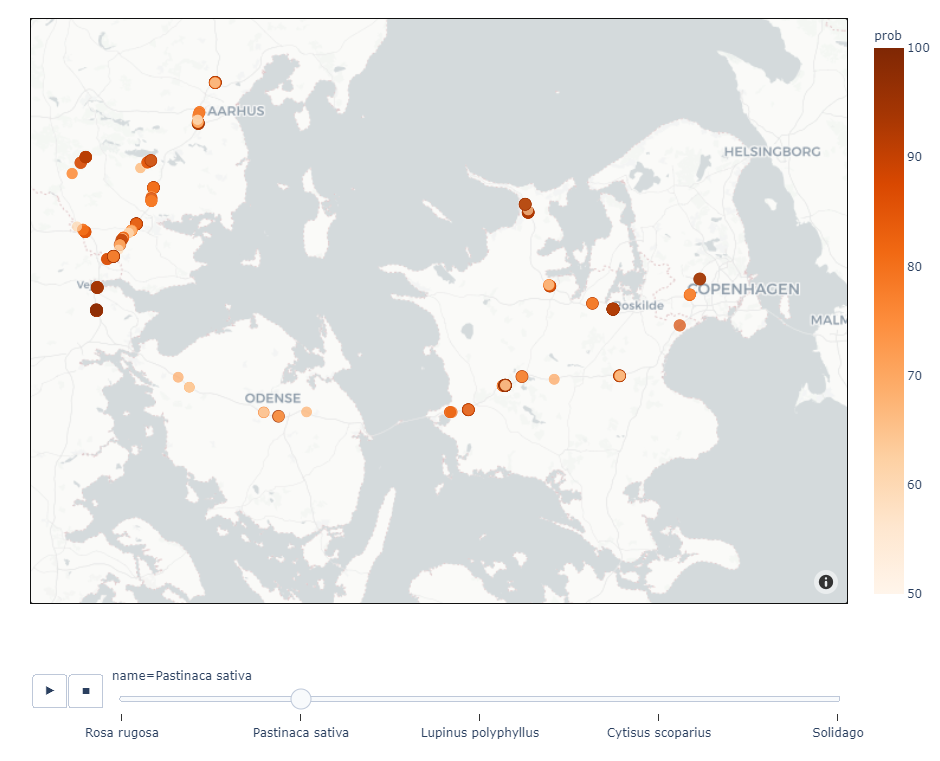

Supplement: Supplementary file 1 [file sensors-21-06126-s001.zip › images/MapPlants.png]

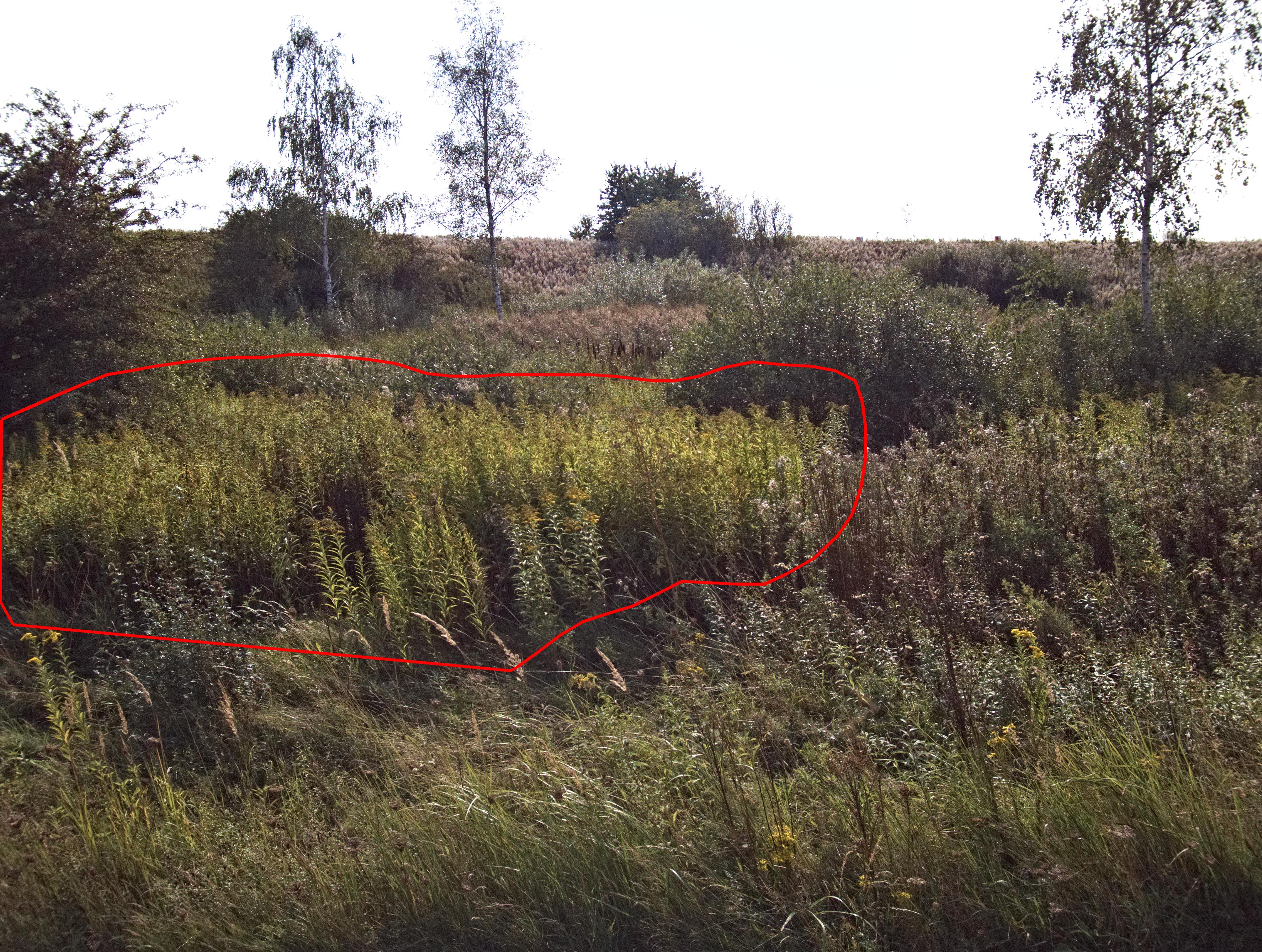

Supplement: Supplementary file 1 [file sensors-21-06126-s001.zip › images/class_examples/Gyldenris_1567_0.1804135225775534_GT_2020-09-15T10_19_40.000Z_CT_1597342172.0005956_12.365552167_55.703139333.jpg]

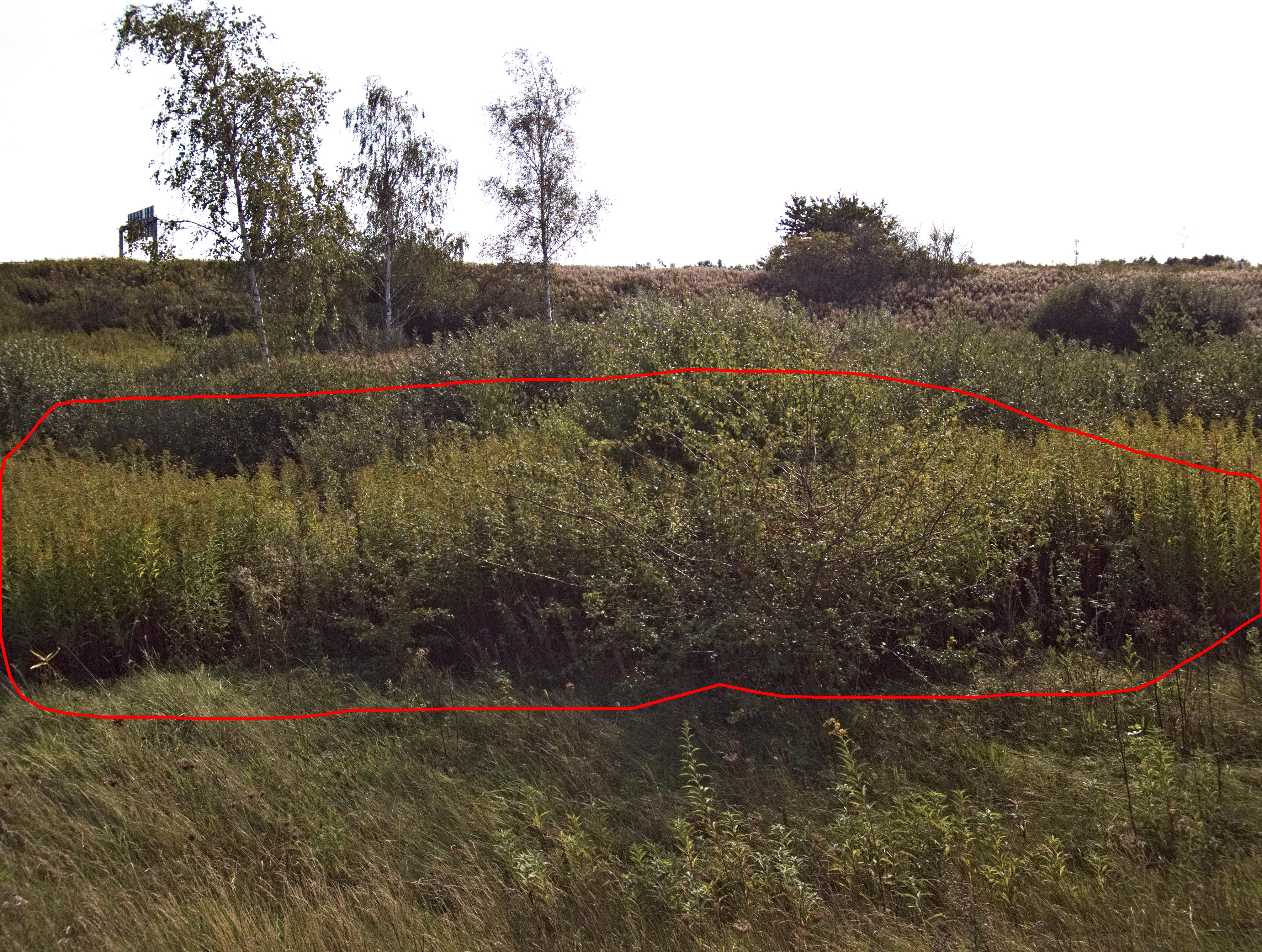

Supplement: Supplementary file 1 [file sensors-21-06126-s001.zip › images/class_examples/Gyldenris_2127_0.31183552265761716_GT_2020-09-15T10_19_38.000Z_CT_1597342170.4056304_12.365277_55.703029333.jpg]

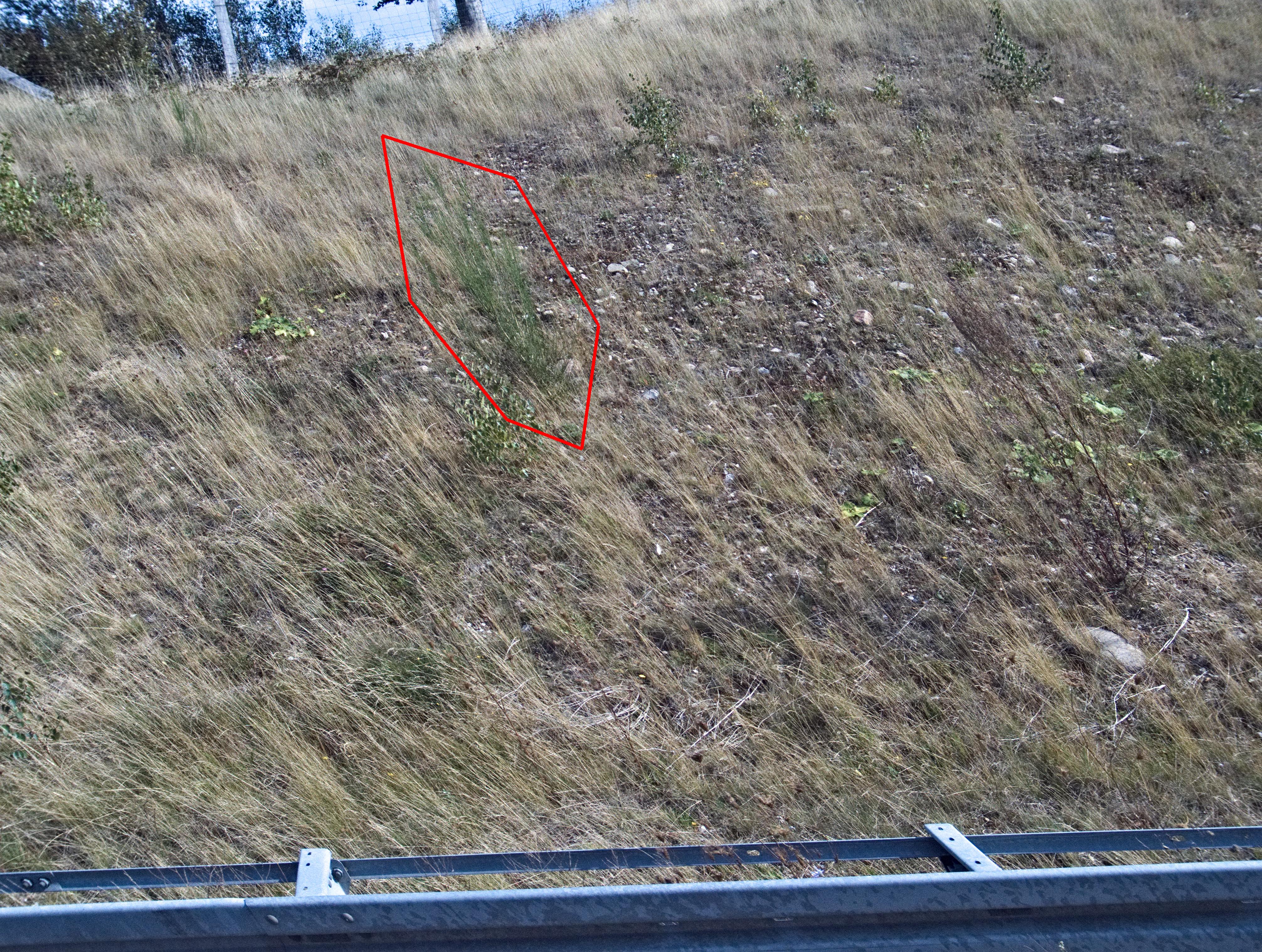

Supplement: Supplementary file 1 [file sensors-21-06126-s001.zip › images/class_examples/Gyvel_78_0.03147259034536807_GT_2020-09-15T11_32_29.000Z_CT_1597346540.3646157_11.583542667_55.8118445.jpg]

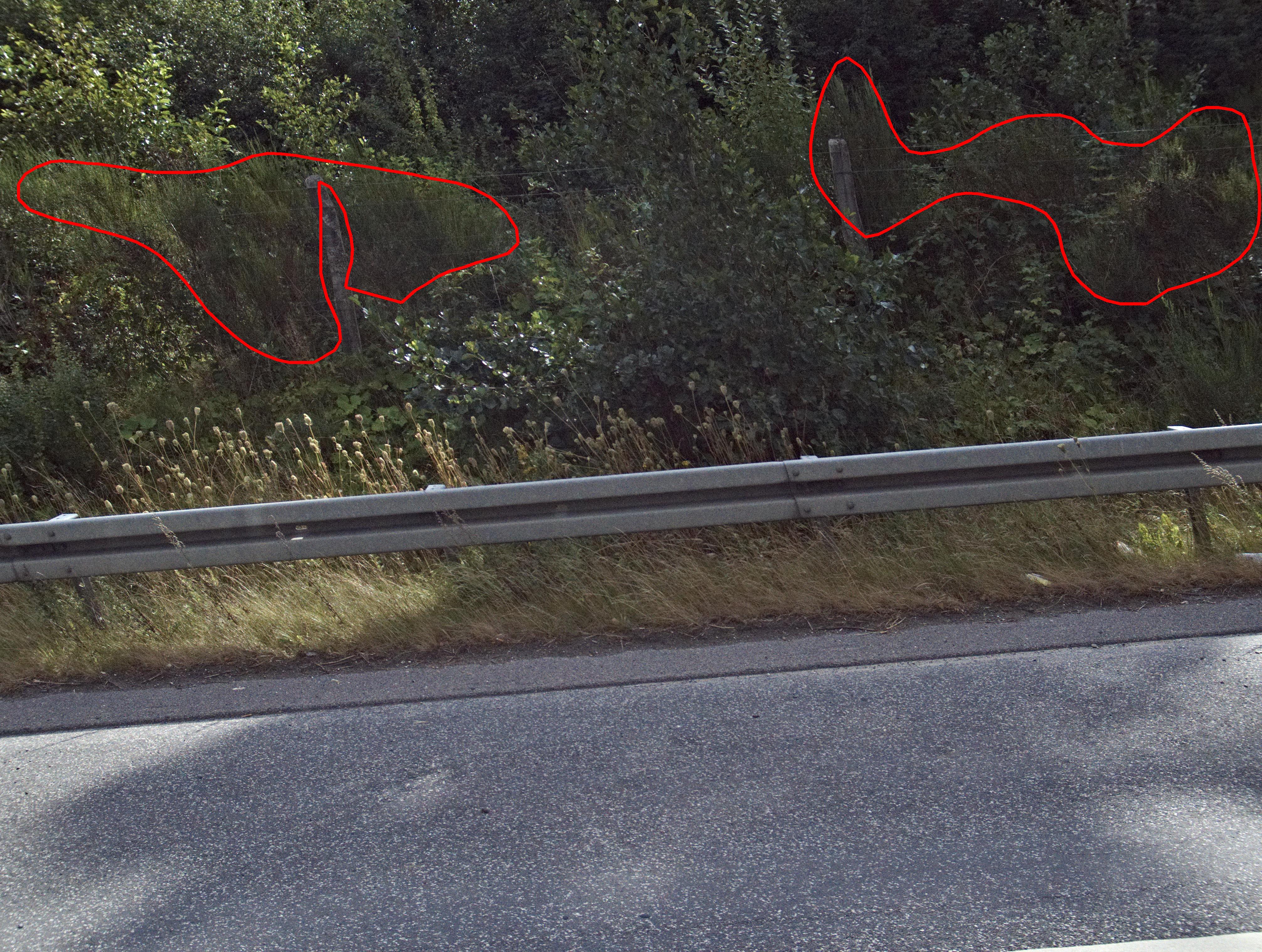

Supplement: Supplementary file 1 [file sensors-21-06126-s001.zip › images/class_examples/Gyvel_273_0.08826127049353832_GT_2020-08-24T08_39_21.000Z_CT_1597332799.5786881_9.569261_55.690328.jpg]

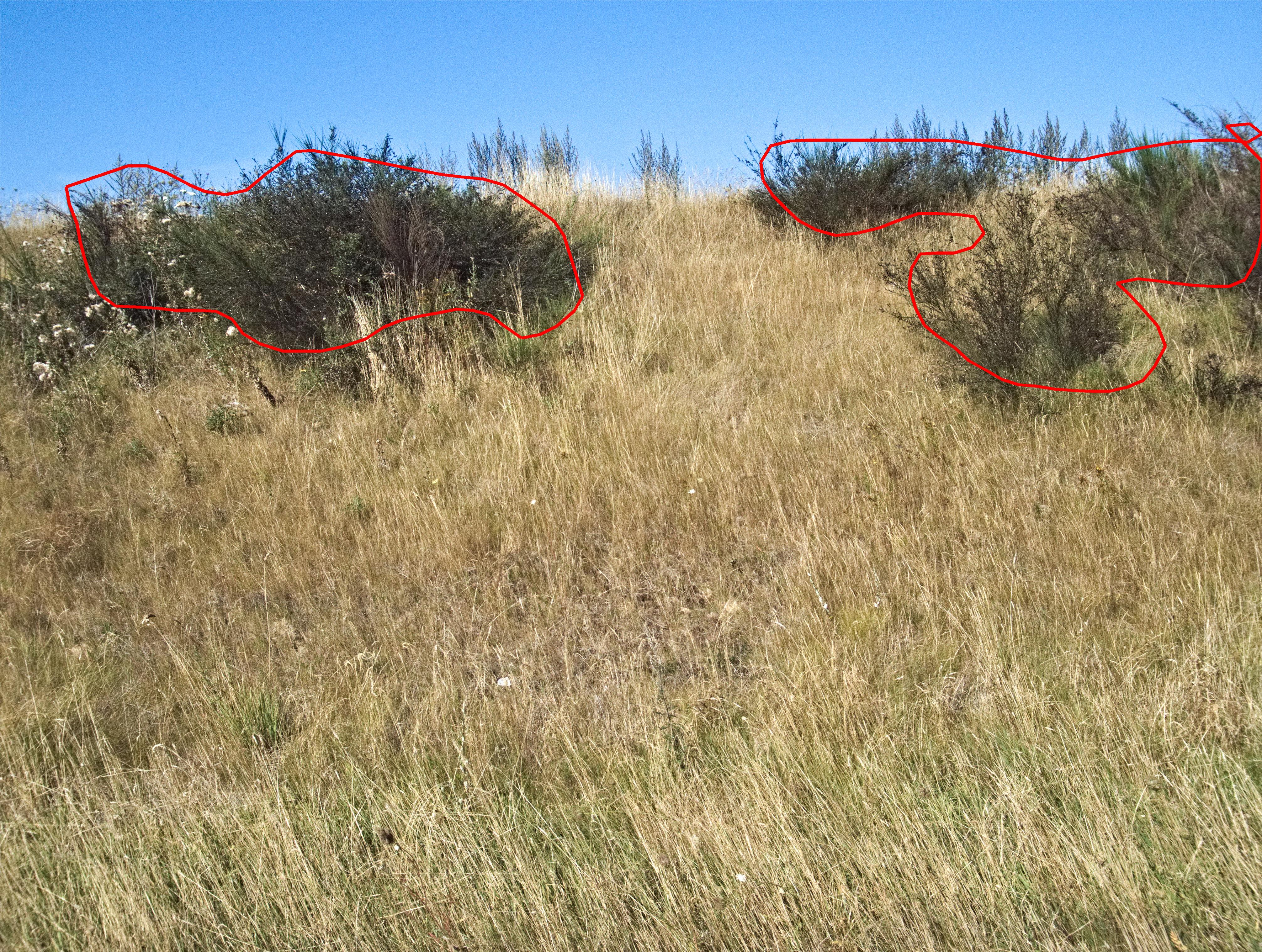

Supplement: Supplementary file 1 [file sensors-21-06126-s001.zip › images/class_examples/Gyvel_351_0.12371339751782945_GT_2020-08-17T09_13_56.000Z_CT_1597331304.2622468_9.881673833_56.031195.jpg]

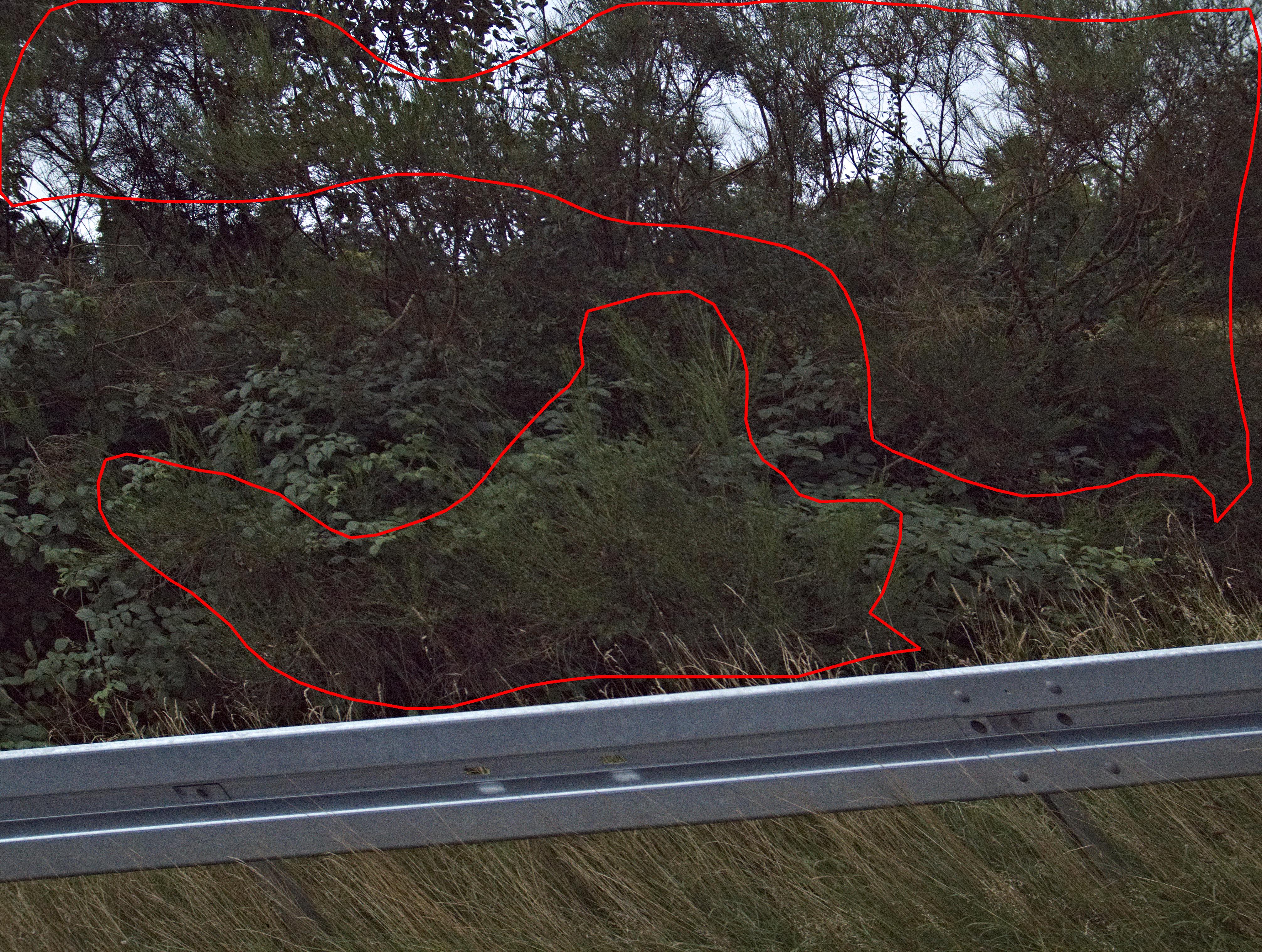

Supplement: Supplementary file 1 [file sensors-21-06126-s001.zip › images/class_examples/Gyvel_663_0.4220091852317638_GT_2020-08-24T09_09_14.000Z_CT_1597334591.7296653_9.395780167_55.975669833.jpg]

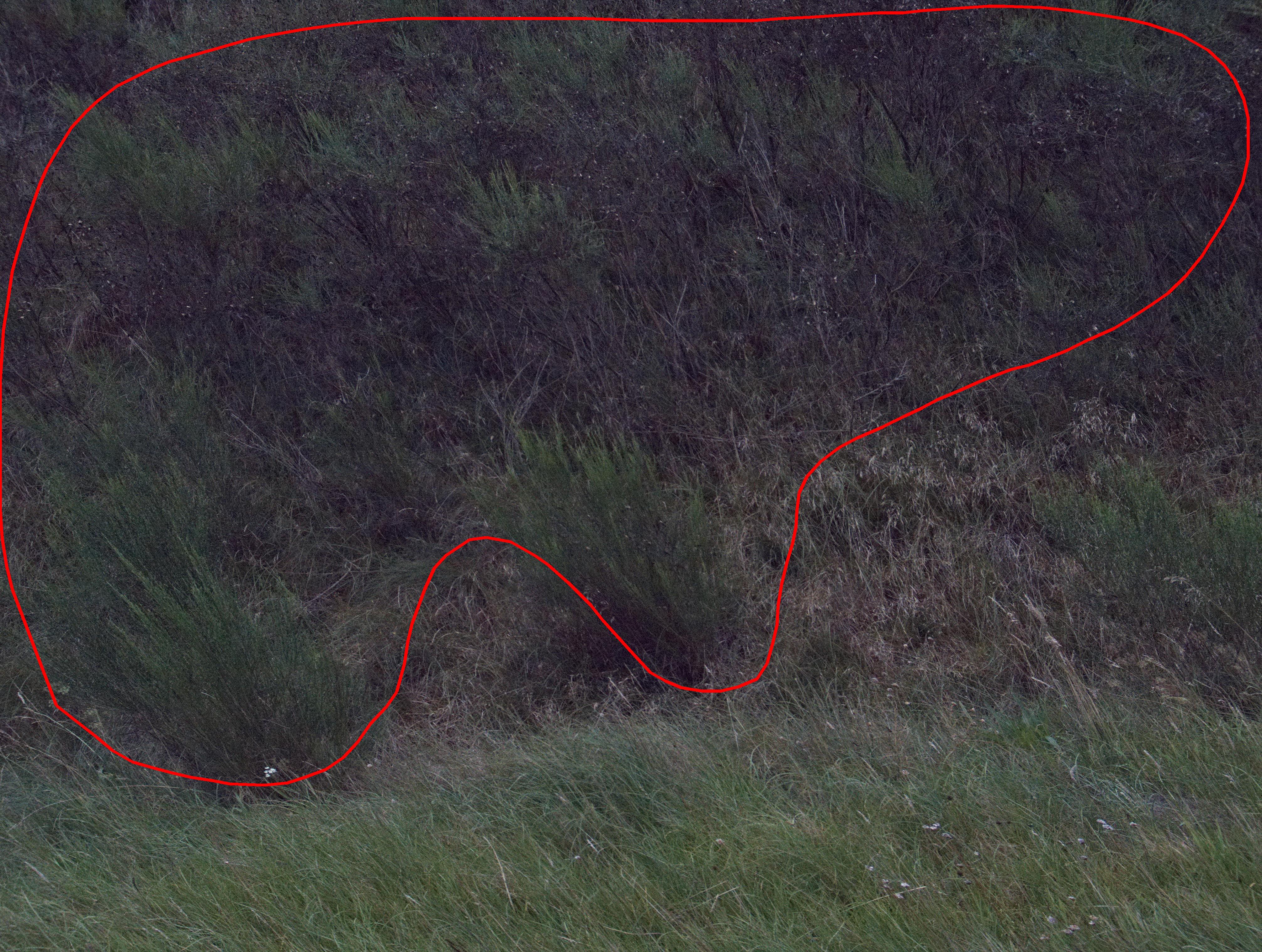

Supplement: Supplementary file 1 [file sensors-21-06126-s001.zip › images/class_examples/Gyvel_741_0.5404509437352878_GT_2020-08-24T09_04_34.000Z_CT_1597334311.825591_9.397231333_55.9532905.jpg]

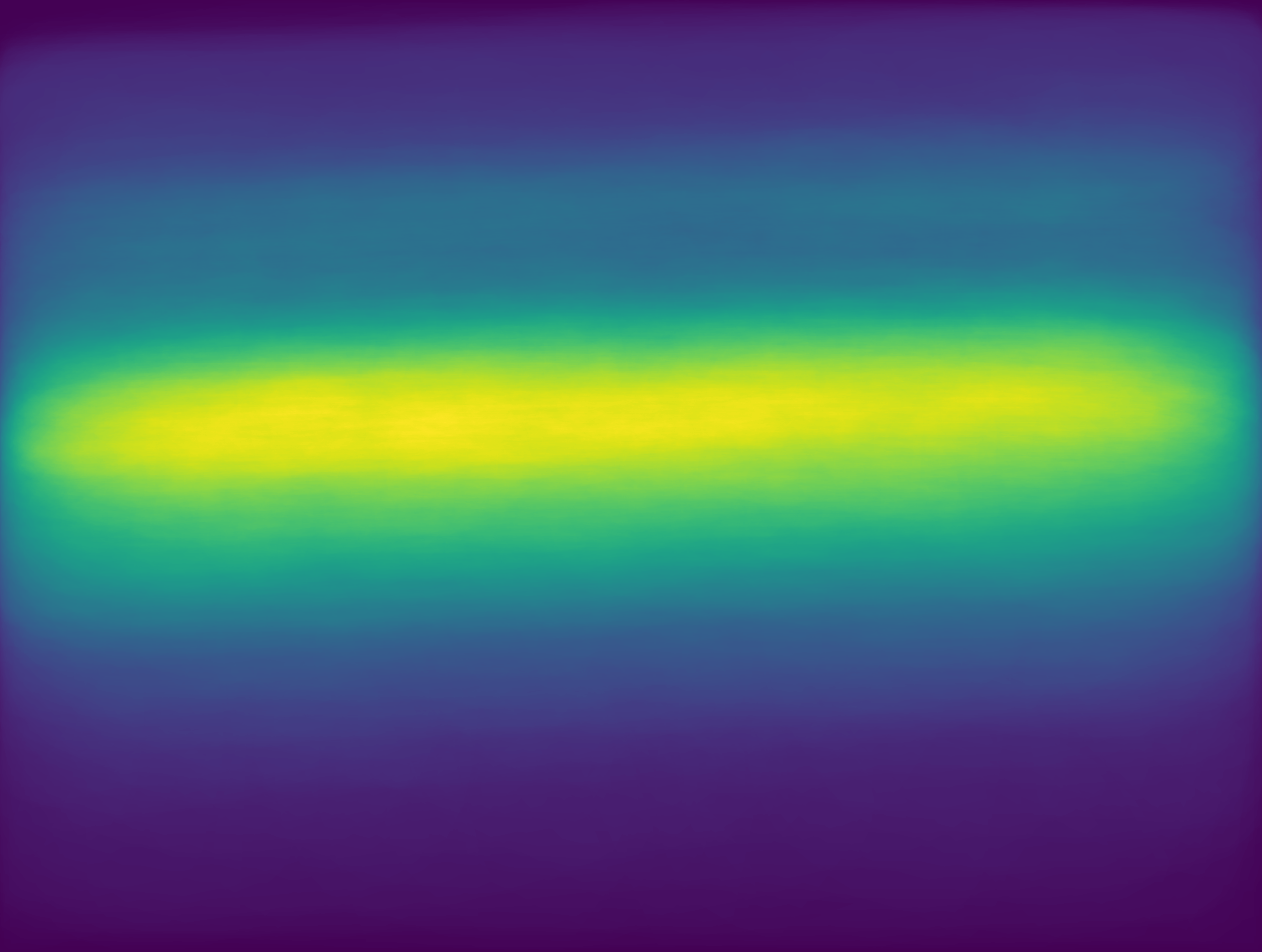

Supplement: Supplementary file 1 [file sensors-21-06126-s001.zip › images/class_examples/heatmap_Gyldenris.png]

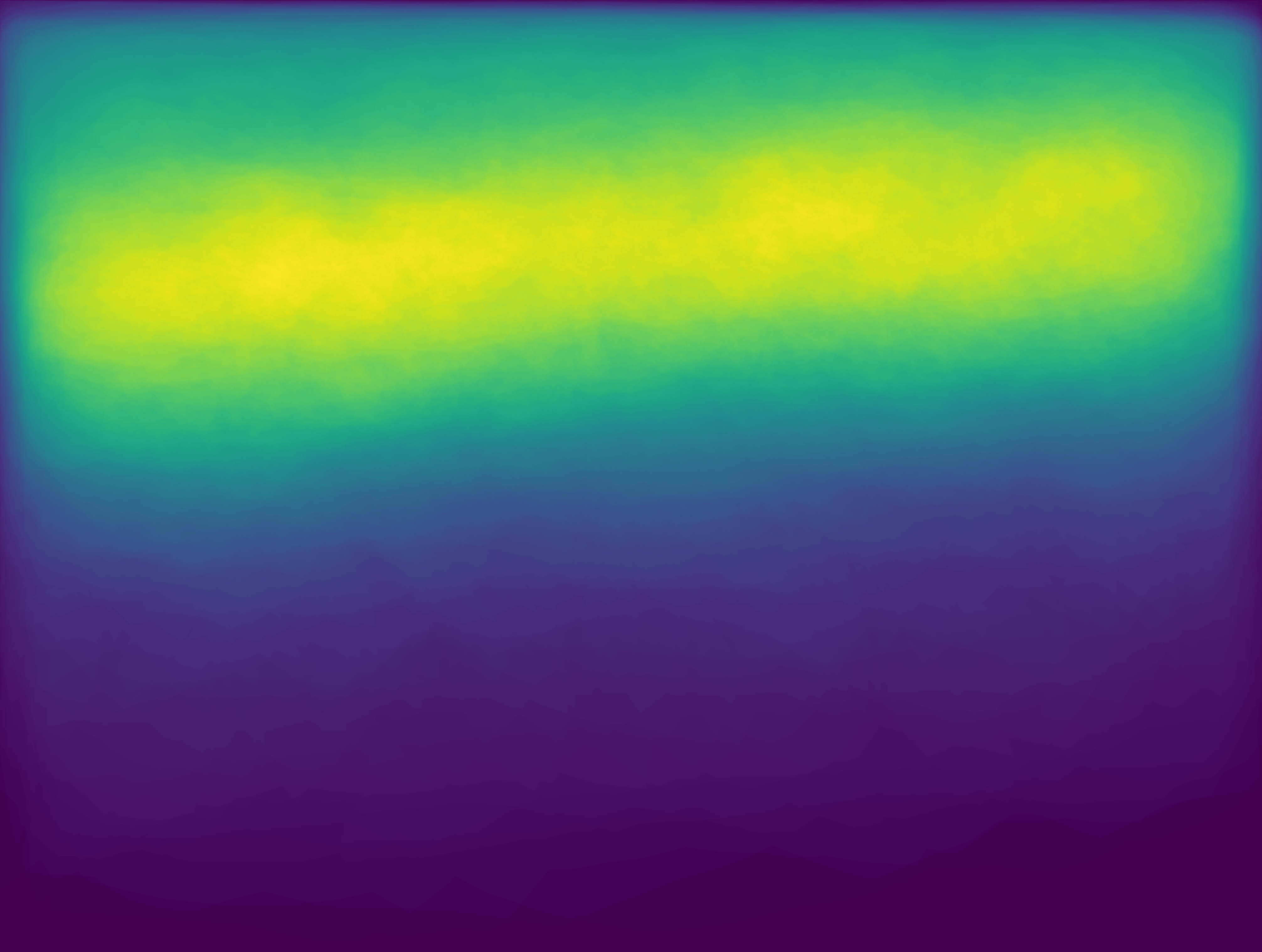

Supplement: Supplementary file 1 [file sensors-21-06126-s001.zip › images/class_examples/heatmap_Gyvel.png]

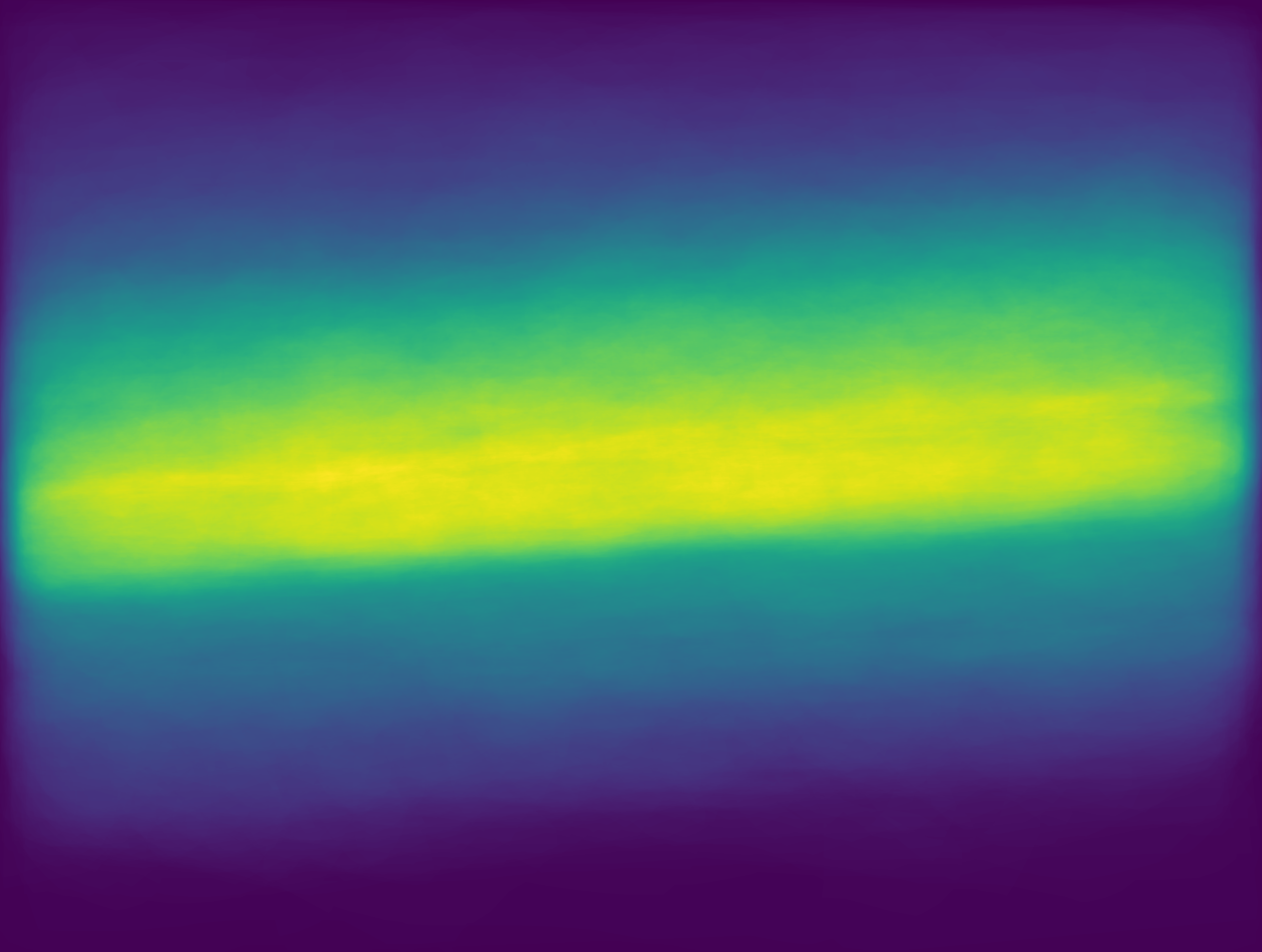

Supplement: Supplementary file 1 [file sensors-21-06126-s001.zip › images/class_examples/heatmap_Hyben.png]

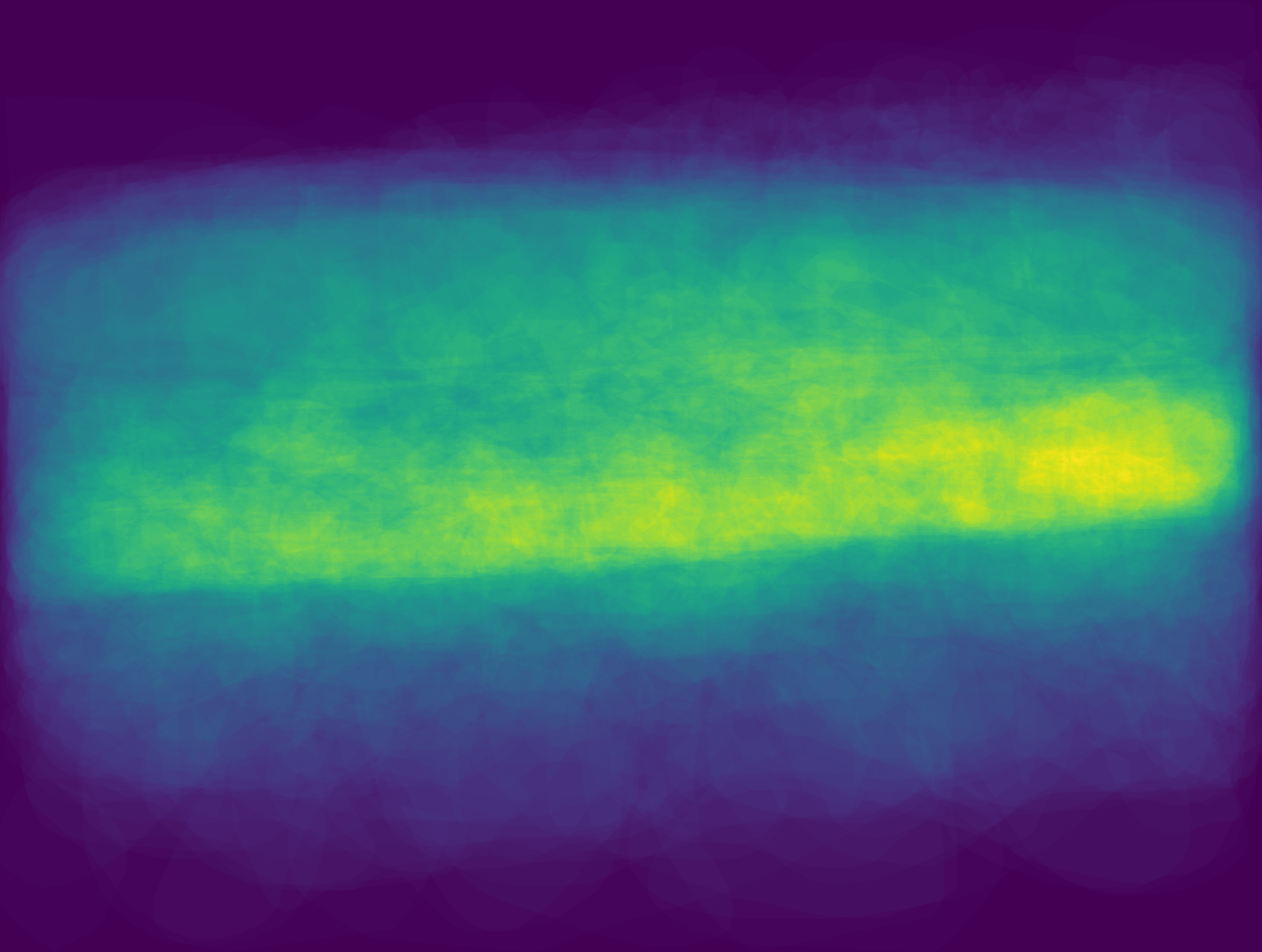

Supplement: Supplementary file 1 [file sensors-21-06126-s001.zip › images/class_examples/heatmap_Lupiner.png]

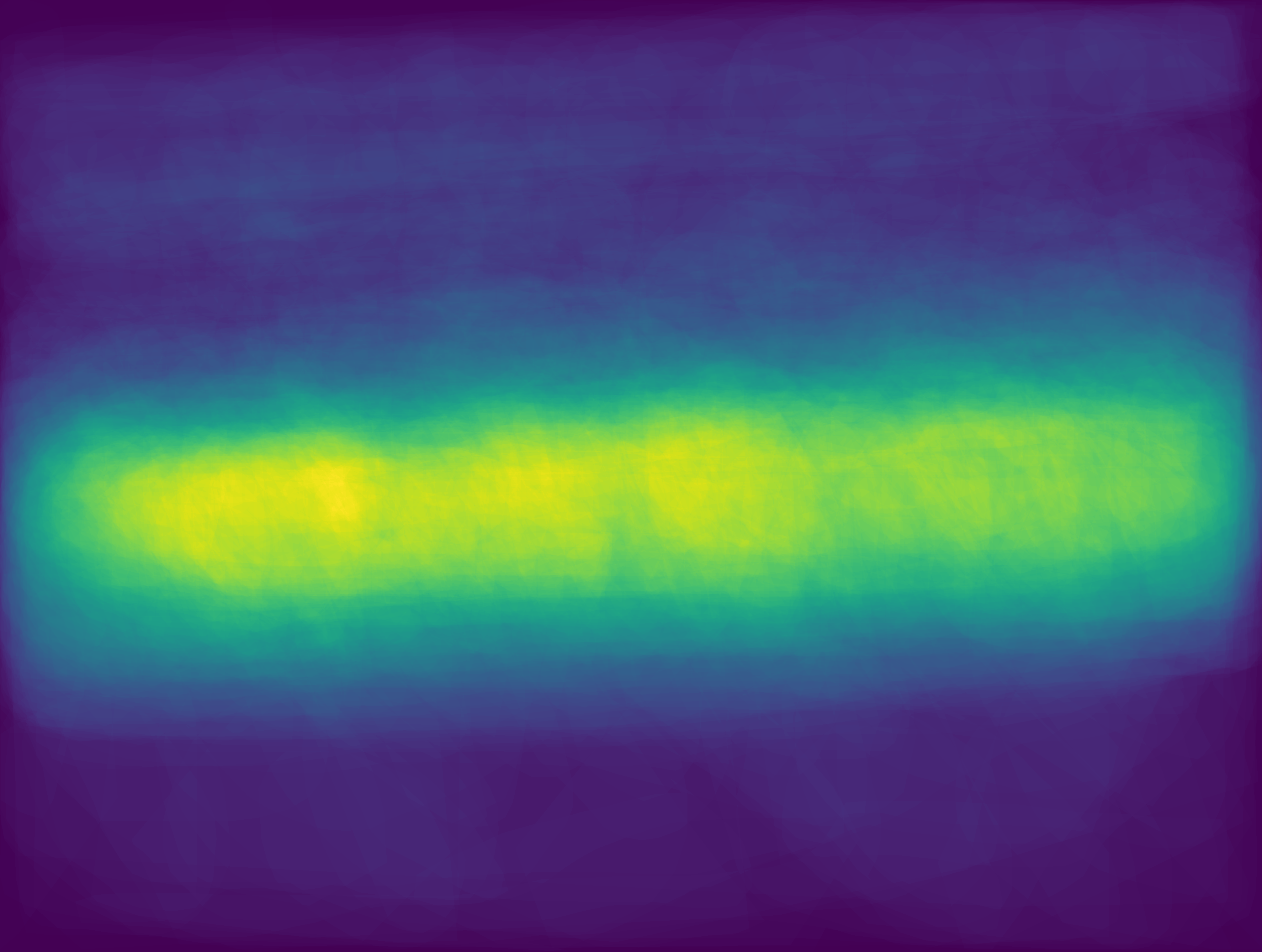

Supplement: Supplementary file 1 [file sensors-21-06126-s001.zip › images/class_examples/heatmap_Pastinak.png]

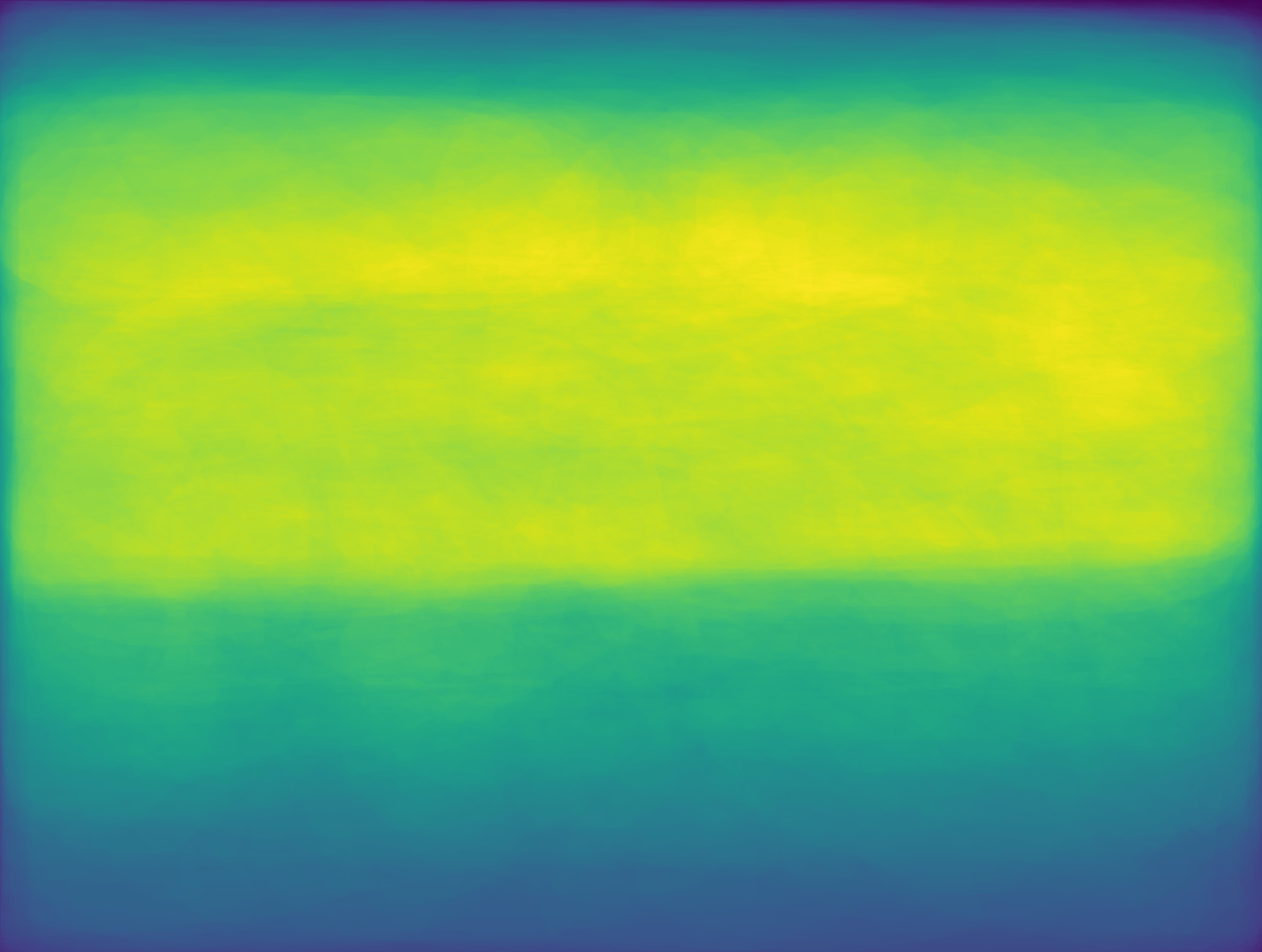

Supplement: Supplementary file 1 [file sensors-21-06126-s001.zip › images/class_examples/heatmap_Pileurt.png]

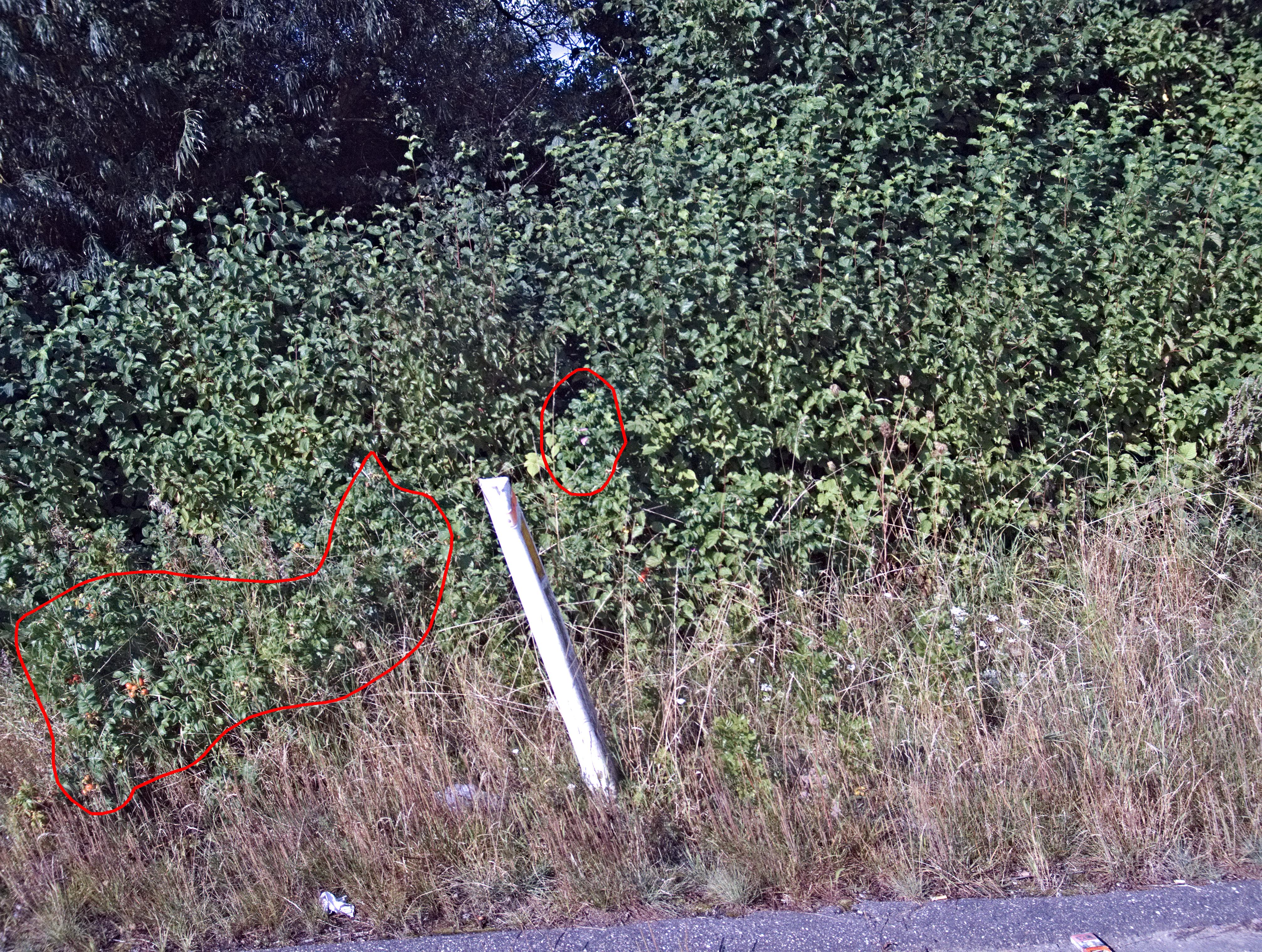

Supplement: Supplementary file 1 [file sensors-21-06126-s001.zip › images/class_examples/Hyben_357_0.06711451137486506_GT_2020-08-24T08_28_17.000Z_CT_1597332135.207674_9.648729_55.544497833.jpg]

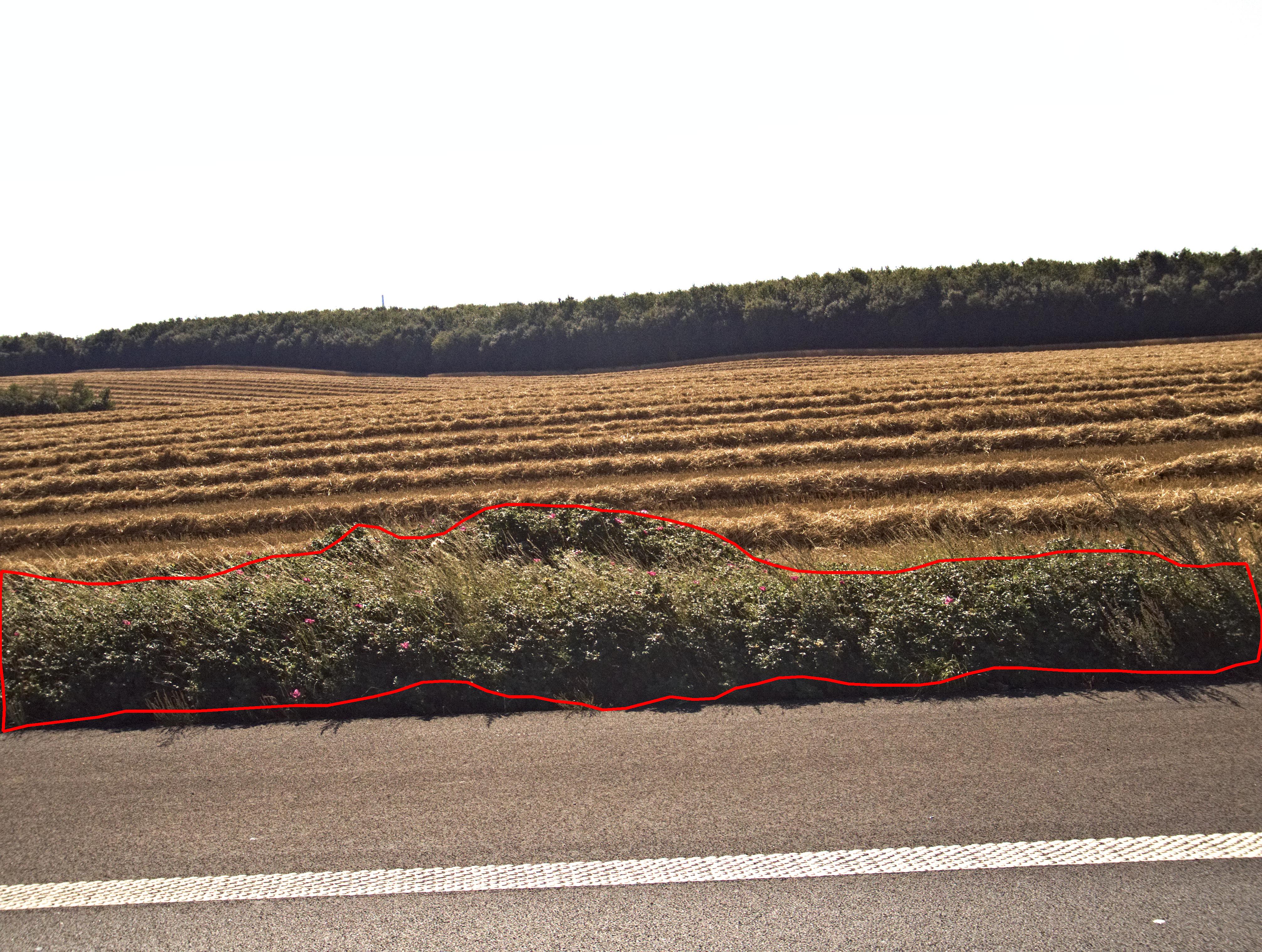

Supplement: Supplementary file 1 [file sensors-21-06126-s001.zip › images/class_examples/Hyben_713_0.1461835624026877_GT_2020-08-17T08_43_36.000Z_CT_1597329484.0175161_10.027847667_56.097095667.jpg]

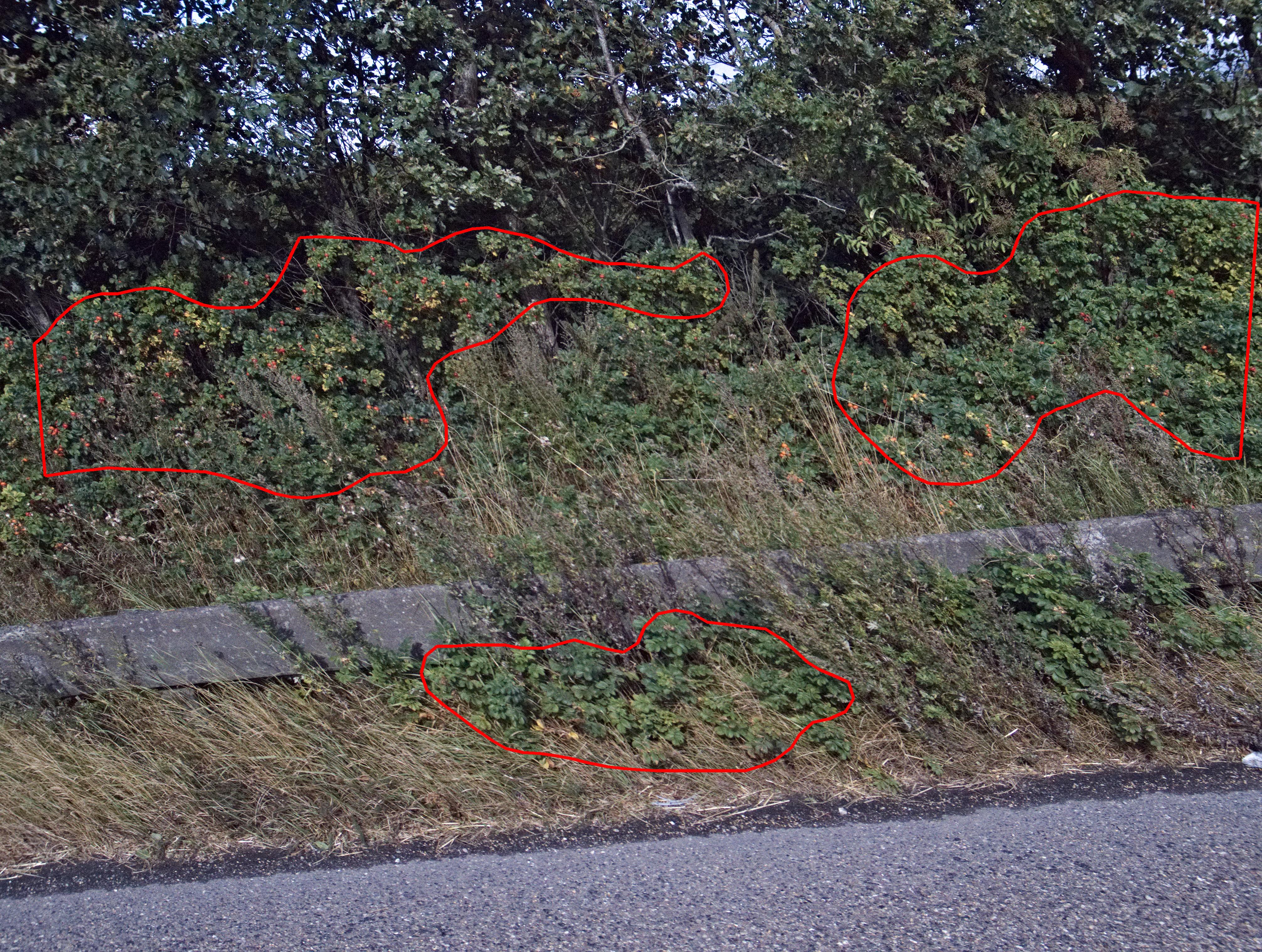

Supplement: Supplementary file 1 [file sensors-21-06126-s001.zip › images/class_examples/Hyben_832_0.19165215964503313_GT_2020-08-24T08_02_56.000Z_CT_1597330614.6324754_9.6621085_55.7821935.jpg]

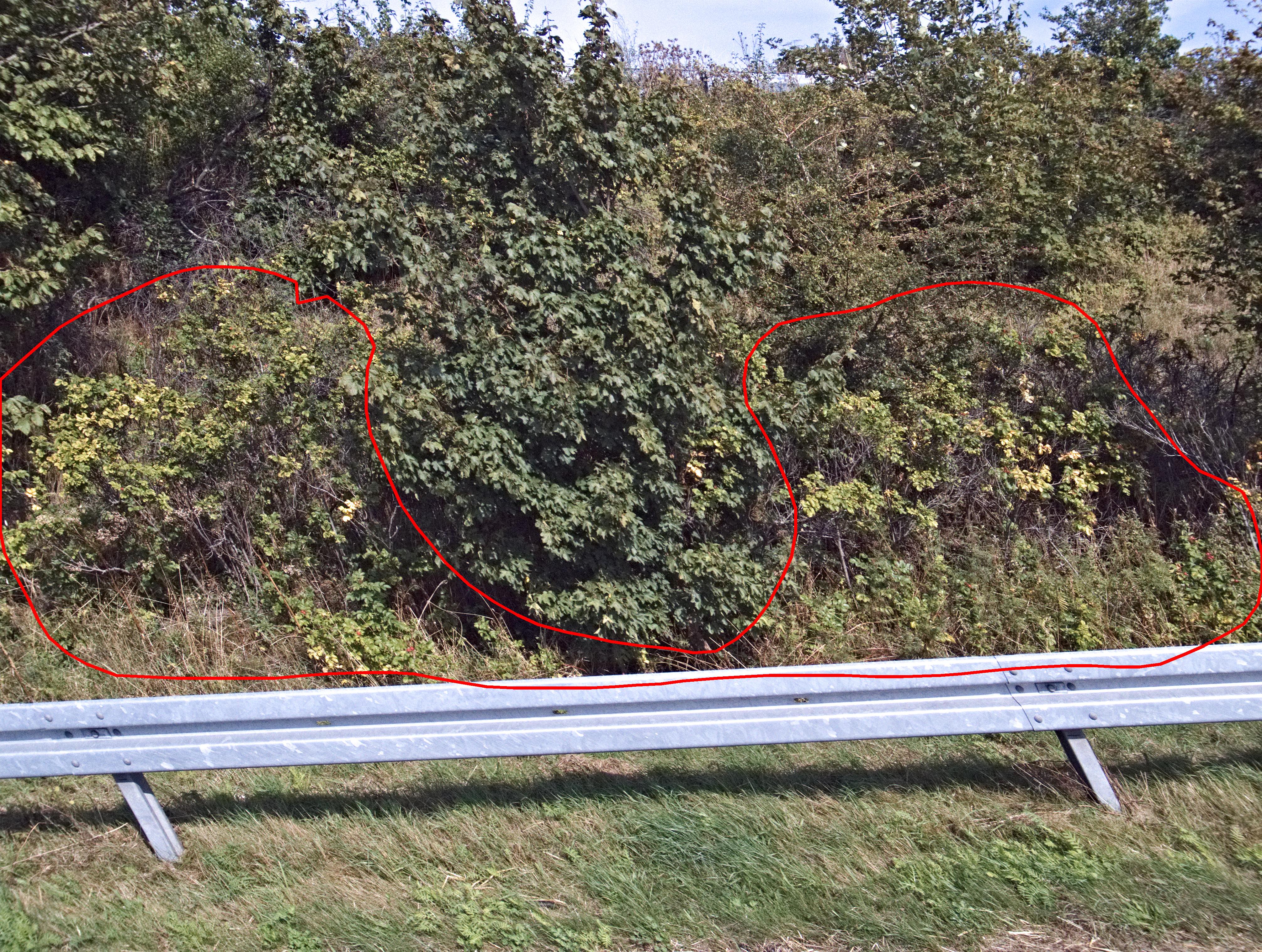

Supplement: Supplementary file 1 [file sensors-21-06126-s001.zip › images/class_examples/Hyben_951_0.26514049538769363_GT_2020-09-15T11_17_56.000Z_CT_1597345668.2125096_11.774097_55.650803167.jpg]

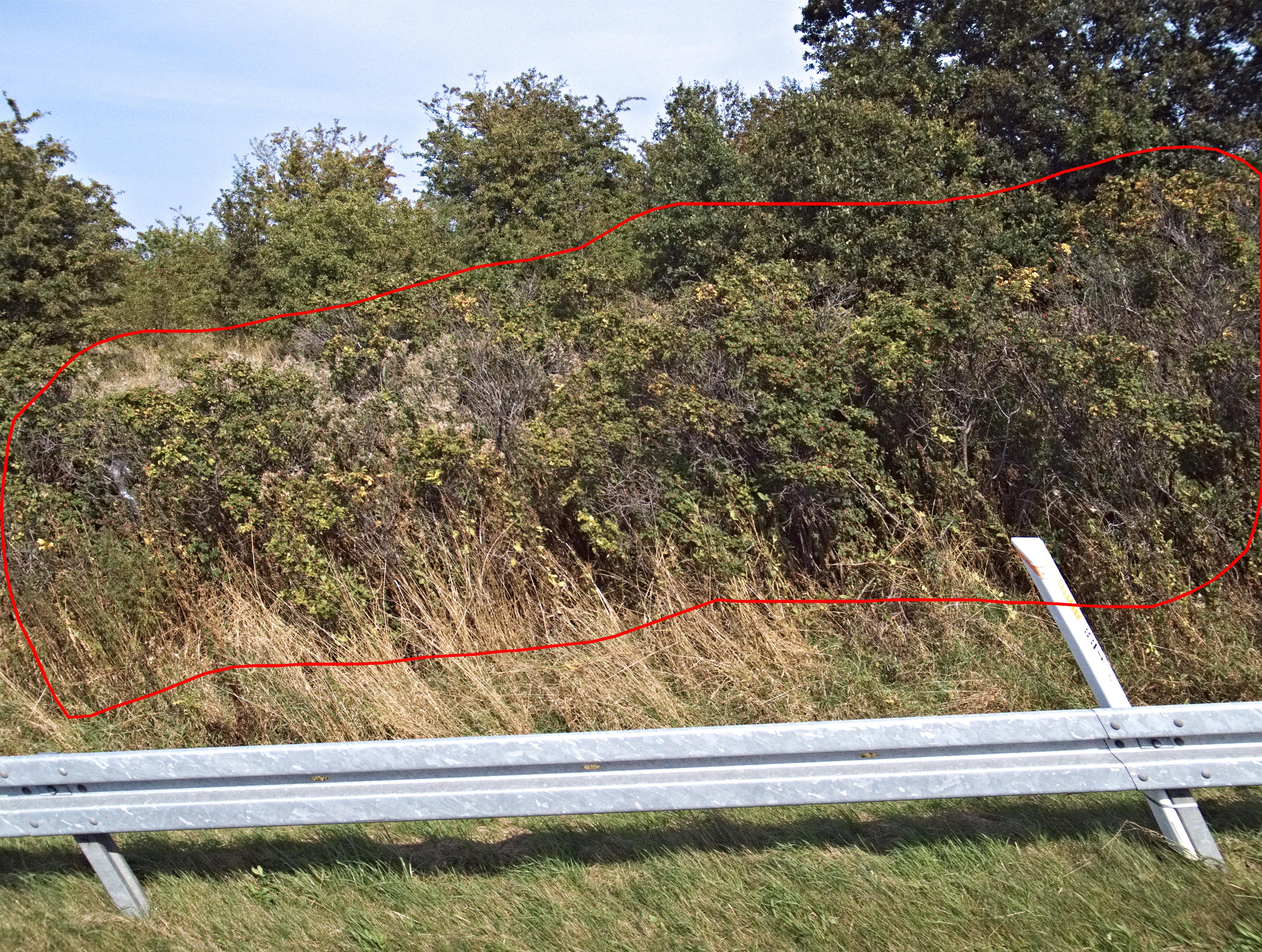

Supplement: Supplementary file 1 [file sensors-21-06126-s001.zip › images/class_examples/Hyben_1130_0.40114565532288216_GT_2020-09-15T11_17_52.000Z_CT_1597345664.2392948_11.775689833_55.6503065.jpg]

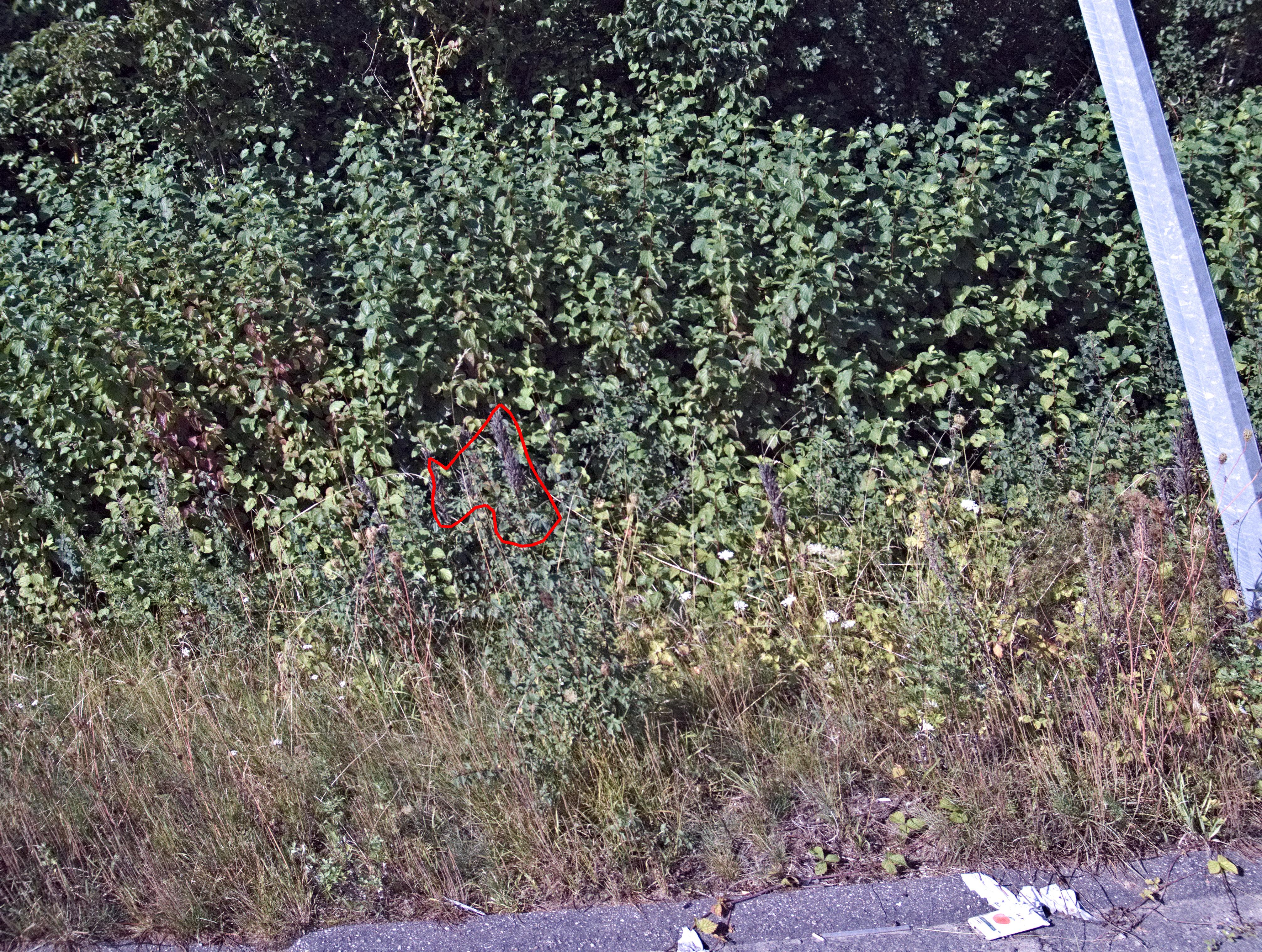

Supplement: Supplementary file 1 [file sensors-21-06126-s001.zip › images/class_examples/Lupiner_43_0.008228204082303717_GT_2020-08-24T08_28_16.000Z_CT_1597332134.4845726_9.648704_55.544591833.jpg]

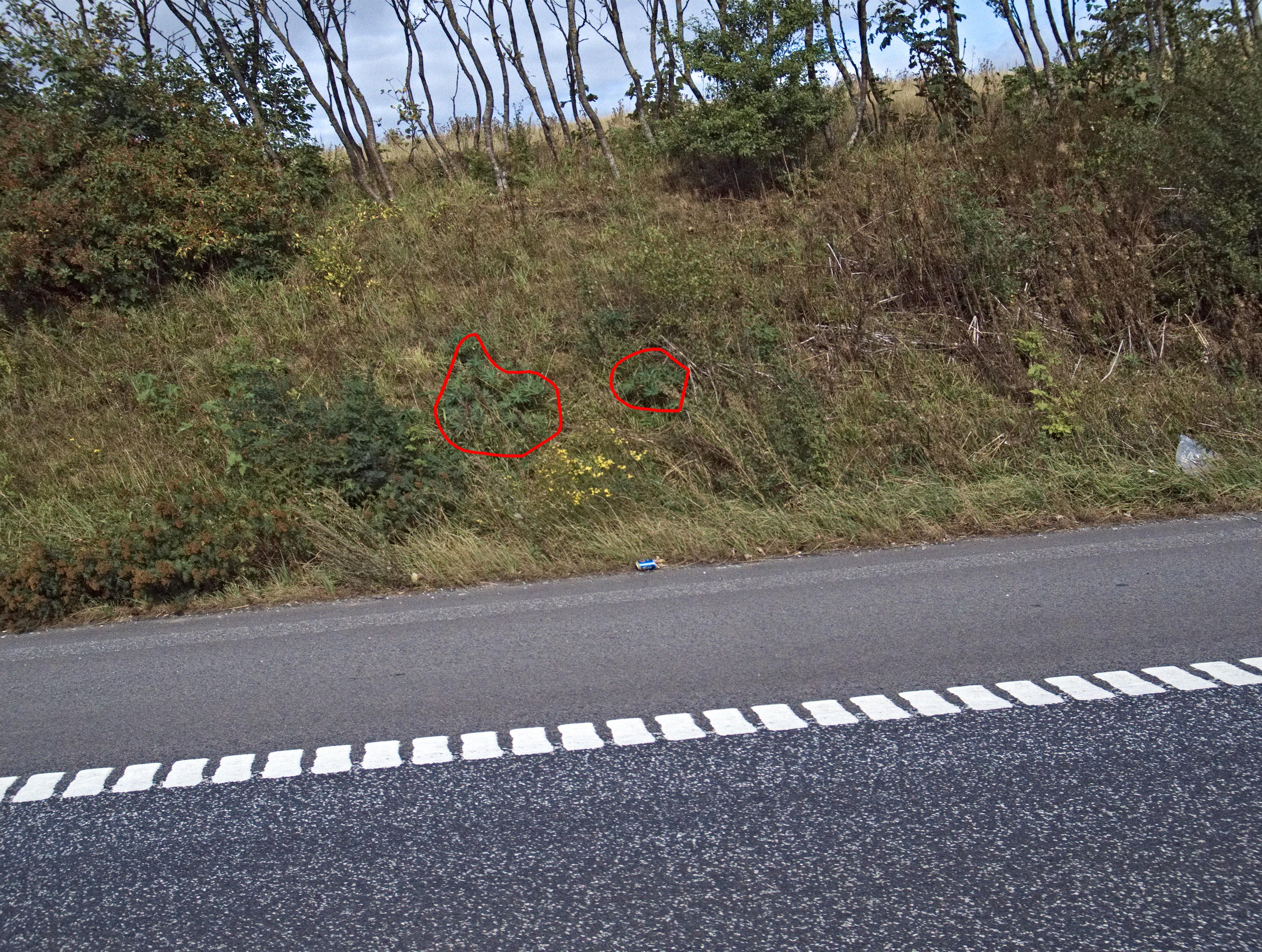

Supplement: Supplementary file 1 [file sensors-21-06126-s001.zip › images/class_examples/Lupiner_64_0.011406131441639537_GT_2020-08-24T07_56_11.000Z_CT_1597330209.1968472_9.766030167_55.866987833.jpg]

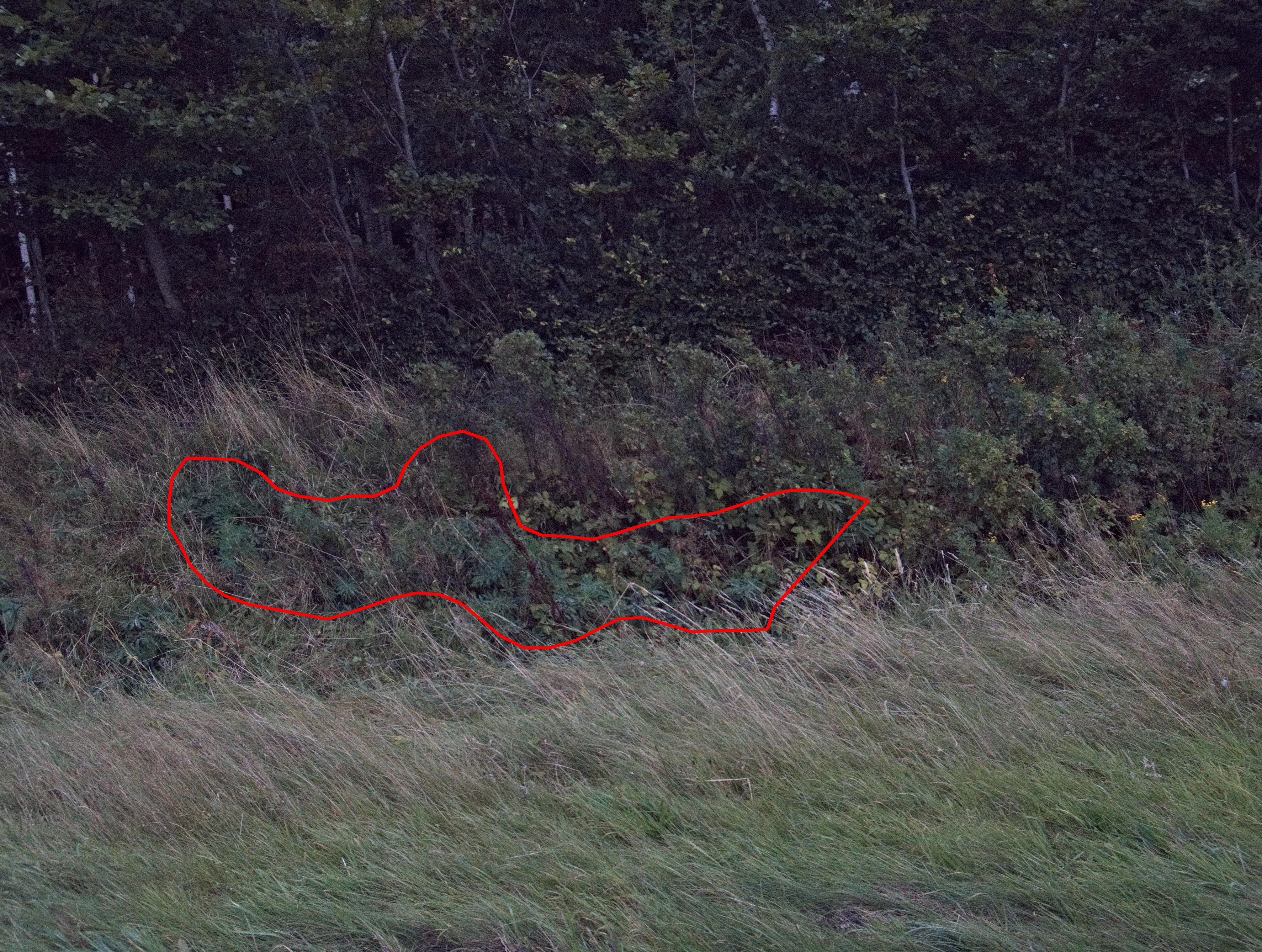

Supplement: Supplementary file 1 [file sensors-21-06126-s001.zip › images/class_examples/Lupiner_213_0.06568009641873365_GT_2020-08-24T08_59_33.000Z_CT_1597334011.0276637_9.443255167_55.9018075.jpg]

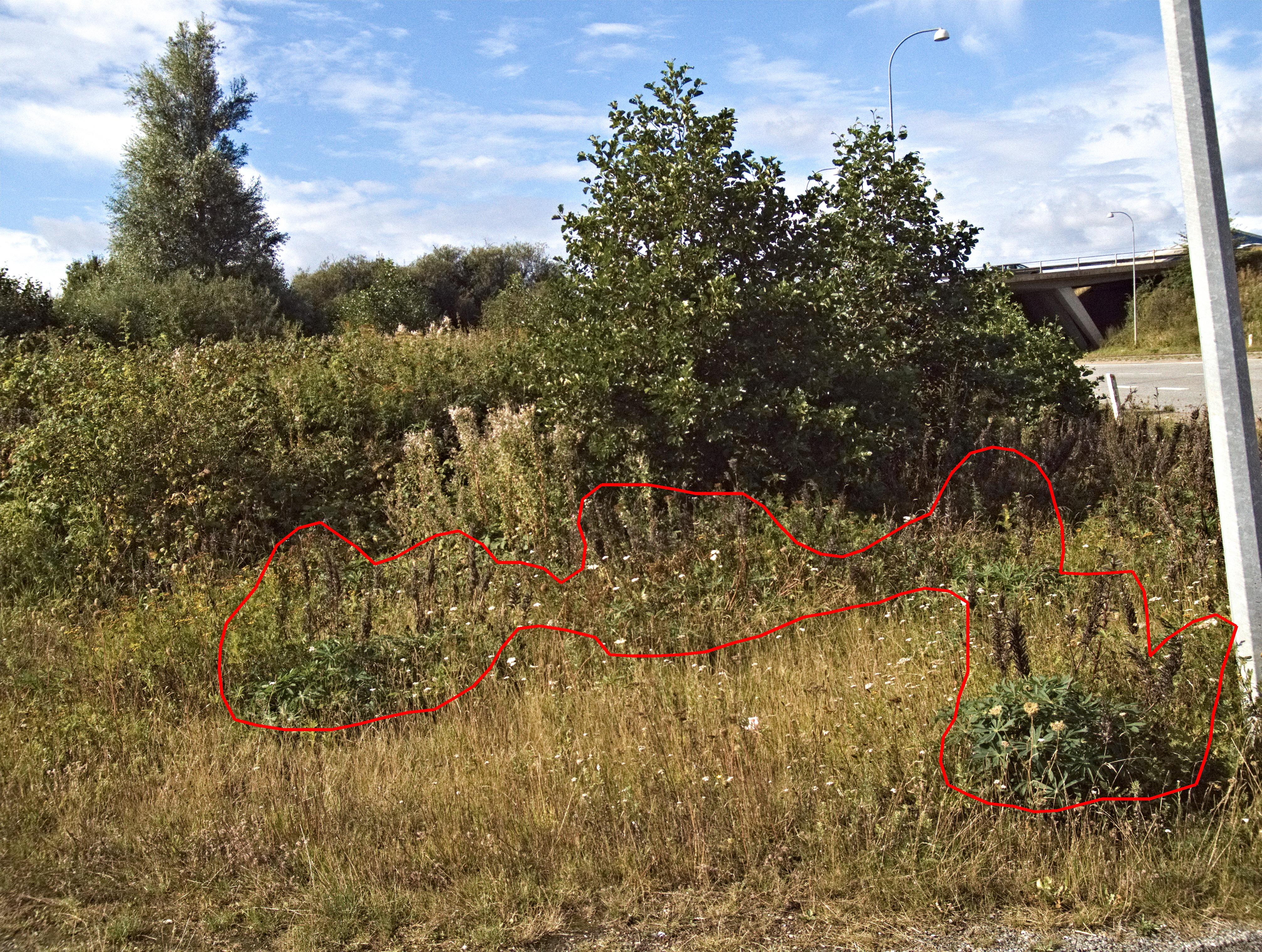

Supplement: Supplementary file 1 [file sensors-21-06126-s001.zip › images/class_examples/Lupiner_341_0.13215306473829433_GT_2020-08-24T08_28_08.000Z_CT_1597332125.9458985_9.648268833_55.545186.jpg]

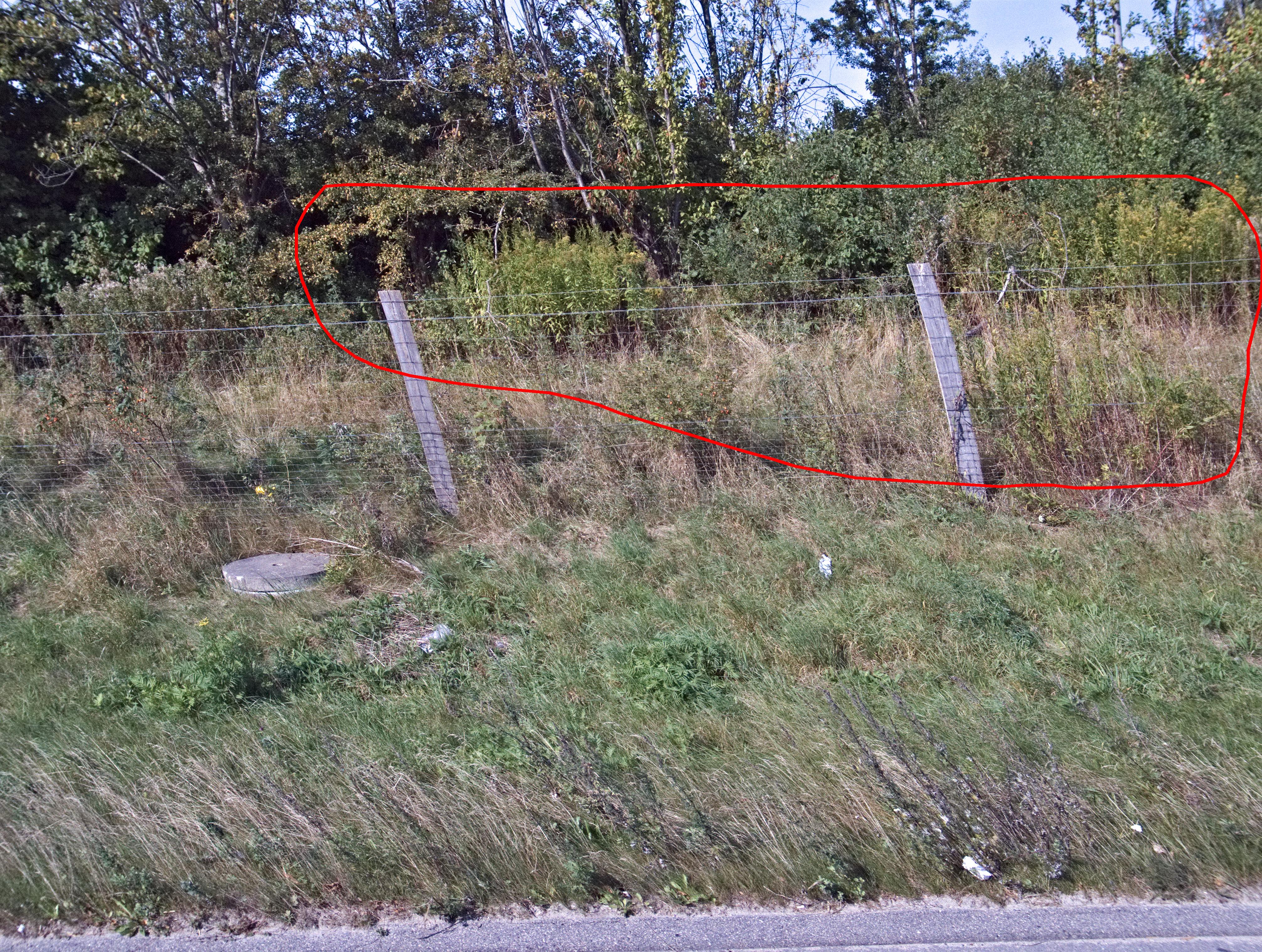

Supplement: Supplementary file 1 [file sensors-21-06126-s001.zip › images/class_examples/Lupiner_383_0.198797123907057_GT_2020-09-15T10_54_30.000Z_CT_1597344262.006176_12.348209667_55.690524667.jpg]

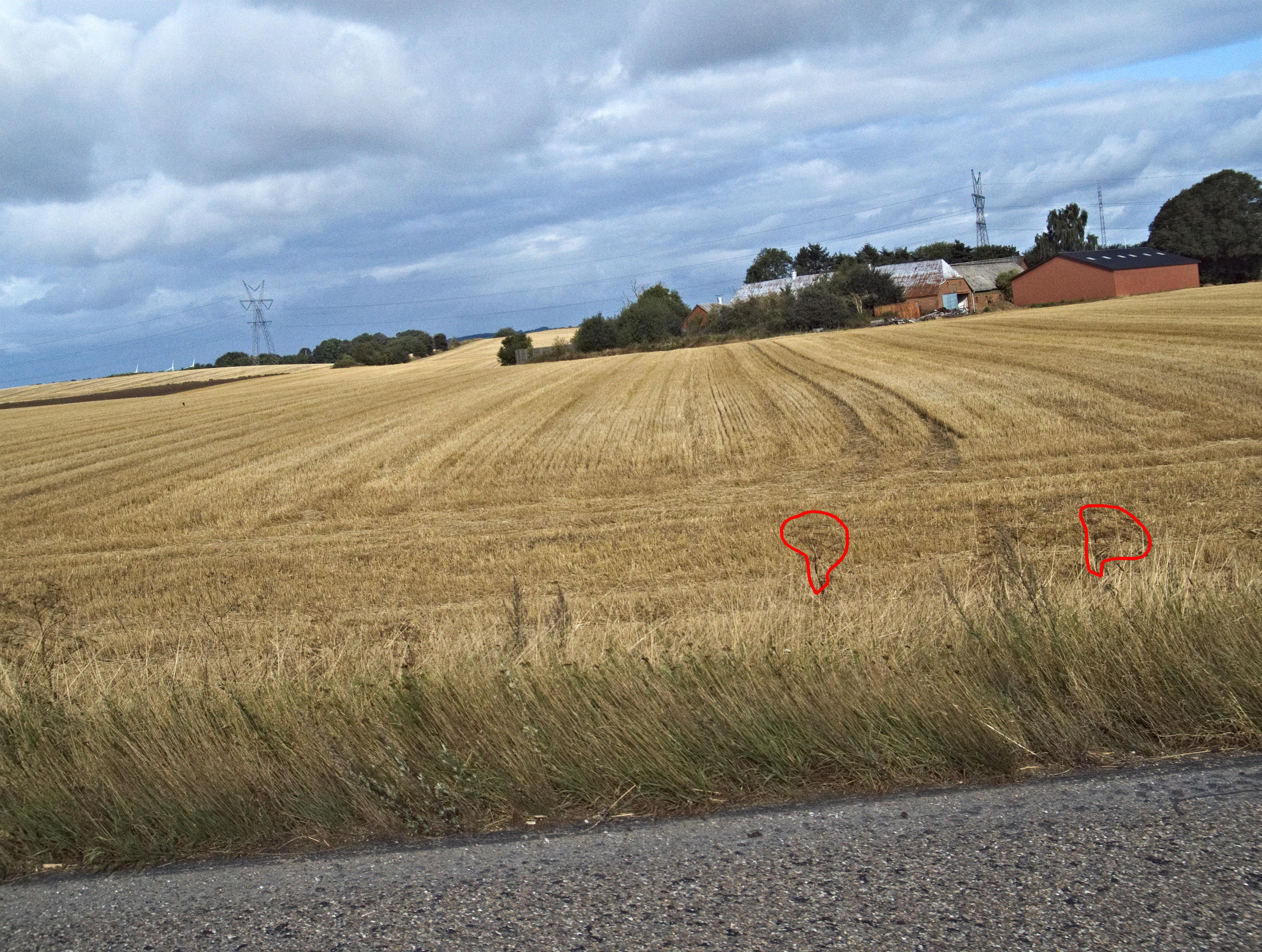

Supplement: Supplementary file 1 [file sensors-21-06126-s001.zip › images/class_examples/Pastinak_33_0.005216244529577565_GT_2020-08-24T07_57_00.000Z_CT_1597330258.7394414_9.751575333_55.857014333.jpg]

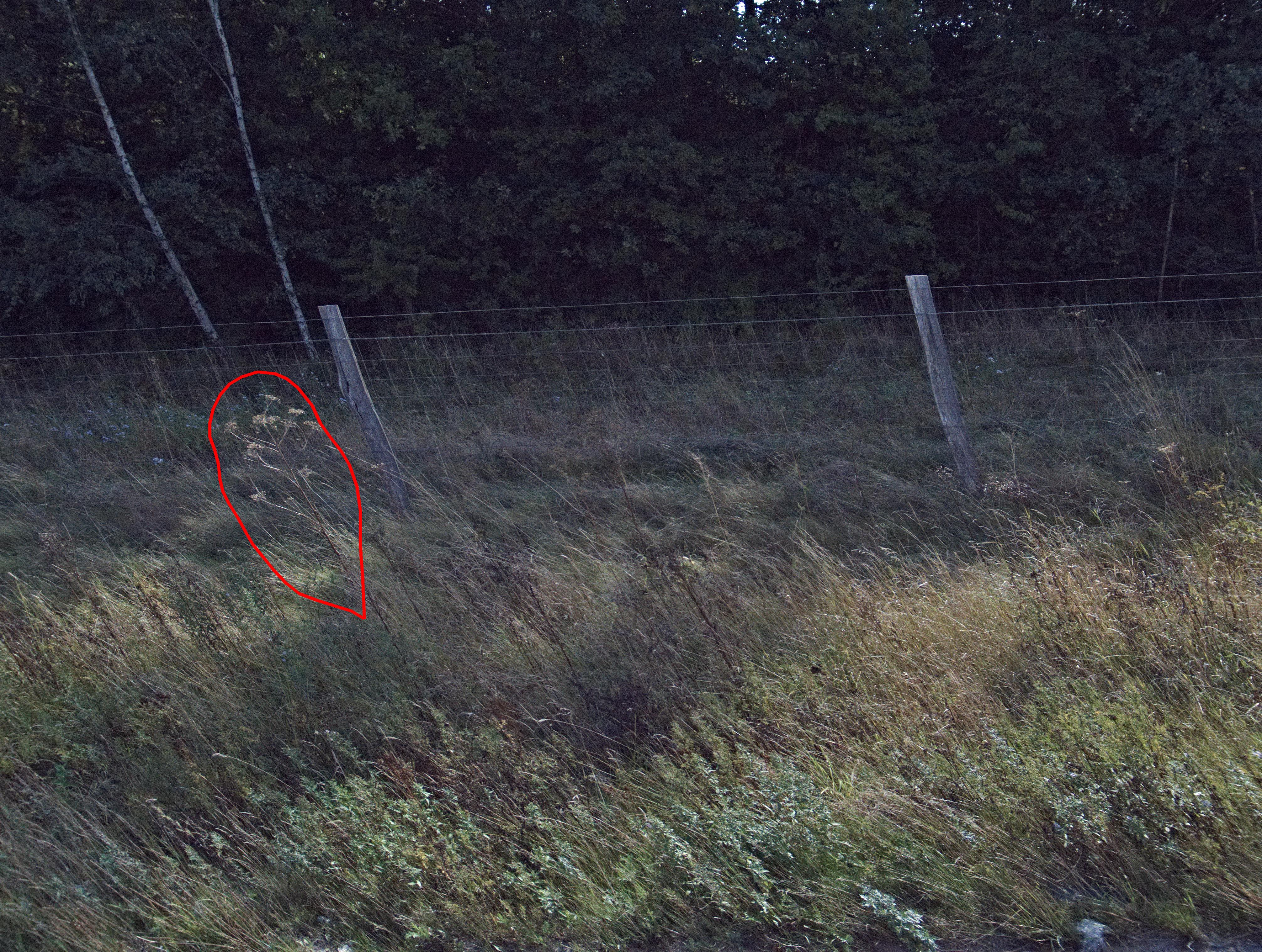

Supplement: Supplementary file 1 [file sensors-21-06126-s001.zip › images/class_examples/Pastinak_166_0.020097310615824454_GT_2020-09-15T10_13_40.000Z_CT_1597341812.0731525_12.319606167_55.670921833.jpg]

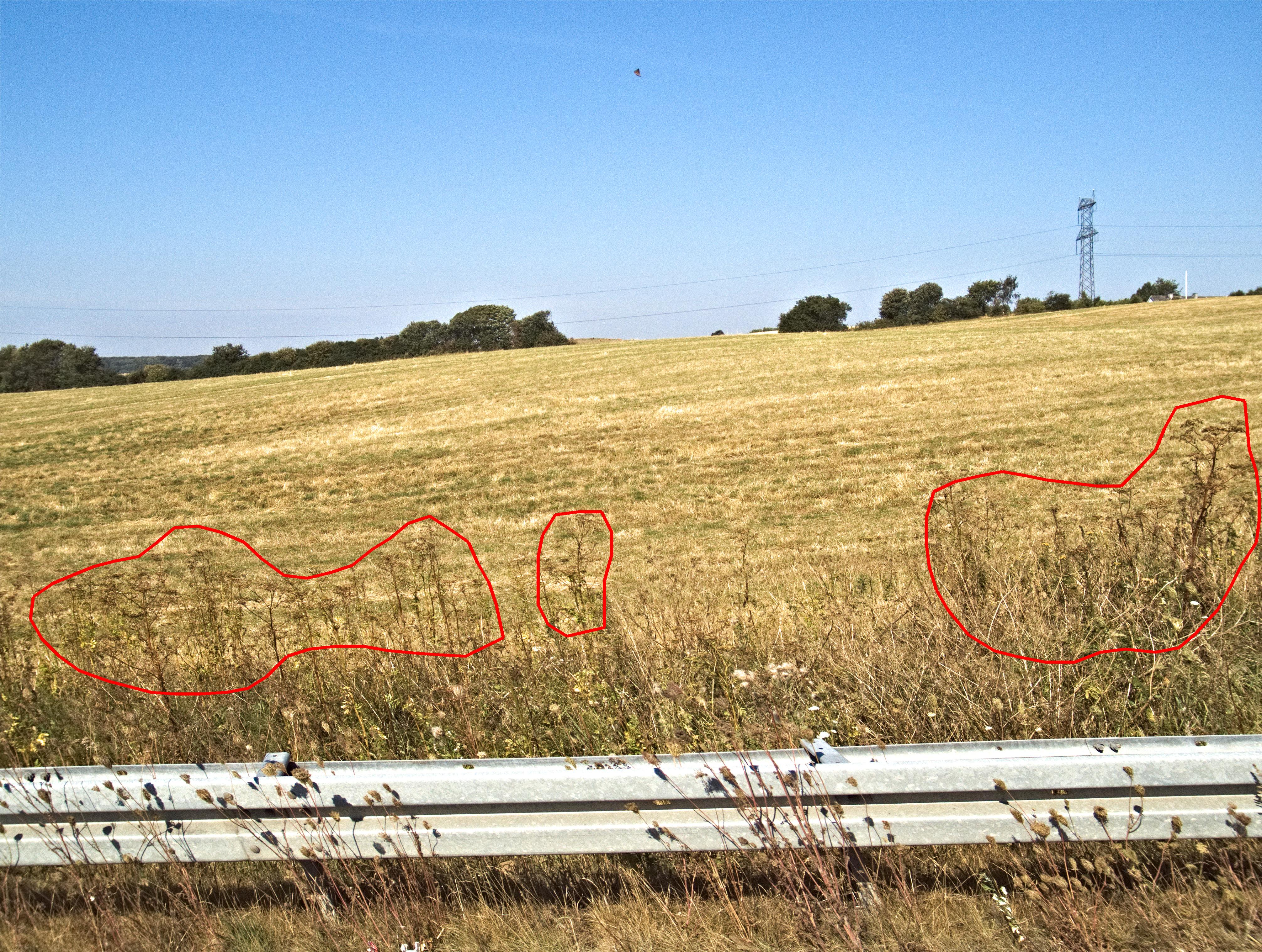

Supplement: Supplementary file 1 [file sensors-21-06126-s001.zip › images/class_examples/Pastinak_465_0.09806387741046914_GT_2020-08-17T08_55_58.000Z_CT_1597330226.1918879_10.115819667_56.221993833.jpg]

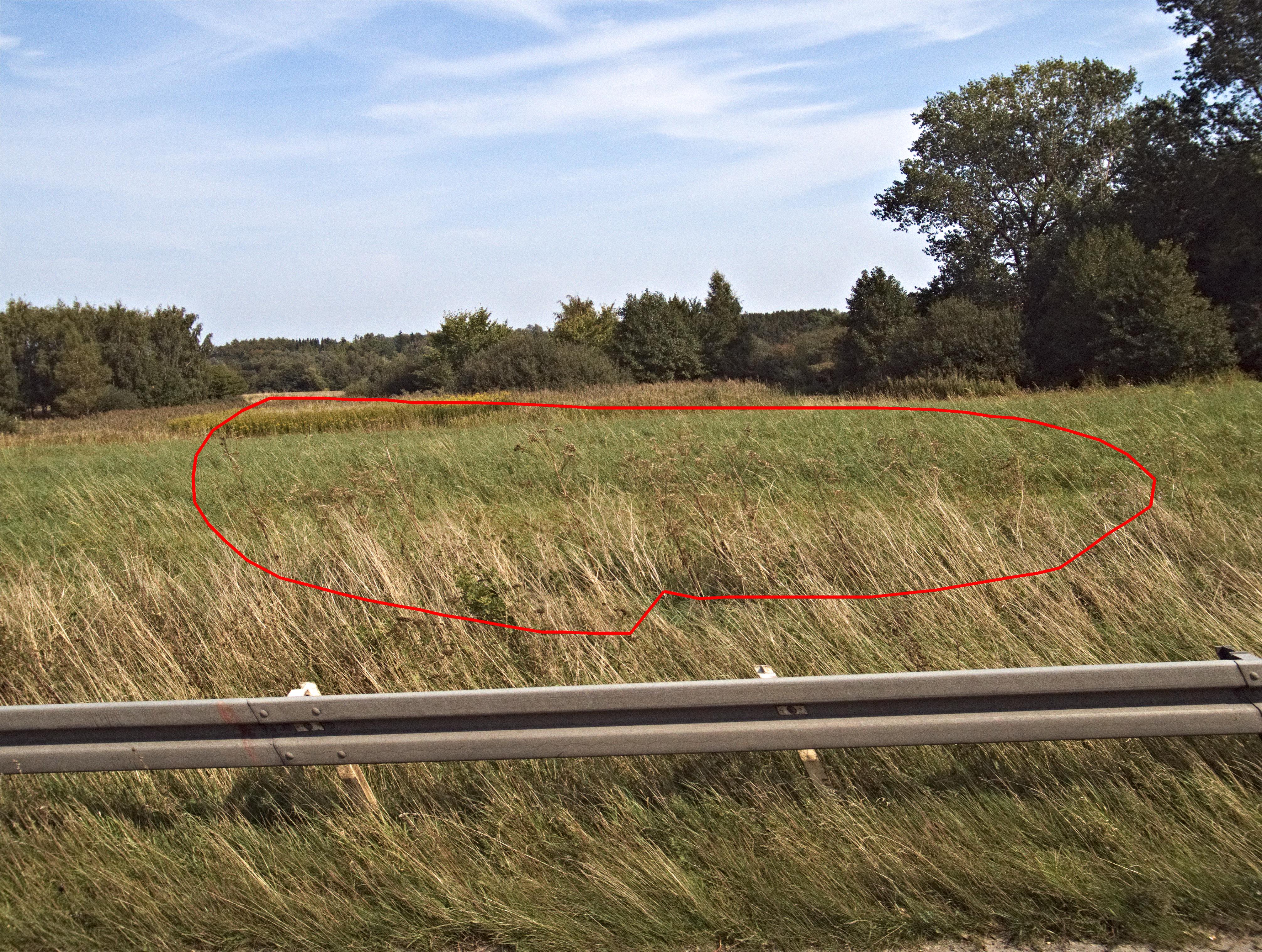

Supplement: Supplementary file 1 [file sensors-21-06126-s001.zip › images/class_examples/Pastinak_532_0.14166859297862505_GT_2020-09-15T11_10_44.000Z_CT_1597345235.5461717_11.9625155_55.633491.jpg]

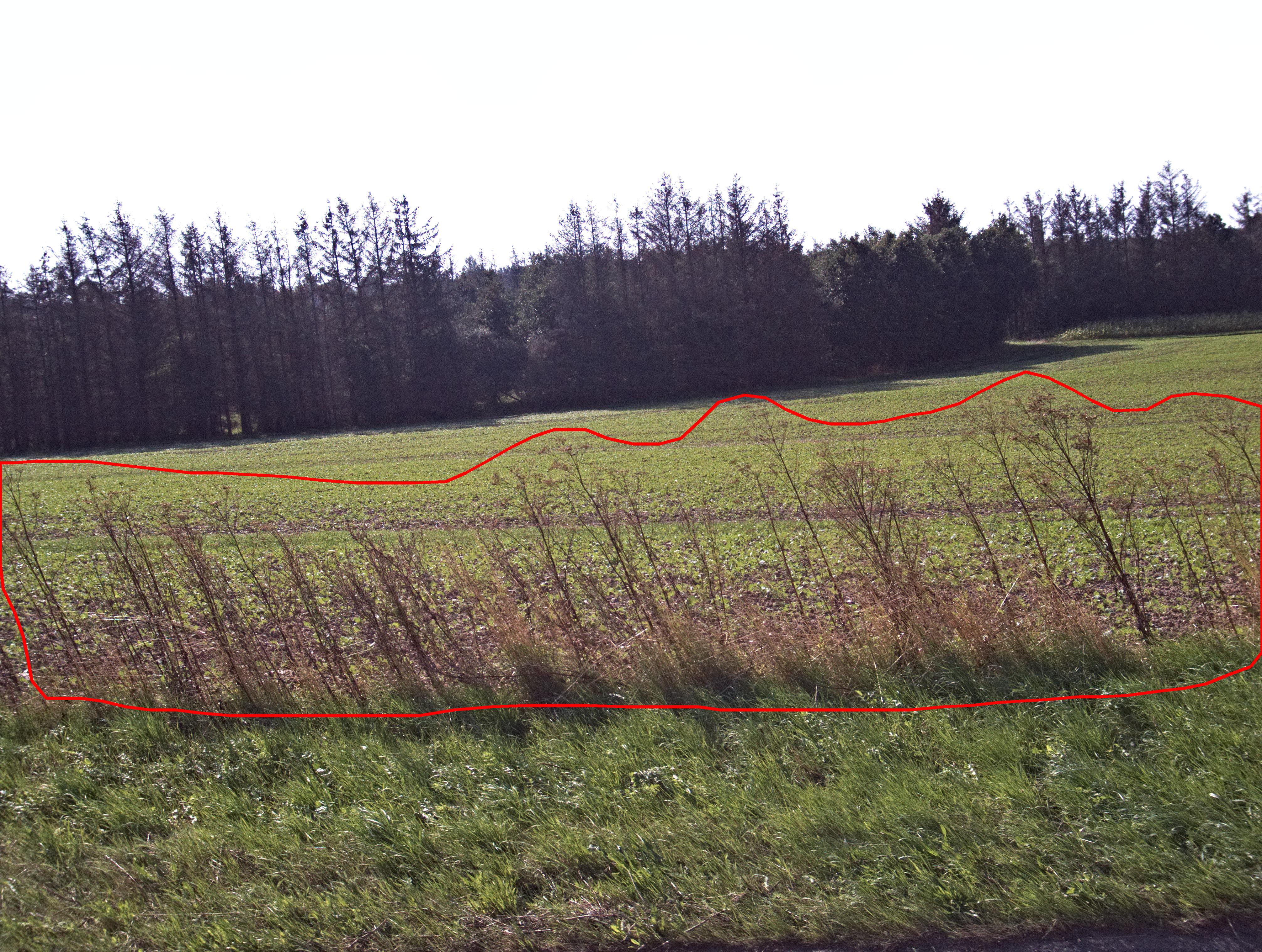

Supplement: Supplementary file 1 [file sensors-21-06126-s001.zip › images/class_examples/Pastinak_632_0.27846856659480645_GT_2020-09-15T09_32_14.000Z_CT_1597339325.6265533_11.4623155_55.432969667.jpg]

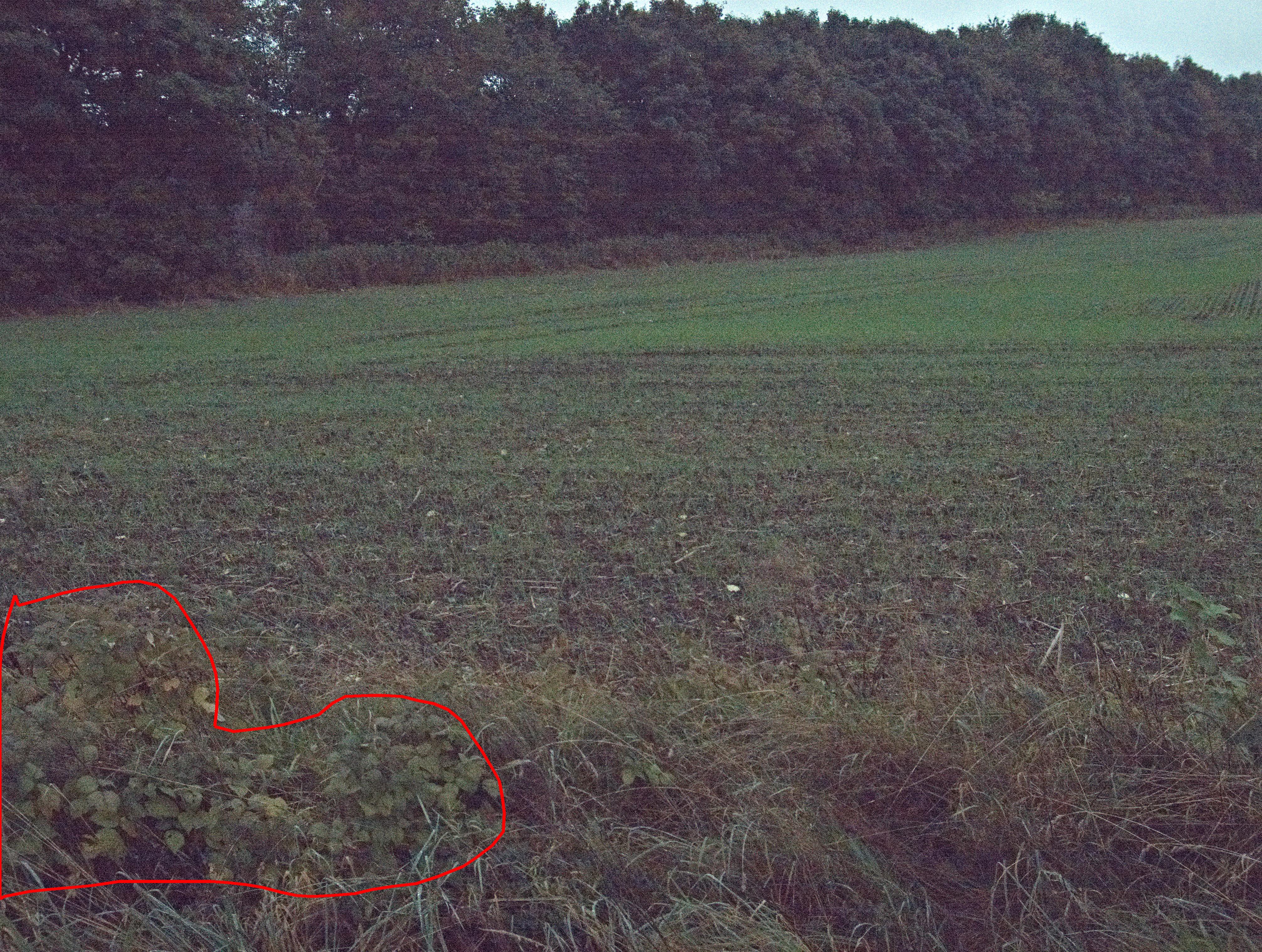

Supplement: Supplementary file 1 [file sensors-21-06126-s001.zip › images/class_examples/Pileurt_77_0.09016447418120337_GT_2020-10-06T08_27_13.000Z_CT_1597332322.4913309_9.629831833_55.9834515.jpg]

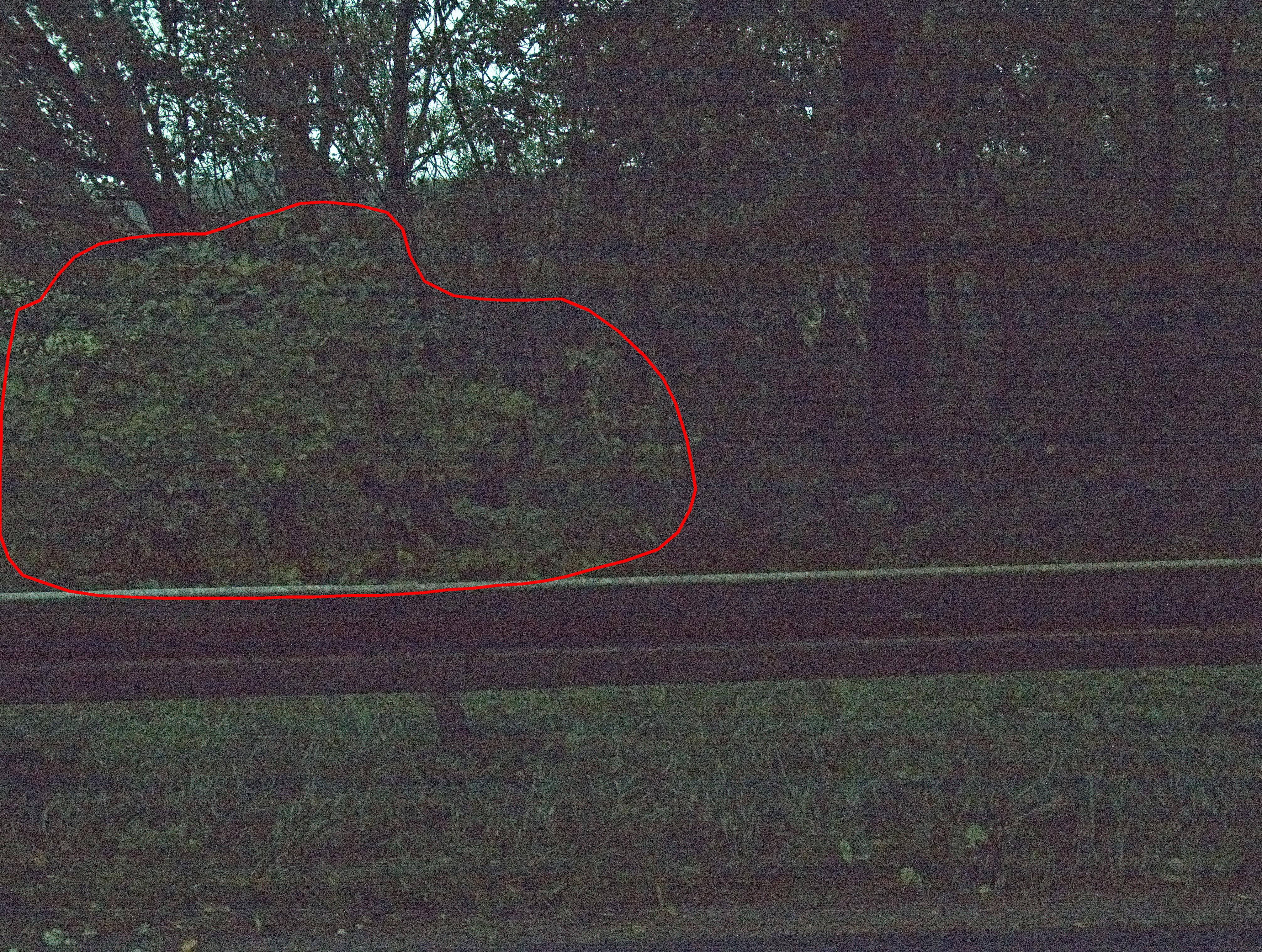

Supplement: Supplementary file 1 [file sensors-21-06126-s001.zip › images/class_examples/Pileurt_204_0.17937357767397205_GT_2020-10-06T08_27_20.000Z_CT_1597332329.148792_9.631019167_55.98379.jpg]

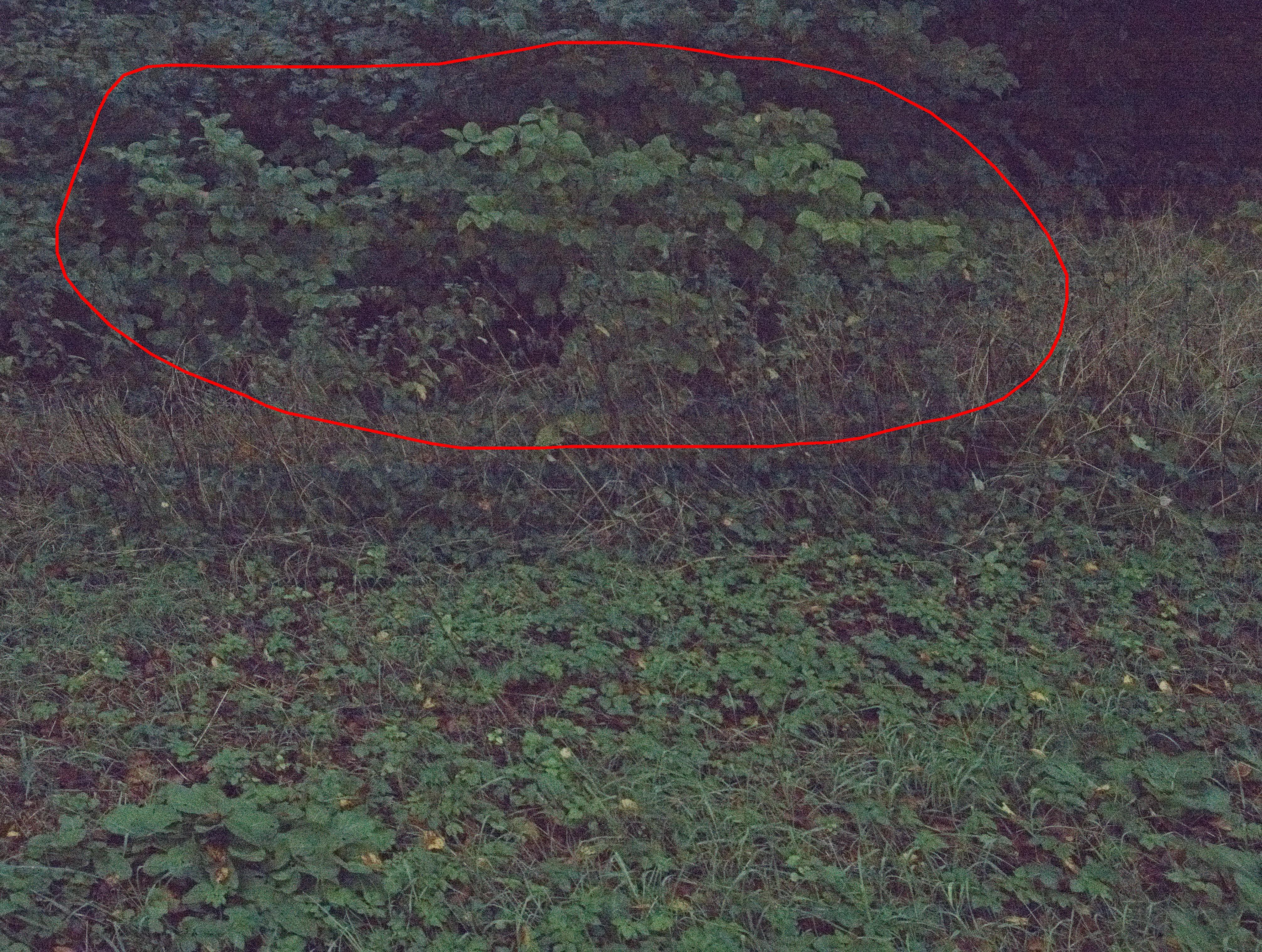

Supplement: Supplementary file 1 [file sensors-21-06126-s001.zip › images/class_examples/Pileurt_281_0.27674237986796757_GT_2020-10-06T08_31_15.000Z_CT_1597332564.0042334_9.620988667_55.974478667.jpg]

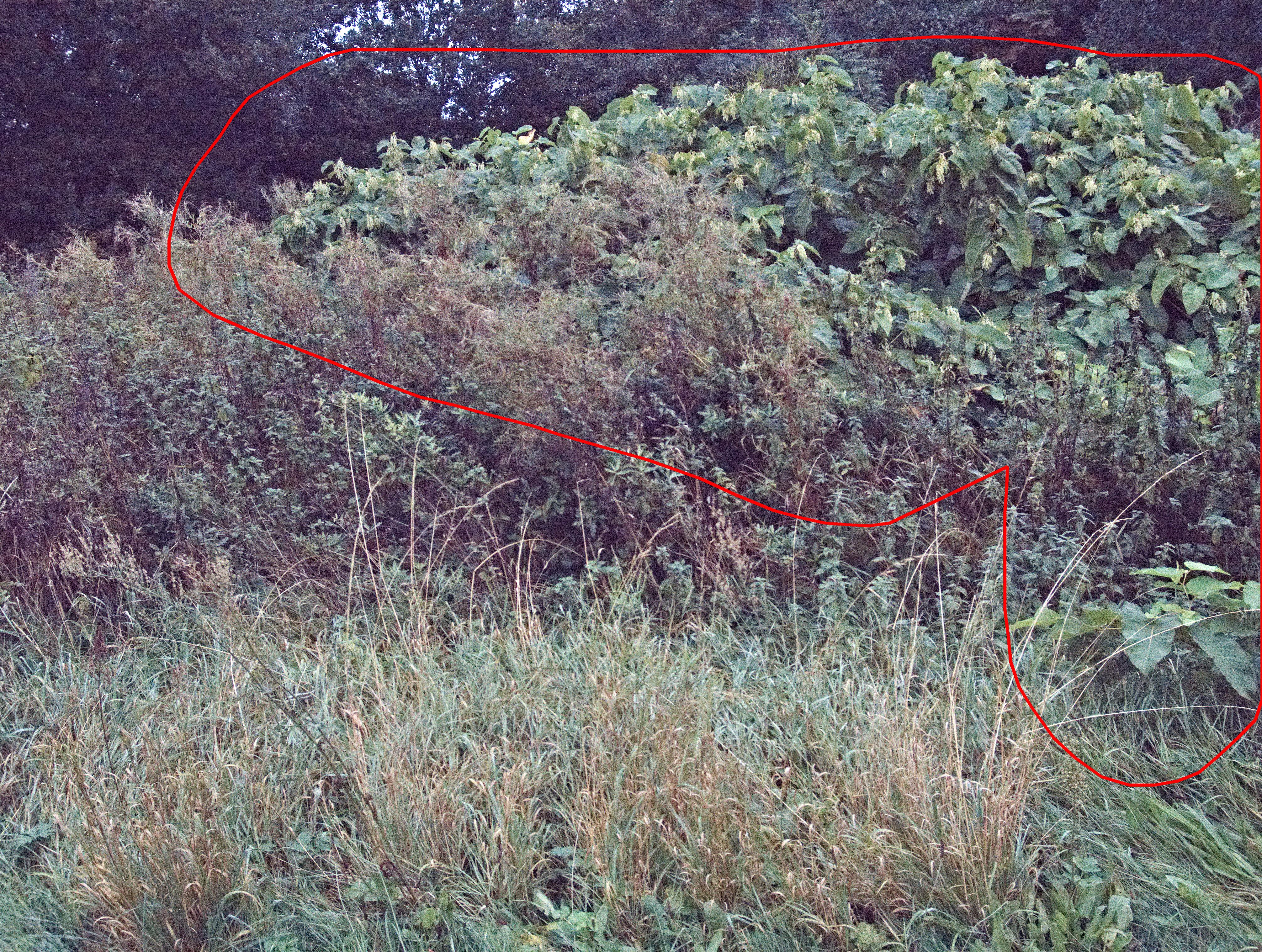

Supplement: Supplementary file 1 [file sensors-21-06126-s001.zip › images/class_examples/Pileurt_332_0.4068635928720352_GT_2020-10-06T09_50_33.000Z_CT_1597337322.243722_9.8422205_56.0042935.jpg]

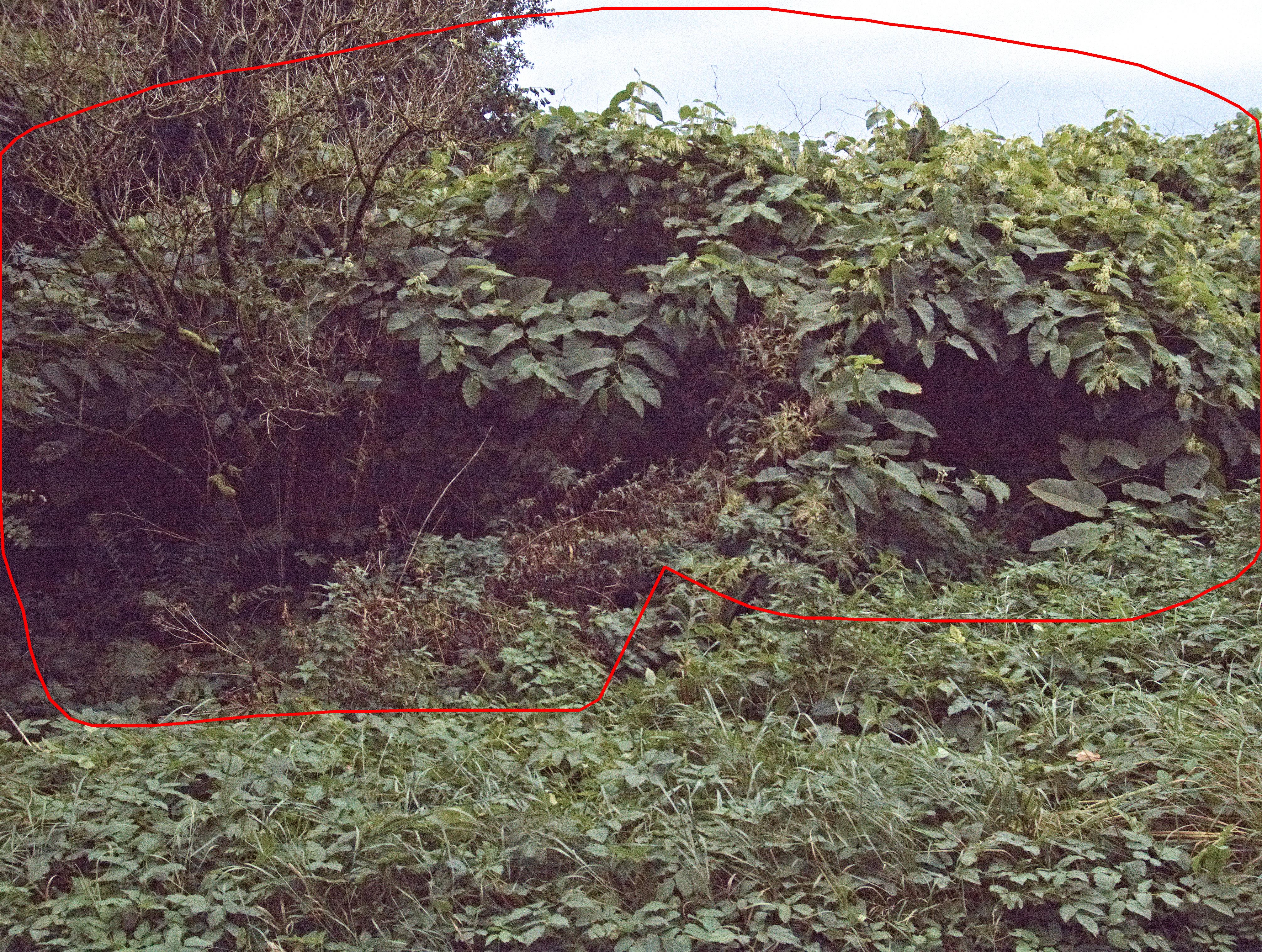

Supplement: Supplementary file 1 [file sensors-21-06126-s001.zip › images/class_examples/Pileurt_383_0.6338593705206482_GT_2020-10-06T09_50_42.000Z_CT_1597337331.4076703_9.842583167_56.004198.jpg]

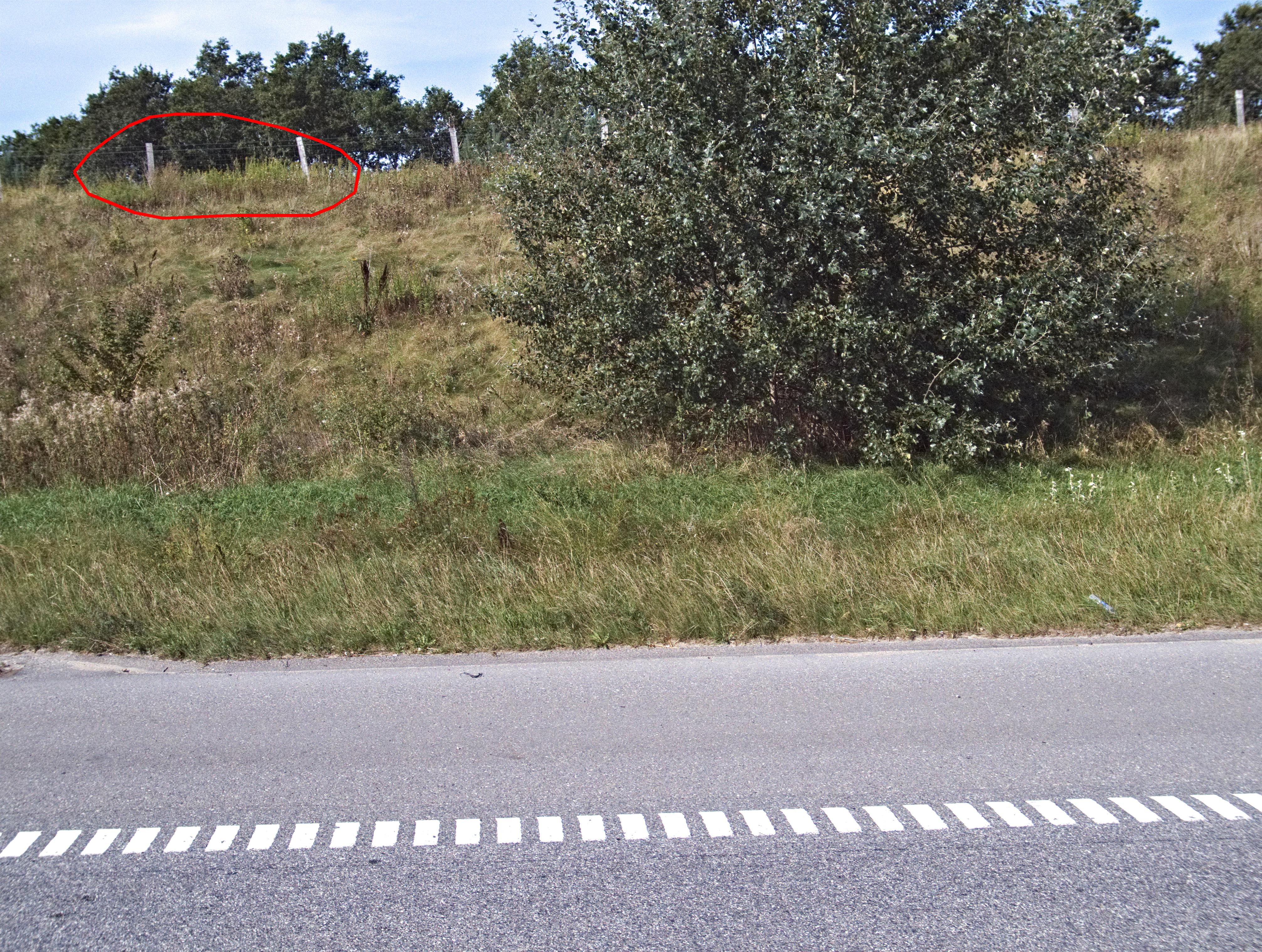

Supplement: Supplementary file 1 [file sensors-21-06126-s001.zip › images/class_examples/Gyldenris_224_0.018799931129476588_GT_2020-09-15T10_53_46.000Z_CT_1597344217.5893047_12.360995333_55.696832.jpg]

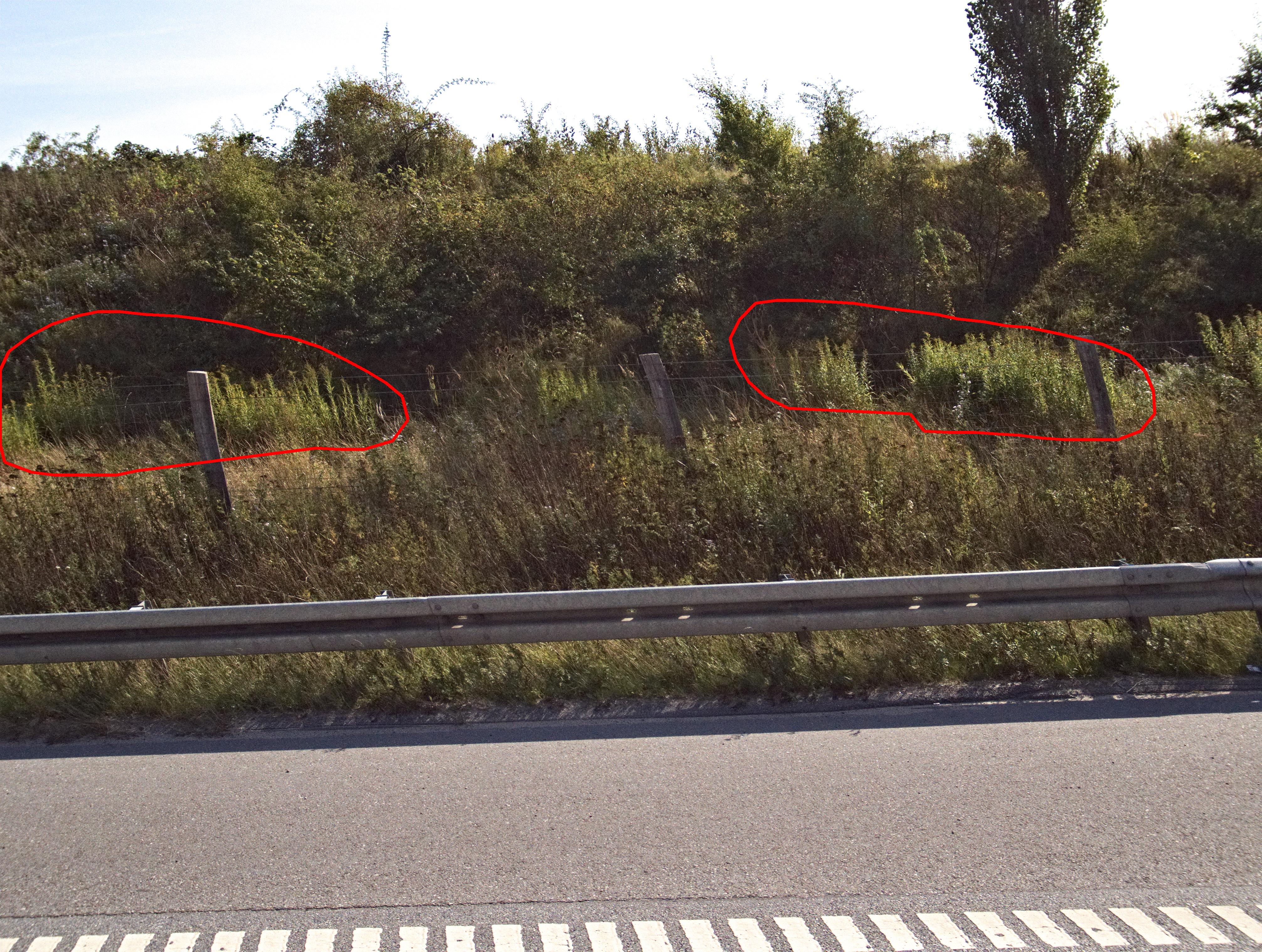

Supplement: Supplementary file 1 [file sensors-21-06126-s001.zip › images/class_examples/Gyldenris_896_0.07702631442697605_GT_2020-09-15T10_14_01.000Z_CT_1597341832.5910215_12.3243245_55.675414833.jpg]

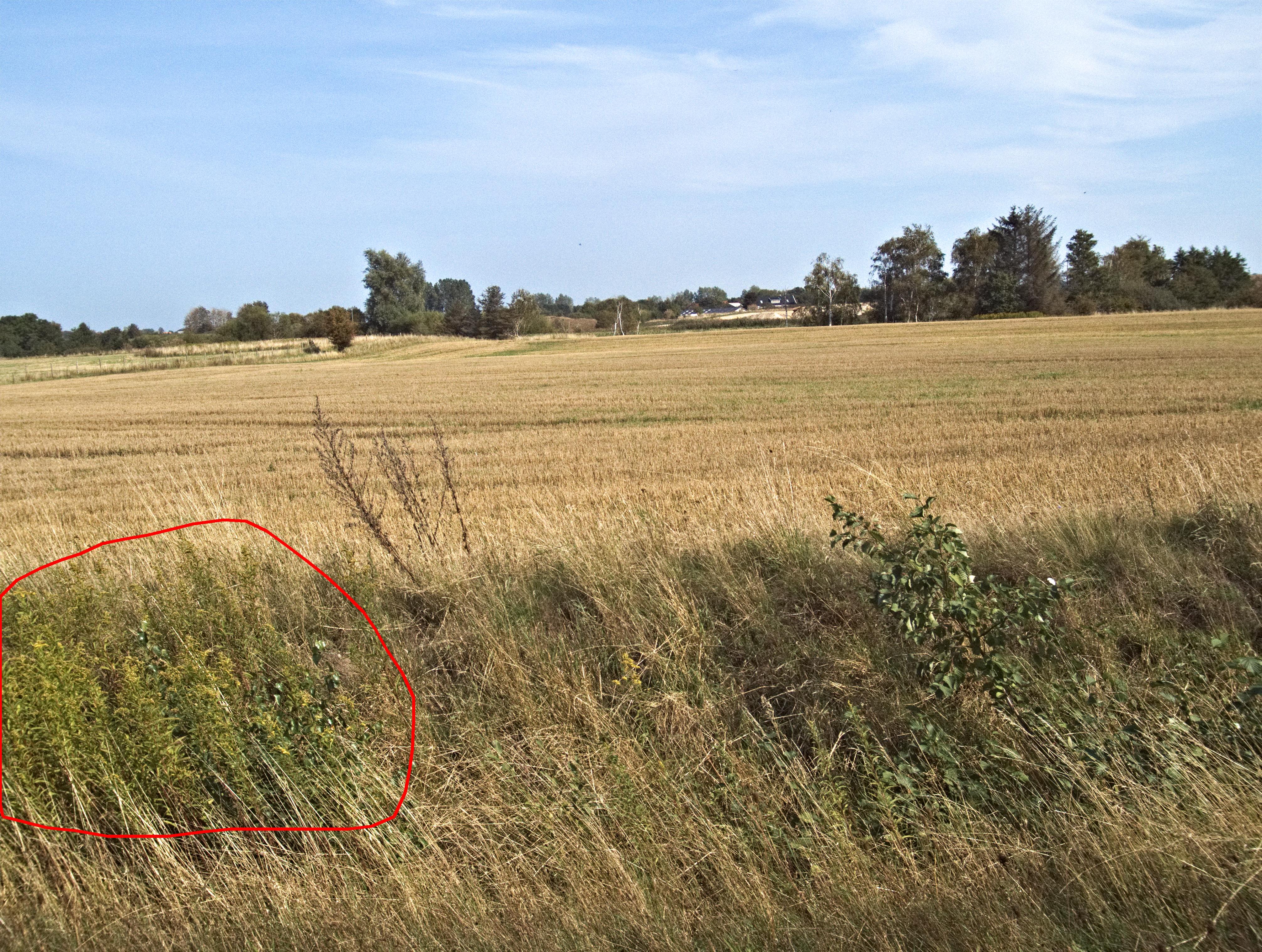

Supplement: Supplementary file 1 [file sensors-21-06126-s001.zip › images/class_examples/Gyldenris_1008_0.09071989727111923_GT_2020-09-15T11_39_29.000Z_CT_1597346961.1591063_11.566787_55.899289833.jpg]

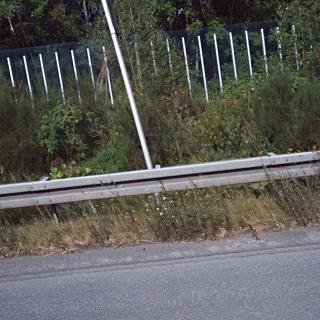

Supplement: Supplementary file 1 [file sensors-21-06126-s001.zip › images/image_size_examples/GT_2020-08-24T08_11_25.000Z_CT_1597331123.6681197_9.568677833_55.689930667__320x320.jpg]

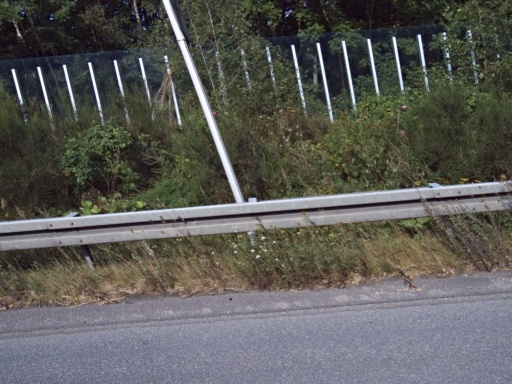

Supplement: Supplementary file 1 [file sensors-21-06126-s001.zip › images/image_size_examples/GT_2020-08-24T08_11_25.000Z_CT_1597331123.6681197_9.568677833_55.689930667__0384x0512.jpg]

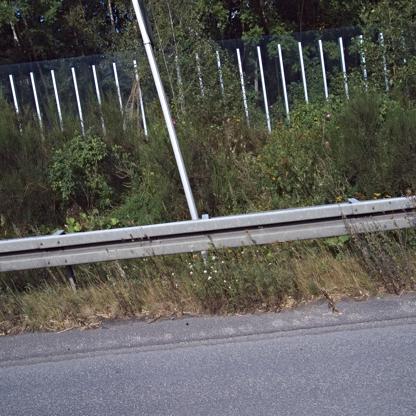

Supplement: Supplementary file 1 [file sensors-21-06126-s001.zip › images/image_size_examples/GT_2020-08-24T08_11_25.000Z_CT_1597331123.6681197_9.568677833_55.689930667__416x416.jpg]

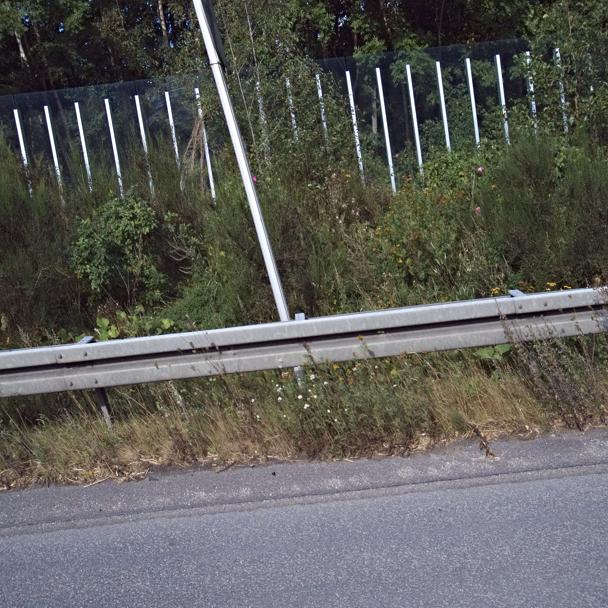

Supplement: Supplementary file 1 [file sensors-21-06126-s001.zip › images/image_size_examples/GT_2020-08-24T08_11_25.000Z_CT_1597331123.6681197_9.568677833_55.689930667__608x608.jpg]

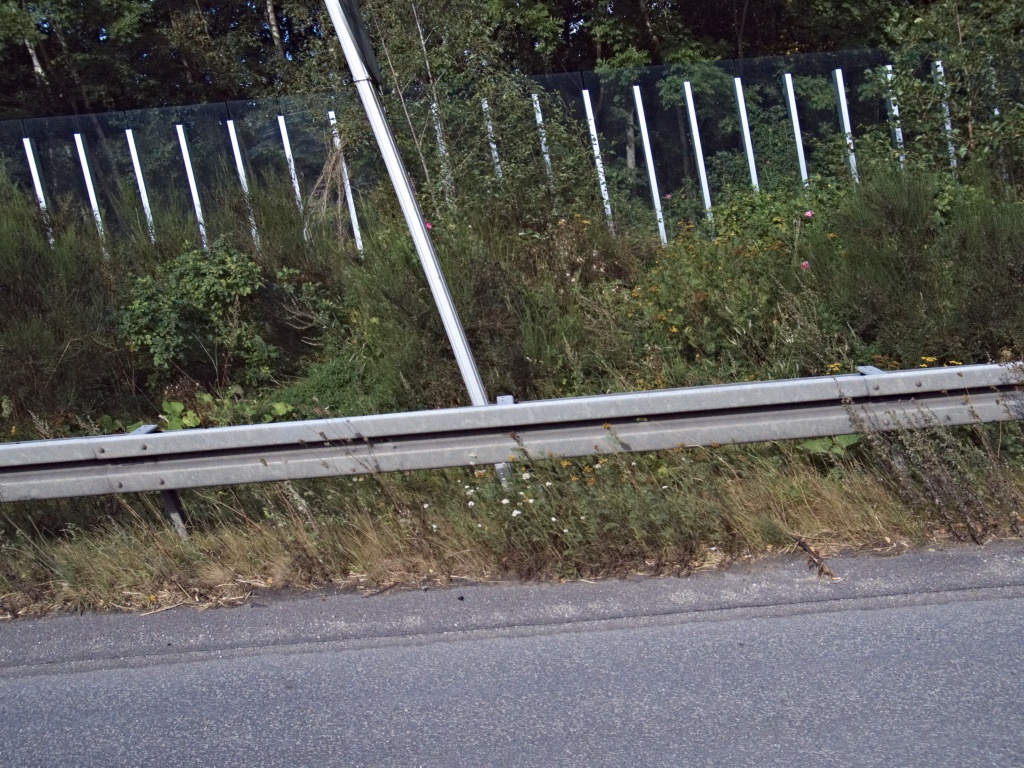

Supplement: Supplementary file 1 [file sensors-21-06126-s001.zip › images/image_size_examples/GT_2020-08-24T08_11_25.000Z_CT_1597331123.6681197_9.568677833_55.689930667__0768x1024.jpg]

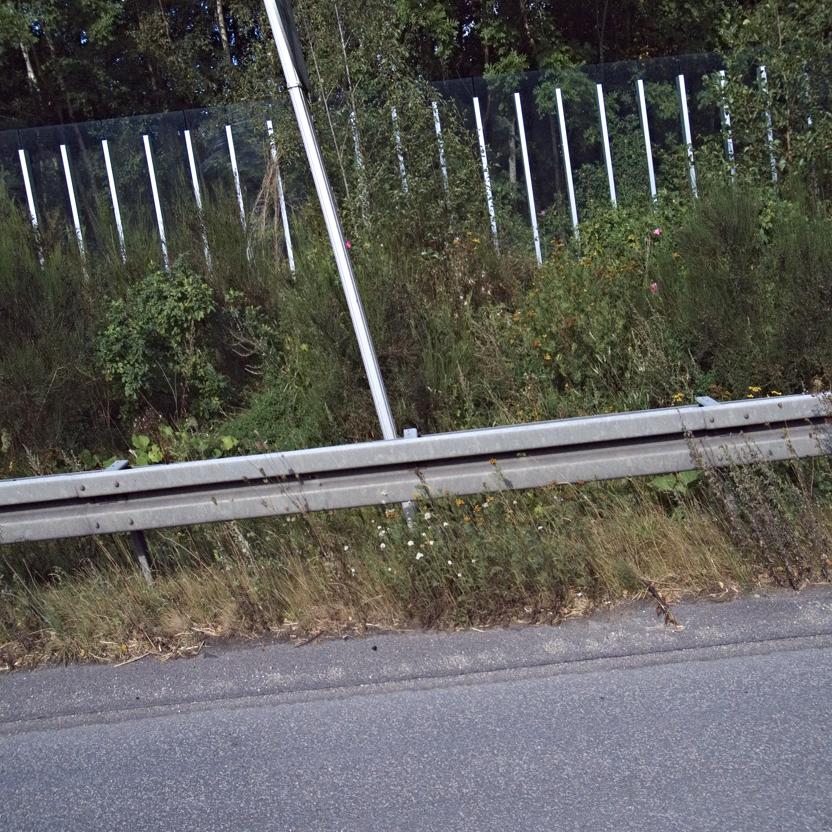

Supplement: Supplementary file 1 [file sensors-21-06126-s001.zip › images/image_size_examples/GT_2020-08-24T08_11_25.000Z_CT_1597331123.6681197_9.568677833_55.689930667__832x832.jpg]

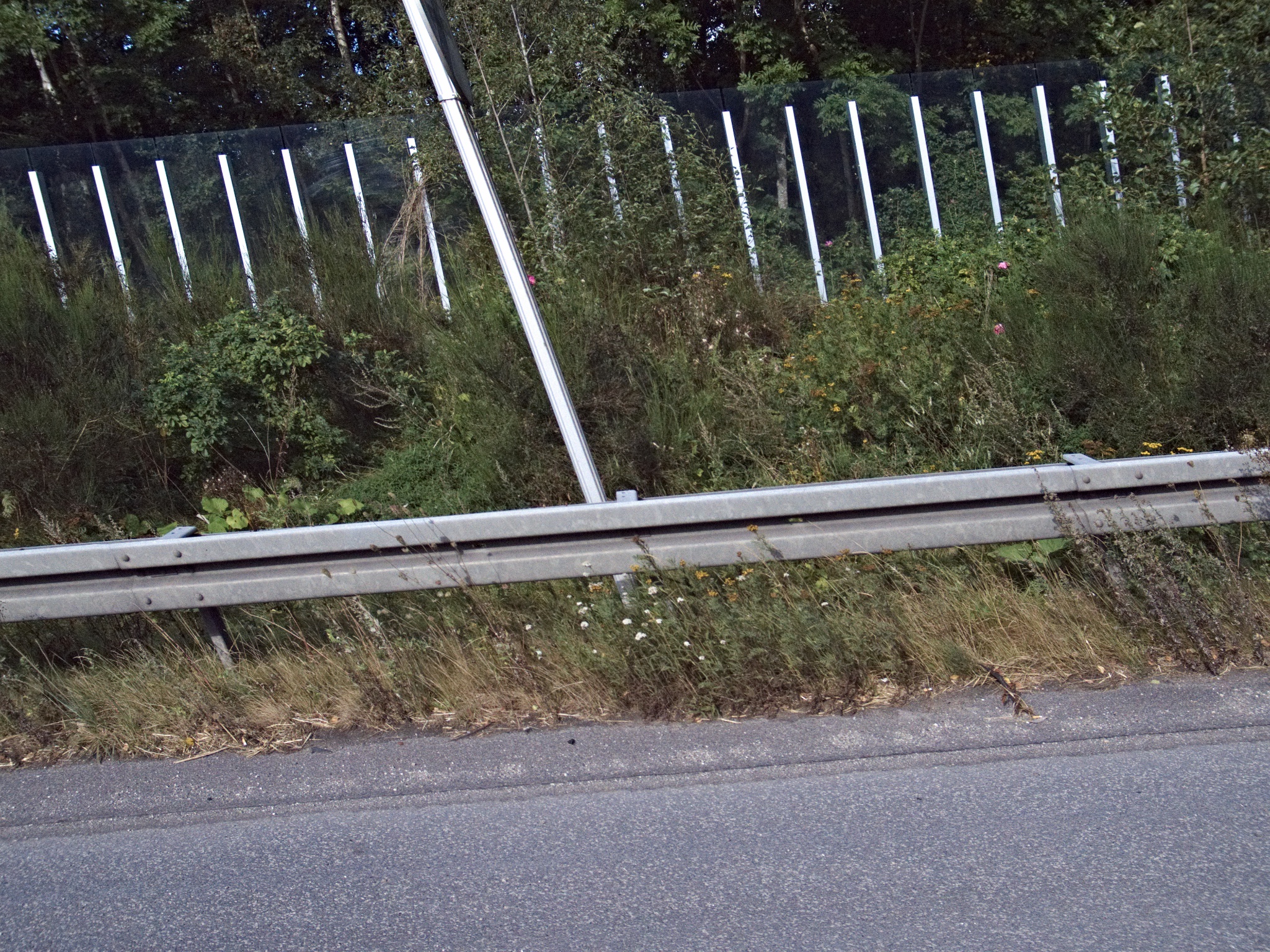

Supplement: Supplementary file 1 [file sensors-21-06126-s001.zip › images/image_size_examples/GT_2020-08-24T08_11_25.000Z_CT_1597331123.6681197_9.568677833_55.689930667__1536x2048.jpg]

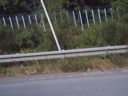

Supplement: Supplementary file 1 [file sensors-21-06126-s001.zip › images/image_size_examples/GT_2020-08-24T08_11_25.000Z_CT_1597331123.6681197_9.568677833_55.689930667__0096x0128.jpg]

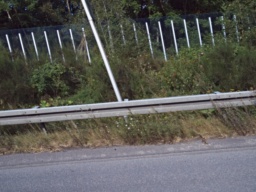

Supplement: Supplementary file 1 [file sensors-21-06126-s001.zip › images/image_size_examples/GT_2020-08-24T08_11_25.000Z_CT_1597331123.6681197_9.568677833_55.689930667__0192x0256.jpg]
